# Biophysical characterization of the calmodulin-like domain of *Plasmodium falciparum* calcium dependent protein kinase 3

**DOI:** 10.1371/journal.pone.0181721

**Published:** 2017-07-26

**Authors:** Cecilia Andresen, Markus Niklasson, Sofie Cassman Eklöf, Björn Wallner, Patrik Lundström

**Affiliations:** 1 Division of Chemistry, Department of Physics, Chemistry and Biology, Linköping University, Linköping, Sweden; 2 Division of Bioinformatics, Department of Physics, Chemistry and Biology, Linköping University, Linköping, Sweden; Griffith University, AUSTRALIA

## Abstract

Calcium dependent protein kinases are unique to plants and certain parasites and comprise an N-terminal segment and a kinase domain that is regulated by a C-terminal calcium binding domain. Since the proteins are not found in man they are potential drug targets. We have characterized the calcium binding lobes of the regulatory domain of calcium dependent protein kinase 3 from the malaria parasite *Plasmodium falciparum*. Despite being structurally similar, the two lobes differ in several other regards. While the monomeric N-terminal lobe changes its structure in response to calcium binding and shows global dynamics on the sub-millisecond time-scale both in its apo and calcium bound states, the C-terminal lobe could not be prepared calcium-free and forms dimers in solution. If our results can be generalized to the full-length protein, they suggest that the C-terminal lobe is calcium bound even at basal levels and that activation is caused by the structural reorganization associated with binding of a single calcium ion to the N-terminal lobe.

## Introduction

Malaria is a life-threatening disease caused by apicomplexan parasites of the *Plasmodium* family. Despite progress in treatment and prevention strategies there are still around 200 million cases of malaria resulting in approximately half a million deaths annually [1]. It is thus of vital interest to characterize the *Plasmodium* proteome in search for additional drug targets. For apicomplexan parasites, calcium is required for vital functions such as protein secretion, host cell invasion and parasite motility [2]. Cytosolic calcium is regulated by several mechanisms and elevated levels trigger activation of pathways that are gated by calcium sensing proteins. In plants and certain protozoa, including apicomplexan parasites, one such class of proteins is the calcium dependent protein kinases (CDPKs). In *P*. *falciparum*, there are seven CDPKs termed *pf*CDPK1 through *pf*CDPK7 [3].

**Fig 1:**
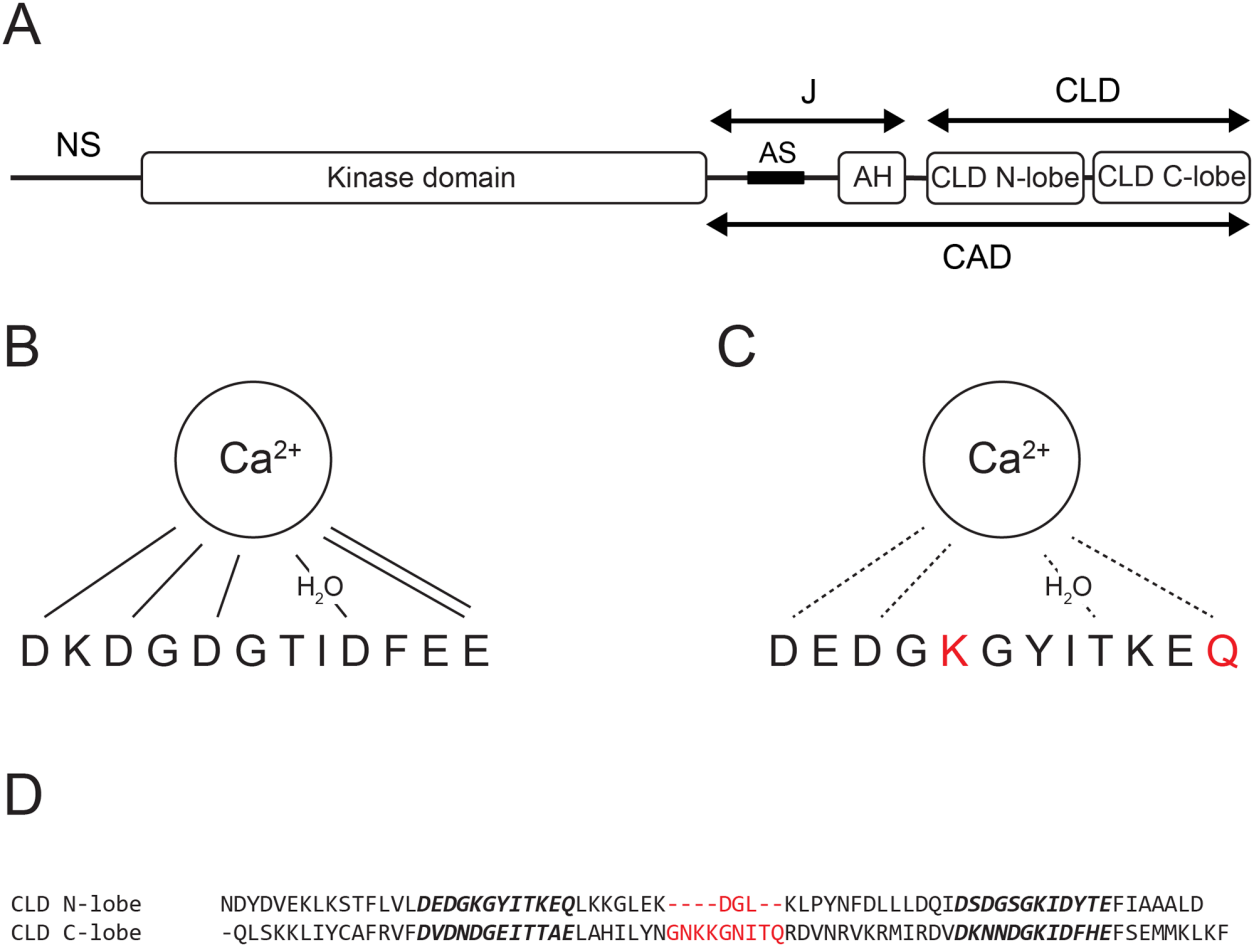
Domain organization of *pf*CDPK3 and properties of its N-terminal EF-hand loop. (A) Domain organization of *pf*CDPK3 [6]. The line represents the unstructured N-terminal sequence (NS), the black box shows the position of the autoinhibitory pseudo-substrate sequence (AS) which is followed by a regulatory helix (AH). These elements form the regulatory junction (J) that interacts with the kinase domain when the protein is inactivated. The C-terminal calmodulin like domain (CLD) consists of an N-terminal and a C-terminal lobe, each comprising a pair of EF-hands. CLD and the junction form the CDPK activation domain (CAD). (B) The consensus EF-hand calcium binding loop [10]. The residues at positions I, III and V ligate calcium using one side-chain oxygen each, the residue at position IX bridges a water ligand with its side-chain oxygen and the highly conserved glutamate at position XII ligates calcium using both side-chain oxygens as indicated by lines. A seventh ligand is provided by the backbone carbonyl at position XII. (C) The disrupted N-terminal EF-hand loop of CLD N-lobe where two substituted residues result in missing calcium ligands. These residues are highlighted in red and the failure to bind calcium is indicated by the broken lines. (D) Sequence alignment of CLD N-lobe and CLD C-lobe. The EF-hand loops are shown in bold italics and the linker between the EF-hands is highlighted in red.

The CDPK domain organization is shown in Fig 1A. The kinase domain of CDPKs is typical of Ser/Thr kinases but conserved acidic residues in the activation loop enable activation without phosphorylation of activation loop residues [4]. Instead it is regulated by the CDPK activation domain (CAD) that consists of a junction domain and a calmodulin like domain (CLD). The junction domain can be further subdivided into an autoinhibitory pseudo-substrate sequence (AS) and a regulatory helix (AH). The CLD consists of two lobes, in the following referred to as the CLD N-lobe and CLD C-lobe, respectively, where each lobe comprises a pair of EF-hands [5]. At conditions of low calcium, the AS blocks the active site of the kinase domain but upon calcium binding, the CLD sequesters the AH, which leads to release of the AH and activation [6]. Crystal structures of autoinhibited and active CDPKs from *Toxoplasma gondii* and *Cryptosporidium parvum* show that the entire CAD translocates to the opposite face of the kinase domain upon activation [7]. The length of the disordered segment upstream of the kinase domain varies among CDPKs and contains potential phosphorylation sites for known protein kinases, suggesting that function also can be regulated by phosphorylation of the N-terminal segment [8]. Crystal structures show that the CLDs of some CDPKs in their autoinhibited states coordinate magnesium and others bind other (unspecified) metal ions [7] while the CLD C-lobe from a CDPK from *Arabidopsis thaliana* has been suggested to bind calcium even at basal levels [9].

The third CDPK that was discovered in *Plasmodium falciparum*, *pf*CDPK3, is predominantly expressed during the sexual stage of the parasite life cycle [8], which is functionally and biochemically different from the asexual stage [11]. In this regard it is noteworthy that activation of gametocytes in the mosquito midgut depends on elevated calcium levels [12]. The regulatory domain of *pf*CDPK3 is special in different ways. Compared to the consensus EF-hand loop (Fig 1B) two ligands for calcium are missing because of amino acid substitutions in the N-terminal loop of CLD N-lobe (Fig 1C). This effectively mitigates calcium binding to this loop and *pf*CDPK3 can thus only bind three calcium ions, one in the N-lobe and two in the C-lobe. A further difference between the lobes is that the linker between the EF-hands of CLD N-lobe is significantly shorter than the corresponding linker of CLD C-lobe (Fig 1D).

The crystal structure of calcium bound CAD (PDB ID: 3K21) revealed an activated state with the two lobes oriented side by side [13]. The autoregulatory helix interacts primarily with the CLD C-lobe where a hydrophobic cleft is found. As expected, the first calcium binding loop of CLD N-lobe is unoccupied. The overall structure of this lobe does not seem to be affected by the shortened linker between the EF-hands except that the fold is slightly more compact than that of CLD C-lobe.

Despite their potential clinical value as drug targets [14], CDPKs have remained poorly characterized and most biophysical studies on how the proteins behave in solution have focused on CDPKs from plants rather than from parasites [9,15,16]. For *pf*CDPK3, the focus of this study, no solution structures are available. Also, there are no reports on the calcium binding properties of the two lobes and the dynamical properties of the protein. To address these questions we have calculated the solution structures, measured calcium affinity and performed detailed dynamical characterization on different time-scales of CLD from *pf*CDPK3. In order to deconvolute the properties of the two lobes, the studies have been performed on isolated CLD N-lobe and CLD C-lobe, respectively.

## Materials and methods

### Protein expression and purification

A gene encoding *pf*CDPK3 was purchased from GenScript. The fragments for CLD N-lobe (residues 416–484), CLD C-lobe (residues 487–562) and entire CLD (residues 416–562) were subcloned into a pNIC28-Bsa4 vector. The constructs were transformed into BL21(DE3) cells and expressed in M9 medium supplemented with 0.5 g/l NH_4_Cl and 2 g/l glucose. Expression and purification of the two lobes and intact CLD are detailed in S1 Text.

### Circular dichroism spectroscopy

Thermal stability of CLD C-lobe was measured by circular dichroism (CD) spectroscopy at 222 nm using a ChiraScan spectrometer (Applied Photophysics Ltd). Sample conditions were 2–4 μM protein in 4 μM Tris pH 7.1, 0.3 mM NaCl and 20 μM CaCl_2_. The temperature was raised from 16°C to 94°C in 1°C increments and at each temperature the sample was equilibrated for one minute before measurements. The data was analyzed using CDpal [17].

### NMR spectroscopy

All experiments were recorded at 25°C using Varian Inova 500 MHz and 600 MHz spectrometers equipped with cryogenically cooled probe heads. Protein concentrations were 0.5–0.8 mM in 20 mM Tris pH 7.1, 150 mM NaCl, 100 μM NaN_3_ and 10% D_2_O. 10 mM CaCl_2_ was added for CLD N-lobe^Ca^ and CLD C-lobe^Ca^ and 4 mM TCEP was added for CLD C-lobe^Ca^.

HNCACB, CBCA(CO)NH, HN(CA)CO, HNCO, HBHA(CO)NH [18] experiments were recorded to assign the protein backbone. Calcium binding to CLD N-lobe was analyzed by recording ^15^N-HSQC experiments at different concentrations of CaCl_2_.

^15^N *R*_1_, ^15^N *R*_1*ρ*_ and {^1^H}-^15^N NOE experiments at were recorded at 600 MHz using standard pulse sequences [19,20]. For measurement *R*_1_, experiments with relaxation delays in the range 20–645 ms and 20–800 ms were acquired for CLD N-lobe and CLD C-lobe, respectively. *R*_1*ρ*_ was measured by recording experiments with relaxation delays in the range 6–100 ms for both lobes. The amplitude of the spin lock fields was ≈1800 Hz and they were centered at 119 ppm. The heteronuclear NOE was measured by recording experiments with or without a 5 s period of saturation pulses. The total recovery delay was 12 s.

^15^N (and ^13^CO for CLD N-lobe^Ca^) CPMG [21,22] relaxation dispersion experiments [23,24] were recorded using effective fields of 33–1000 Hz and for CLD N-lobe^apo^, also ^1^HN *R*_1*ρ*_ relaxation dispersions were recorded [25]. On-resonance experiments employed spin lock field strengths in the range 738–14535 Hz with the spinlock carrier centered at 8.5 ppm. Off-resonance experiments were recorded at a spin lock field strength of 2115 Hz with nominal tilt angles between 11.9° and 64.5°.

### Isothermal calorimetry

Purified proteins were dialyzed twice against 4 mM EDTA and then three times against a buffer containing 20 mM HEPES, pH 7.1, 150 mM NaCl. Titrations were performed using a MicroCal PEAQ-ITC system (Malvern Instruments Ltd) at 25°C using 37 injections of 1 μl (first injection 0.8 μl) of ligand in 20 mM HEPES pH 7.1, 150 mM NaCl and a delay of 100 s between injections. The concentration of the ligand was 3 mM (CLD N-lobe) or 2 mM (CLD). The data was fitted to a one set of sites site model. The experiments were performed in triplicate and the results are reported as the mean ± the standard deviation of these.

### Data analysis

All NMR data were processed with NMRpipe [26] and visualized in Sparky (Gordon and Kneller, University of California, San Francisco). Backbone resonances were assigned using the software COMPASS [27]. Peaks were integrated and relaxation rate constants were fitted using PINT [28]. Uncertainties in peak volumes were estimated from duplicate data points. *R*_2_ was calculated from *R*_1*ρ*_ and *R*_1_ as

$$R_{2}=R_{1\rho }/{\text{sin}}^{2}\theta -R_{1}/{\text{tan}}^{2}\theta$$ (1)

where *θ* = arctan(*B*_1_/Ω) is the tilt angle of the effective field, $\omega_{eff}=2\pi {\left[B_{1}^{2}+\Omega^{2}\right]}^{½}$, with respect to the static magnetic field, where *B*_1_ is the spin lock field strength in frequency units and Ω the resonance offset from the radio frequency carrier [29]. Errors in the fitted parameters were estimated by the jack-knife approach [30].

Peak volumes from ^15^N CPMG relaxation dispersion experiments were converted into effective transverse relaxation rates *R*_2,*eff*_ (*v*_*cpmg*_) = ln(*I*_0_/*I*)/*T*, where *I* and *I*_0_ are the intensities with and without the constant time relaxation delay of duration *T* and *v*_*cpmg*_ is the repetition rate in the CPMG pulse train. Residues with significant chemical exchange (*p*<0.01) in individual fits were fitted to a global two-state model using the software CATIA [31].

*R*_1*ρ*_ relaxation dispersions were fitted on a per-residue basis to the expression for two-state dynamics in the fast exchange limit

$$R_{1\rho }=R_{1}{\text{cos}}^{2}\theta +R_{2,0}{\text{sin}}^{2}\theta +k_{ex}\phi_{ex}/\left(k_{ex}^{2}+\omega_{eff}^{2}\right){\text{sin}}^{2}\theta$$ (2)

where *k*_*ex*_ is the exchange rate constant and *ϕ*_*ex*_ = *p*_*B*_(1 − *p*_*B*_)Δ*ω*^2^ in which *p*_*B*_ is the population of the alternative state and Δ*ω* is the difference in resonance (angular) frequency between the two states [32]. The data was also fitted to a model excluding dynamics by omitting the last term in Eq 2. The exchange rates for residues with significant exchange (*p*<0.01) were clustered using k-means where each residue was represented as a gaussian centered at *k*_*ex*_ and with a width corresponding to the uncertainty in *k*_*ex*_.

### Structural ensembles

CS-Rosetta [33] was used to calculate structural ensembles guided by NMR chemical shifts to generate local backbone fragments. To avoid including existing crystal structure fragments with sequence similarity to our target structure, all fragments were generated with the *-nohoms* flag. The calcium binding sites were modeled by restraining the pairwise distances for the coordinating residues across the binding site to distances obtained from known calcium binding sites. In total 83176, 86711, and 70698 structural models were sampled for CLD N-lobe^apo^, CLD N-lobe^Ca^ and CLD C-lobe^Ca^, respectively. Models were clustered using 3 Å cutoff using Rosetta’s clustering application and the lowest energy model with a chemical shift root-mean-squared difference between experimental and chemical shifts predicted by SPARTA+ [34] better than one standard deviation from the ensemble mean was selected as the structural representative. The generated structures were used in hydrodynamic calculations of correlational times for rotational diffusion [35].

## Results

### The two isolated CLD lobes of *pf*CDPK3 are well-folded and CLD C-lobe cannot be prepared calcium-free

**Fig 2:**
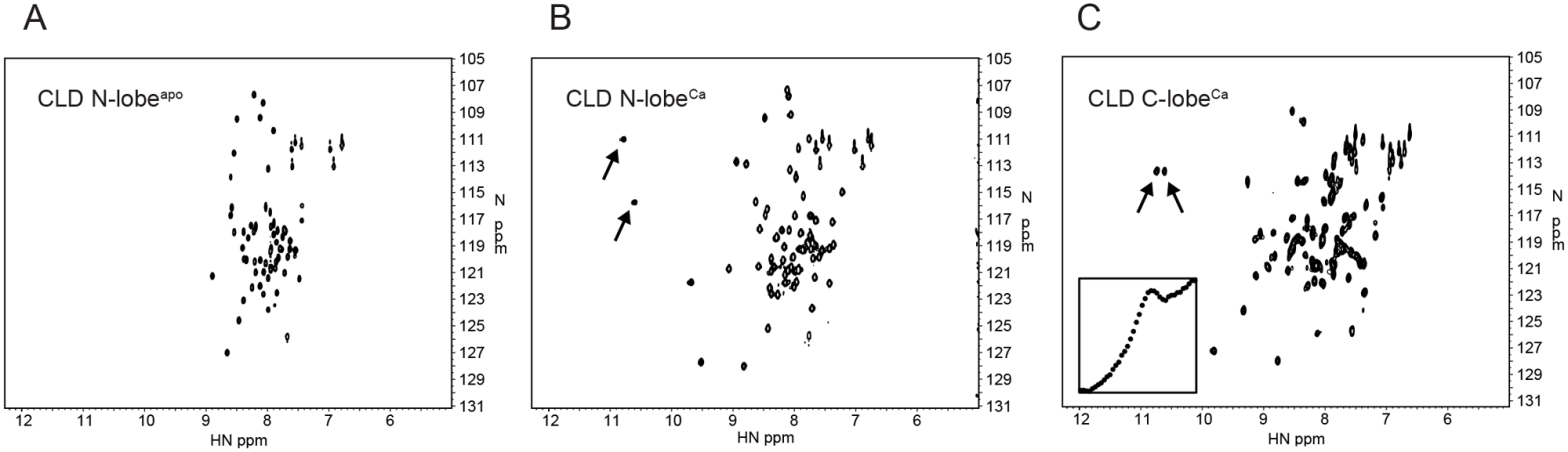
^15^N-HSQC spectra for (A) CLD N-lobe^apo^, (B) CLD N-lobe^Ca^ and (C) CLD C-lobe^Ca^. The inset in panel C shows a circular dichroism thermal denaturation profile for CLD C-lobe^Ca^ in the temperature range 16–94°C.

We were not able to obtain samples of intact CLD or indeed CAD in sufficient concentrations and at conditions suitable for NMR spectroscopy. Therefore most experiments involved the two CLD domains in isolation. Furthermore, since calcium could not be removed from CLD C-lobe we were not able to perform any studies of its apo state. The ^15^N-HSQC spectra of CLD N-lobe in its apo state (CLD N-lobe^apo^), CLD N-lobe in the presence of calcium (CLD N-lobe^Ca^) and CLD C-lobe in the presence of calcium (CLD C-lobe^Ca^) shown in Fig 2 demonstrate that they all are well-folded. As expected, the chemical shift dispersion is larger for the calcium bound forms. The spectra show many hallmarks of EF-hand proteins, the most obvious being that the amide proton resonances of the glycine residues at position VI of the calcium binding loops resonate above 10.5 ppm in the calcium bound forms. This also applies to G436 in the N-terminal loop of CLD N-lobe that is expected to be unoccupied [13] and we point out that these chemical shifts do not reflect calcium-binding *per se* but formation of hydrogen bonds with the side-chain of the residue at position I of the loops. Evidence against calcium-binding is that the chemical shift of I438^N^ moves *upfield* by 1.1 ppm upon addition of calcium since a significant *downfield* movement upon binding is expected [36].

As reported previously, CLD N-lobe undergoes two-state unfolding with denaturation midpoint temperatures of 64.4 ± 0.4°C and 90 ± 3°C for CLD N-lobe^apo^ and CLD N-lobe^Ca^, respectively [17]. In contrast, the thermal denaturation profiles for several samples of CLD C-lobe^Ca^ were not compatible with two-state denaturation (see inset of Fig 2C and S1 Fig) and it was not fruitful to estimate the midpoint temperature of denaturation. Purification of CLD C-lobe in the absence of calcium was not possible and the NMR spectrum did not change even after extensive dialysis against EDTA while direct addition of EDTA inevitably resulted in protein precipitation. It was thus not possible to study CLD C-lobe in the absence of calcium.

### Structural ensemble from chemical shifts

The resonances were assigned by recording the standard suite of triple-resonance experiments [18] and using the semi-automatic assignment software COMPASS [27]. The assignment completeness for amide moieties were 97.2%, 97.2% [*sic*] and 92.2% for CLD N-lobe^apo^, CLD N-lobe^Ca^ and CLD C-lobe^Ca^, respectively. Unassigned resonances were mostly localized to the N-termini. Additionally, for CLD N-lobe^apo^ as well as CLD N-lobe^Ca^, Y456 that follows the only proline residue in the amino acid sequence and for CLD C-lobe^Ca^, F500 and K548 could not be identified. The chemical shifts were used to predict the secondary structure, which yielded the classical EF-hand helix-loop-helix-linker-helix-loop-helix motif.

**Fig 3:**
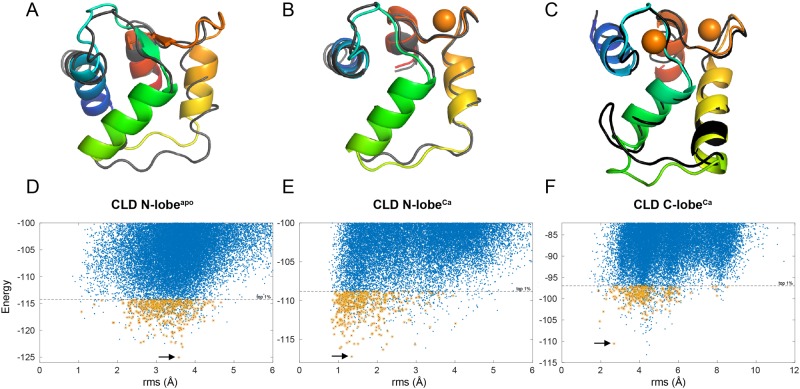
Selected models generated by CS-Rosetta using chemical shifts as restraints of (A) CLD N-lobe^apo^, (B) CLD N-lobe^Ca^ and (C) CLD C-lobe^Ca^ overlaid with the corresponding fragments of the CAD crystal structure (PDB ID: 3K21). The models are colored as a rainbow (blue N-terminus and red C-terminus) while the crystal structure is in dark gray with calcium ions shown as orange spheres. (D-F) The corresponding RMSD vs. Rosetta Energy scatter plots for models generated for CLD N-lobe^apo^, CLD N-lobe^Ca^ and CLD C-lobe^Ca^, respectively. The RMSD is calculated against the corresponding fragment of the CAD crystal structure. The dashed line shows the cutoff for the 1% lowest energy models, circled points (orange) indicate models that are among the 1% lowest energy and have a chemical shift RMSD that is one standard deviation better than the ensemble mean. The arrows point to the selected models shown in (A-C).

CS-Rosetta [33] was used to generate structural ensembles. After structural clustering, the lowest energy model that also had predicted chemical shifts that agreed well with the experimentally observed chemical shifts was selected (Fig 3A–3C). For CLD N-lobe^apo^ and CLD N-lobe^Ca^ the selected structural model was also the lowest energy structural representative from the largest cluster. For CLD C-lobe^Ca^, the lowest energy model did not agree well with experimental chemical shifts, thus the third lowest energy model (depicted by the arrow in Fig 3F) was selected as the structural representative. Coordinates for the selected structural models for CLD N-lobe^apo^, CLD N-lobe^Ca^ and CLD C-lobe^Ca^ are presented in S1–S3 Tables. As hinted by the secondary structure pattern predicted by the chemical shifts, the selected models are similar to EF-hand domains. The calcium bound models are similar to their corresponding fragment of the CAD crystal structure (PDB ID: 3K21) (Fig 3E and 3F). For CLD N-lobe^Ca^ the agreement is especially good with an RMSD of 1.3 Å. The hydrogen bonds that explain the high chemical shifts of the amide protons of the glycines at loop position VI were readily identified, also for the loop carrying substitutions. The RMSD of 2.5 Å for CLD C-lobe^Ca^ is slightly larger with regions of poor agreement largely confined to the linker between the two EF-hands. CLD N-lobe^apo^ shows larger RMSD, above 3.5 Å, for the selected representative (Fig 3D). The larger RMSD for the apo structure is mostly attributed to the different orientation of the N-terminal helix and the linker connecting helices 2 and 3. The differences can be summarized as that the CLD N-lobe^apo^ adopts a more compact structure, with smaller angles between the helices of the first EF-hand like other calcium-sensing EF-hand proteins in their apo states [37]. Apparently, binding of a single calcium ion to the C-terminal loop is sufficient for triggering the conformational switch.

### Only CLD N-lobe responds to changes in calcium concentration

**Fig 4:**
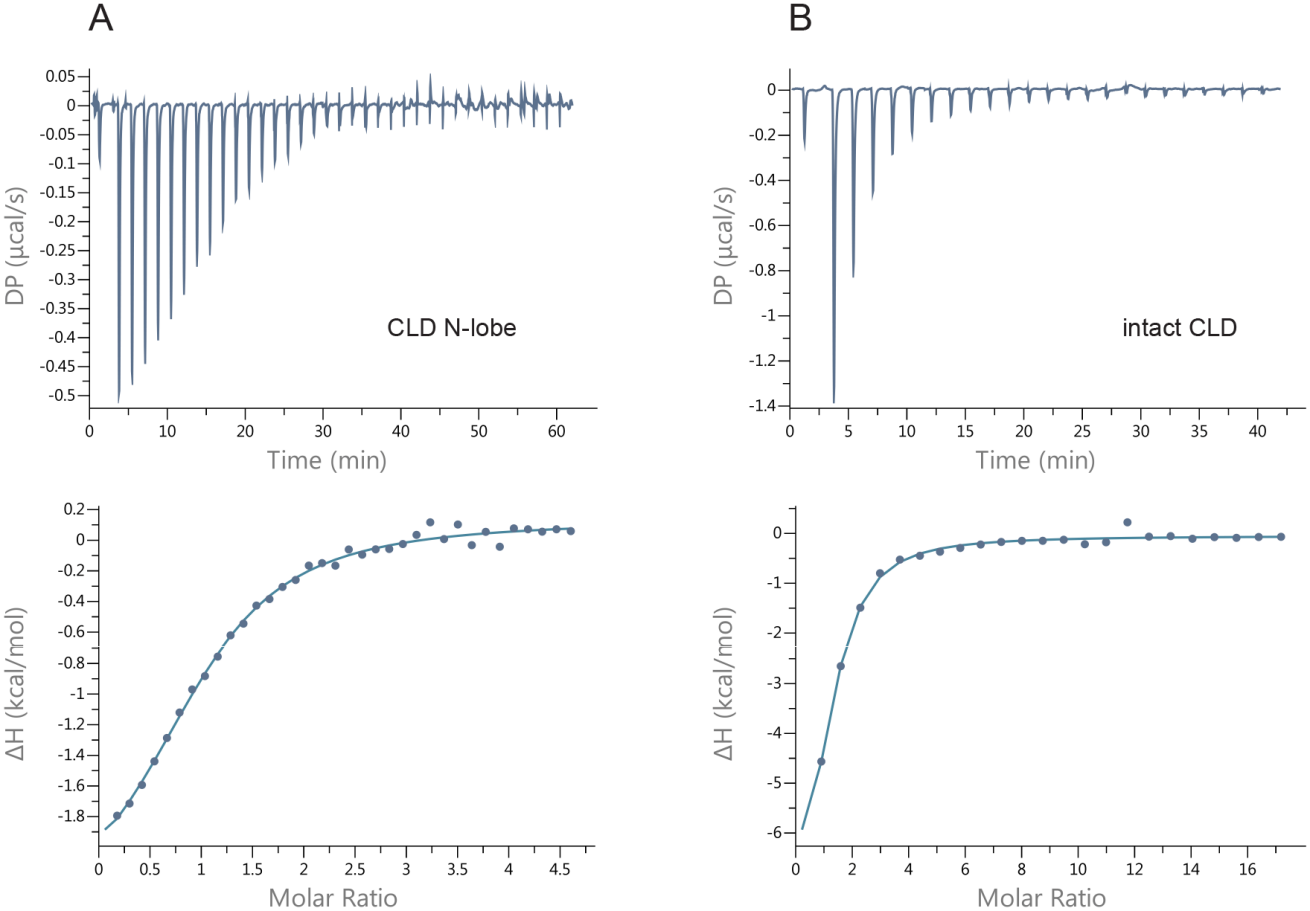
Calcium affinity of (A) CLD N-lobe and (B) CLD analyzed by isothermal calorimetry. The experiments were recorded in triplicates and representative profiles are shown. The data was fitted to a one set of sites binding model. The dissociation constants were 38 ± 3 μM and 18 ± 2 μM for CLD N-lobe and intact CLD, respectively.

The calcium dissociation constant of CLD N-lobe was determined by isothermal calorimetry (ITC). The data was well fitted to one-site model with a stoichiometric ratio of 1:1 and yielded a a K_d_ of 38 ± 3 μM (Fig 4). As expected from the literature [13] and our NMR data, the N-lobe thus only binds one calcium ion with substantial affinity. As mentioned, we could not prepare calcium-free CLD C-lobe but we were able to purify intact CLD at concentrations suitable for ITC. After extensive dialysis against EDTA and then buffer its ITC profile resembled that of CLD N-lobe and could be fitted to a one-site 1:1 model with K_d_ = 18 ± 2 μM (Fig 4). Importantly, attempts to fit the data to 2:1 or 3:1 (calcium:protein) ratios were only possible if the protein concentration was set fixed to unrealistically low values. Our interpretation is that also in the context of full length CLD, CLD C-lobe remains calcium bound even at low calcium concentrations and that the experiments reflected binding of an additional calcium ion to the N-lobe.

### CLD C-lobe^Ca^ forms dimers in solution

**Fig 5:**
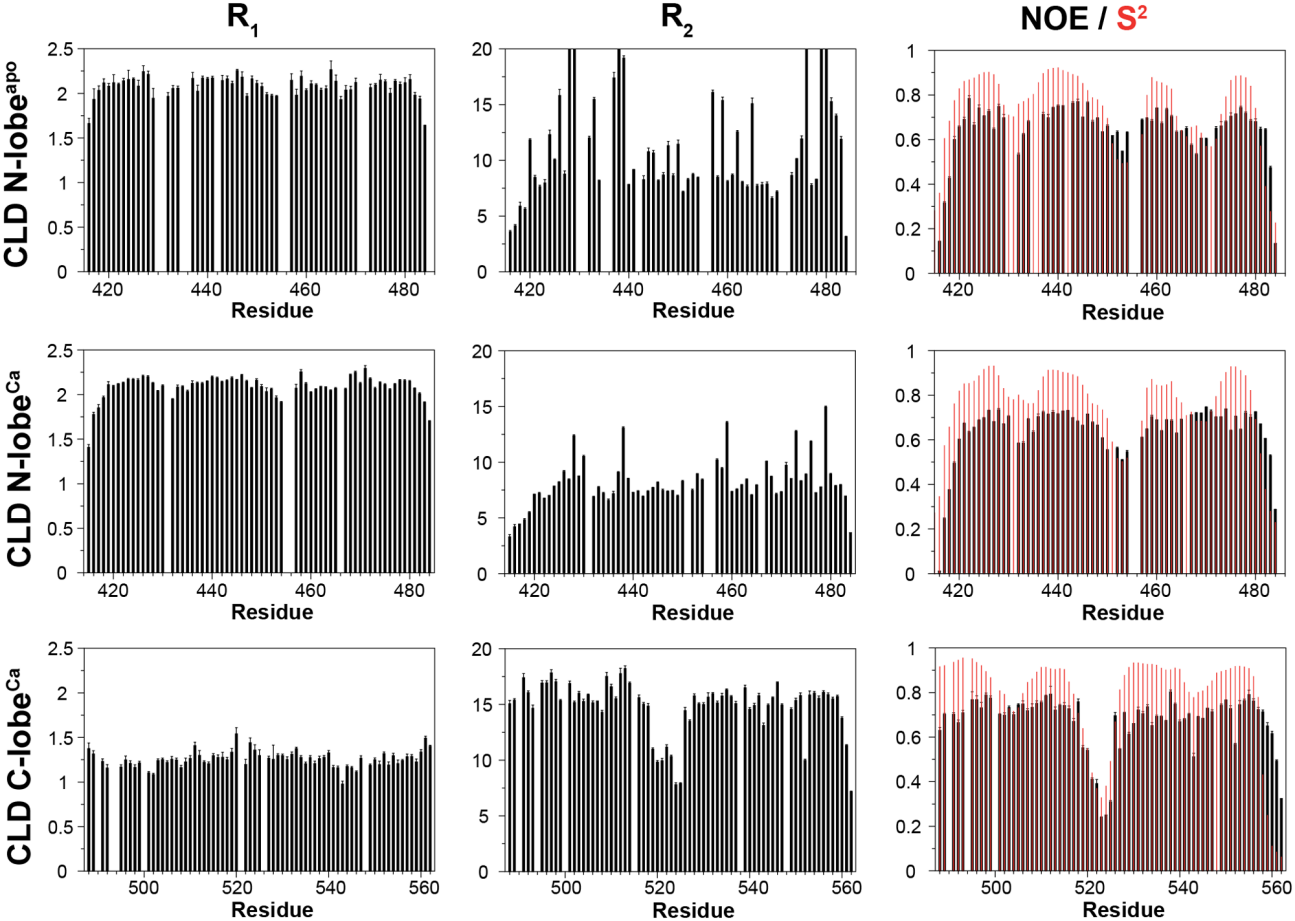
^15^N relaxation rate constants and the {^1^H}-^15^N NOE for CLD N-lobe^apo^, CLD N-lobe^Ca^ and CLD C-lobe^Ca^. Order parameters, calculated by the RCI method and shown as red bars, have been included in the panels representing the heteronuclear NOE. The vertical axes have been scaled identically for all proteins.

To characterize molecular tumbling and backbone flexibility, we measured *R*_1_, *R*_2_ and the heteronuclear NOE. The most striking feature of the relaxation data is that *R*_1_ and *R*_2_ are very different for CLD N-lobe^apo/Ca^ and CLD C-lobe^Ca^ (Fig 5). The ratio of these parameters were used to calculate correlation time for molecular reorientation [38]. The correlation times of 5.3 ± 0.1 ns and 5.9 ± 0.4 ns for CLD N-lobe^apo^ and CLD N-lobe^Ca^, respectively, are fully compatible with the domains tumbling as monomers and the slightly larger correlation time for CLD N-lobe^Ca^ is consistent with its more expanded structure. In contrast, the estimated correlation time of 15.0 ± 0.1 ns for CLD C-lobe^Ca^ can only be explained by formation of dimers in solution. These results are corroborated by hydrodynamic calculations [35] based on the calculated structures from which we obtained rotational correlation times of 5.0 ns, 5.2 ns and 7.1 ns for monomeric CLD N-lobe^apo^, CLD N-lobe^Ca^ and CLD C-lobe^Ca^, respectively.

### CLD N-lobe^apo^ and CLD N-lobe^Ca^ exchange with alternative states

**Fig 6:**
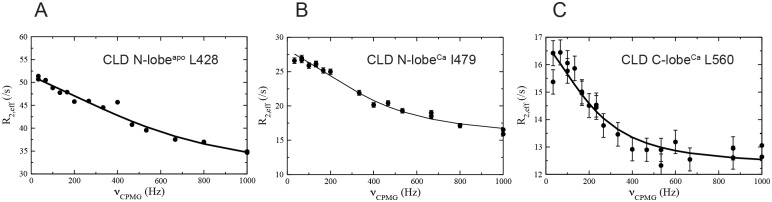
Examples of dispersion profiles for (A) CLD N-lobe^apo^, (B) CLD N-lobe^Ca^ and (C) CLD C-lobe^Ca^. The lines represent the best fit to a local two-state process for CLD N-lobe^apo^ and to a global two-state process for CLD N-lobe^Ca^ and CLD C-lobe^Ca^. The data was recorded at a static magnetic field of 14.1 T at 25°C. The values of the fitted global exchange parameters are presented in the text. Dispersion profiles for all residues with μs–ms dynamics can be found in S2–S4 Figs.

Relaxation dispersions were measured to characterize μs–ms motions. These experiments are sensitive to the population of an alternative state, *p*_*B*_, the exchange rate constant, *k*_*ex*_, and the magnitude of the difference in chemical shifts, |Δ*ϖ*|, between exchanging states [39]. The first two parameters report on the thermodynamics and kinetics of the exchange while |Δ*ϖ*| is related to the structure of alternative state. Examples of ^15^N CPMG dispersion profiles for CLD N-lobe^apo^, CLD N-lobe^Ca^ and CLD C-lobe^Ca^ are shown in Fig 6. There were few residues with μs—ms dynamics for CLD C-lobe^Ca^ and these residues are primarily localized to the N- and C-termini. There is no evidence that these dynamics are due to dimer-monomer exchange since the exchange rate of *k*_*ex*_ = 860 ± 90 s^-1^ and population of the alternative state of *p*_*B*_ = 1.7 ± 0.1% were unchanged when the sample was diluted two-fold. Millisecond dynamics of CLD C-lobe^Ca^ will not be discussed further herein.

**Fig 7:**
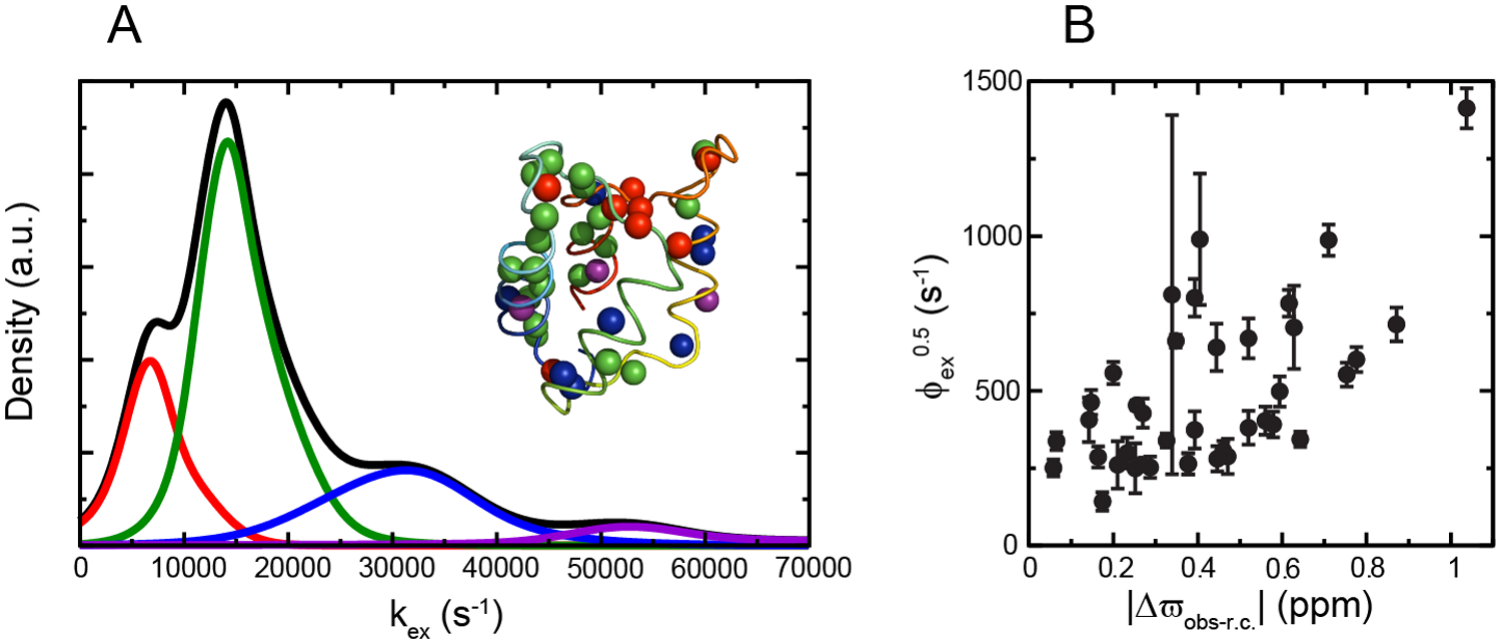
Conformational dynamics of CLD N-lobe^apo^. (A) K-means (k = 4) cluster analysis of exchange rates for CLD N-lobe^apo^. The mean values of the clusters are 6900 s^-1^ (n = 8; red), 15400 s^-1^ (n = 20; green), 30800 s^-1^ (n = 8; blue) and 57100 s^-1^ (n = 3; violet). The black line represents the sum of the four clusters. The inset shows the structure of CLD N-lobe^apo^ (colored as in Fig 3) with exchanging amide protons as spheres, color coded according to cluster identity. (B) Correlation between $\phi_{ex}^{0.5}$ and magnitude of differences between observed chemical shifts and expected chemical shifts for a random coil.

In contrast, conformational dynamics was detected for the majority of the residues of CLD N-lobe in both its apo and calcium bound states. For CLD N-lobe^apo^, the exchange was not quenched at the highest effective field and we therefore also recorded ^1^HN *R*_1*ρ*_ dispersions and identified chemical exchange for 39 residues. The dispersions were sizable with an average of 19 ± 30 s^-1^ and in two cases (K435 and I473) they exceeded 100 s^-1^ (S2 Fig). The exchange rates spanned the range 4300–63000 s^-1^ and the data could not be fitted to a global two-state process. Instead we used the k-means approach [40] to cluster the individual apparent two-state exchange rates into four groups and residues belonging to the respective groups were color coded onto the structure of CLD N-lobe^apo^ (Fig 7A) to gain insight into their spatial distribution. Exchange is most abundant in helices 1 and 4 where most residues are in the class with an exchange rate centered at 15400 s^-1^ and in the calcium binding loops where most residues are in the class with and average exchange rate of 6900 s^-1^. The square root of the exchange parameter *ϕ*_*ex*_ is proportional to |Δ*ϖ*| (see Eq 2) and can thus be used as a proxy for the ‘amplitude’ of the structural fluctuations at different positions to provide insight into the nature of the alternative state. When compared with the difference between the observed chemical shifts and expected random coil chemical shifts a descent correlation with Pearson’s coefficient of correlation of 0.60 was obtained (Fig 7B).

**Fig 8:**
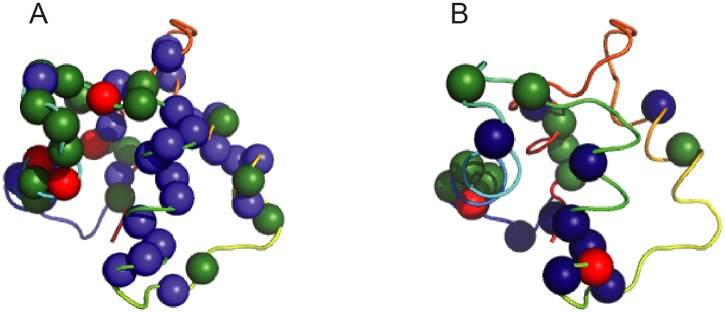
Spatial distribution of exchanging backbone ^15^N and ^13^CO nuclei of CLD N-lobe^Ca^. Exchanging nuclei are shown as spheres on the structure of CLD N-lobe^Ca^ colored as in Fig 3. (A) Exchanging backbone ^15^N nuclei. The color coding is |Δ*ϖ*_*CPMG*_| <2 ppm (indigo), 2 < |Δ*ϖ*_*CPMG*_| < 3 ppm (green) and |Δ*ϖ*_*CPMG*_| > 3 ppm (red). (B) Exchanging backbone ^13^CO nuclei. The color coding is |Δ*ϖ*_*CPMG*_| <0.75 ppm (indigo), 0.75 < |Δ*ϖ*_*CPMG*_| < 1.0 ppm (green) and |Δ*ϖ*_*CPMG*_| > 1.0 ppm (red).

For CLD N-lobe^Ca^ the ^15^N CPMG experiment revealed millisecond dynamics for almost all residues. In addition of the N-terminal residues G415-D419, the only exceptions for assigned residues were K422, L452, G469, S470, G471 and D484. The dynamics differed from that of the apo state of the domain in two regards. First, the exchange is considerably slower and second, the process is simpler since the data could be satisfactory fitted to a global two-state model with *k*_*ex*_ = 1640 ± 70 s^-1^ and *p*_*B*_ = 1.13 ± 0.04%. The average |Δ*ϖ*_*CPMG*_| was 2.1 ± 1 ppm and largest values were found for residues L428 (4.4 ppm), I438 (5.1 ppm), I479 (5.9 ppm). We also recorded ^13^CO relaxation dispersions that mainly are sensitive to modulation of the backbone dihedral angles and identified dynamics for 21 residues (Fig 8B). Exchange was particularly abundant in helices 1, 2 and 4 and in the unoccupied calcium binding loop. It is noteworthy that we did not identify a single residue with ^13^CO dispersions in the second, occupied, calcium binding loop.

## Discussion

Reassuringly, the individual domains of CLD adopt solution structures that are typical of EF-hand proteins and the calcium bound forms are very similar to the corresponding fragments of the crystal structure of calcium bound CAD as gauged by the CS-Rosetta structural ensembles. It is thus valid to study the lobes in isolation for many purposes although we refrain from interpretating the details of the structures. It is however noteworthy that binding of a single calcium ion to the N-lobe is sufficient for triggering the structural change associated with activation of EF-hand domains [41]. The larger deviation between the solution and crystal structures of CLD C-lobe^Ca^ than of CLD N-lobe^Ca^ may in part be explained by our observation that the isolated fragment of the former forms dimers in solution.

The failure to remove calcium from CLD C-lobe and our ITC results for intact CLD point to a very high calcium affinity for the lobe. If the results can be generalized to full-length *pf*CDPK3 they could mean that CLD C-lobe binds calcium immediately after synthesis and thus does not change its structure in response to fluctuating calcium levels as has been proposed for CPK-1 from *Arabidopsis thaliana* [9]. Another possibility is that magnesium or other metal ions occupy the loops of CLD C-lobe at low calcium levels as has been suggested for other apicomplexan CDPKs [7]. Regardless, activation of *pf*CDPK3 would then be due to the structural reorganization associated with calcium binding to the CLD N-lobe. The dissociation constant for this binding is approximately two orders of magnitude lower than for calcium binding to calmodulin at similar conditions [42] and since only one of the CLD N-lobe loops binds calcium there can be no cooperativity within the lobe. This means that very high levels of calcium are needed for the activation of *pf*CDPK3 although the dissociation constant is lowered by a factor two in the context of intact CLD and possibly more for full length *pf*CDPK3. We stress that future studies on full length *pf*CDPK3 or at least CAD are necessary to confirm these hypotheses and that mutational data would be helpful to conclusively establish the binding mechanism.

The most important finding from the analysis of the relaxation rate constants that report on molecular motions was that CLD C-lobe^Ca^ dimerizes at all concentrations tested and the instability of the protein in monomeric form explains the unusual denaturation profile shown in Fig 2. Interestingly, the software PISA [43] predicts that CAD forms dimers in the presence of calcium. However, the interaction surface between two individual CLD C-lobe units in these dimers is small and it is unlikely that the dimerization observed here takes place *in vivo*. A more plausible biological implication of our observation is thus that CLD C-lobe requires an interaction partner. In the crystal structure of intact CAD at high calcium levels, this role is fulfilled by the junction region [13]. If indeed CLD C-lobe always is calcium bound, the requirement would also apply to conditions of low calcium. *pf*CDPK3 could then once again resemble the model for CPK-1 from *Arabidopsis thaliana*, where there is interaction between CLD C-lobe and the junction region also at low calcium levels [9].

While only a few CLD C-lobe^Ca^ residues are sensitive to μs–ms motions, there is global exchange with alternative states for CLD N-lobe in its apo as well as calcium bound state. The dynamics of CLD N-lobe^apo^ are more complex than a simple two-state process since the data had to be fitted on a per-residue basis. The so obtained exchange rates could however be divided onto four clusters. For the largest cluster 16 out of 20 members were located in helix 1, helix 4 and the N-terminal part of the first loop, making it likely that this region of the protein experiences a common dominant process. Based on the correlation between $\phi_{ex}^{0.5}$ and the absolute value of the difference between observed and random coil chemical shifts a possible model is that CLD N-lobe^apo^ is in equilibrium with a sizable fraction of a conformation where the lobe is partially unfolded although the less than perfect correlation and the failure to fit the data to a two-state process demonstrates additional processes.

We found no evidence for a ‘conformational selection’ model for calcium binding since there was no correlation between $\phi_{ex}^{0.5}$ and the magnitude of the difference between CLD N-lobe^apo^ and CLD N-lobe^Ca^ chemical shifts and on the same grounds we ruled out that dynamics for CLD N-lobe^Ca^, are the result of exchange with the apo state. Additional evidence against this is that there were ^13^CO dispersions for most regions of CLD N-lobe^Ca^ but none for the occupied calcium binding loop. In contrast, ^13^CO dispersions for several residues of the unoccupied loop could mean transient binding calcium binding that remained undetected by the ITC experiments and the dynamics of CLD N-lobe^Ca^ may reflect necessary plasticity for activation of *pf*CDPK3. Since the dynamics seem to reflect a concerted two-state process it should be possible to explore the structure of the alternative conformation in detail [44].

In conclusion, our results show that the solution structures of the calcium bound forms of the two lobes are similar to the corresponding fragment of the crystal structure of CAD while the structure of CLD N-lobe^apo^ is typical of EF-hand domains in absence of calcium. Our failure to prepare calcium-free CLD C-lobe suggests that the activation of *pf*CDPK3 is due to the conformational switch resulting from the binding of a single calcium ion to CLD N-lobe. In contrast to CLD C-lobe, the N-lobe is highly dynamic on the μs–ms time-scale. For CLD N-lobe^apo^ the dynamics may reflect a folding-unfolding transition and for CLD N-lobe^Ca^ they could be required for activation. Because of the low affinity for calcium and the lack of cooperativity within CLD N-lobe it is likely that higher calcium levels are needed to activate *pf*CDPK3 than most other calcium sensors including other *pf*CDPKs. If CLD C-lobe indeed is calcium-bound at low calcium levels, an interpretation of our observation that isolated CLD C-lobe forms dimers is that it interacts with the junction region also in the inactive state.

## Supporting information

S1 Text Expression and purification of CLD N-lobe and CLD C-lobe. (DOCX)

S1 Fig Thermal denaturation of CLD C-lobe^Ca^ monitored by circular dichroism spectroscopy. (DOCX)

S2 Fig ^1^HN *R*_1*ρ*_ dispersions for CLD N-lobe^apo^ at 500 MHz and 25°C. (DOCX)

S3 Fig ^15^N CPMG dispersions for CLD N-lobe^Ca^ at 600 MHz and 25°C. (DOCX)

S4 Fig ^15^N CPMG dispersions for CLD C-lobe^Ca^ at 600 MHz and 25°C. (DOCX)

S1 Table Coordinates for the selected CS-Rosetta model of pfCDPK3 CLD N-lobe^apo^. (DOCX)

S2 Table Coordinates for the selected CS-Rosetta model of *pf*CDPK3 CLD N-lobe^Ca^. (DOCX)

S3 Table Coordinates for the selected CS-Rosetta model of *pf*CDPK3 CLD C-lobe^Ca^. (DOCX)
