# Supplementary material for: Biophysical characterization of the calmodulin-like domain of Plasmodium falciparum calcium dependent protein kinase 3
Source: PLoS One. 2017 Jul 26;12(7):e0181721. doi: 10.1371/journal.pone.0181721 (PMC5528832; doi:10.1371/journal.pone.0181721)
Supplement: S1 Text — (DOCX) [file pone.0181721.s001.docx]

**S1 Text.** **Expression and purification of CLD N-lobe and CLD C-lobe.**

A gene encoding *pf*CDPK3 was purchased from GenScript. The fragments for CLD N-lobe (residues 416-484) and CLD C-lobe (residues 487-562) were subcloned into a pNIC28-Bsa4 vector that includes an N-terminal 6×His tag followed by a tobacco etch virus (TEV) protease recognition site.

The constructs were transformed into BL21(DE3) cells and expressed in M9 medium supplemented with 1 mM MgSO_4_, 0.1 mM CaCl_2_, MEM vitamins (Thermo Scientific), 0.5 g/l NH_4_Cl, 2 g/l glucose and 50 mg/l kanamycin. The culture was incubated at 37°C until OD_600_ reached 0.8. Expression was induced by addition of 0.5 mM IPTG and performed overnight at 16°C. The cells were harvested by centrifugation at 3000×g for 20 min at 4°C and the cell pellet was resuspended in 30 ml of 20 mM Tris pH 7.1, 150 mM NaCl, 10 mM CaCl_2_ and 5 mM imidazole (lysis buffer). Since CLD N-lobe is expressed in the soluble fraction and CLD C-lobe is found in inclusion bodies, the two domains had to be purified according to separate protocols.

For purification of CLD N-lobe, the bacteria were lysed by sonication on ice for 180 s and the lysate was centrifuged at 20000×g for 30 min at 4°C. The cleared lysate was applied to Ni-NTA superflow beads (GE Healthcare). The beads were washed with 20 mM Tris pH 7.1, 150 mM NaCl, 10 mM CaCl_2_ and 20 mM imidazole and the protein was eluted with 20 mM Tris pH 7.1, 150 mM NaCl, 10 mM CaCl_2_ and 250 mM imidazole. The His tag was cleaved using TEV protease during dialysis at room temperature overnight against 20 mM Tris pH 7.1, 150 mM NaCl, 10 mM CaCl_2_ and 5 mM imidazole. The His-tag and TEV protease were removed by Ni^2+^ affinity chromatography. The protein was further purified by size exclusion chromatography using a Sephadex 75 column (GE Healthcare) and concentrated using an Amicon® stirred cell (Merck Millipore). CLD N-lobe^apo^ was prepared by dialysis against 20 mM Tris pH 7.1, 150 mM NaCl, 2 mM EDTA and 5% glycerol, followed by two additional dialysis steps against 20 mM Tris pH 7.1, 150 mM NaCl.

For purification of CLD C-lobe, the bacteria were sonicated on ice for 180 s and cell debris and inclusion bodies were pelleted by centrifugation at 20000×g for 30 min at 4°C. The inclusion bodies were washed twice by resuspension in 30 ml lysis buffer followed by sonication and centrifugation as before and then solubilized in 8 M urea, 20 mM Tris pH 8.0, 150 mM NaCl and sonicated once more. The unfolded protein was applied to Ni-NTA superflow beads (GE Healthcare) and washed with solubilization buffer at pH 8.0 and pH 6.9 followed by elution with 8 M urea, 20 mM Tris pH 4.5, 150 mM NaCl. CLD C-lobe was then refolded by repeated dialysis against 20 mM Tris pH 7.1, 150 mM NaCl, 10 mM CaCl_2_ and 5 mM imidazole. Digestion with TEV protease and further purification was done as for CLD N-lobe.

Intact CLD was expressed and purified in the same was as CLD N-lobe.
