# Supplementary material for: Biophysical characterization of the calmodulin-like domain of Plasmodium falciparum calcium dependent protein kinase 3
Source: PLoS One. 2017 Jul 26;12(7):e0181721. doi: 10.1371/journal.pone.0181721 (PMC5528832; doi:10.1371/journal.pone.0181721)
Supplement: S1 Fig — (DOCX) [file pone.0181721.s002.docx]

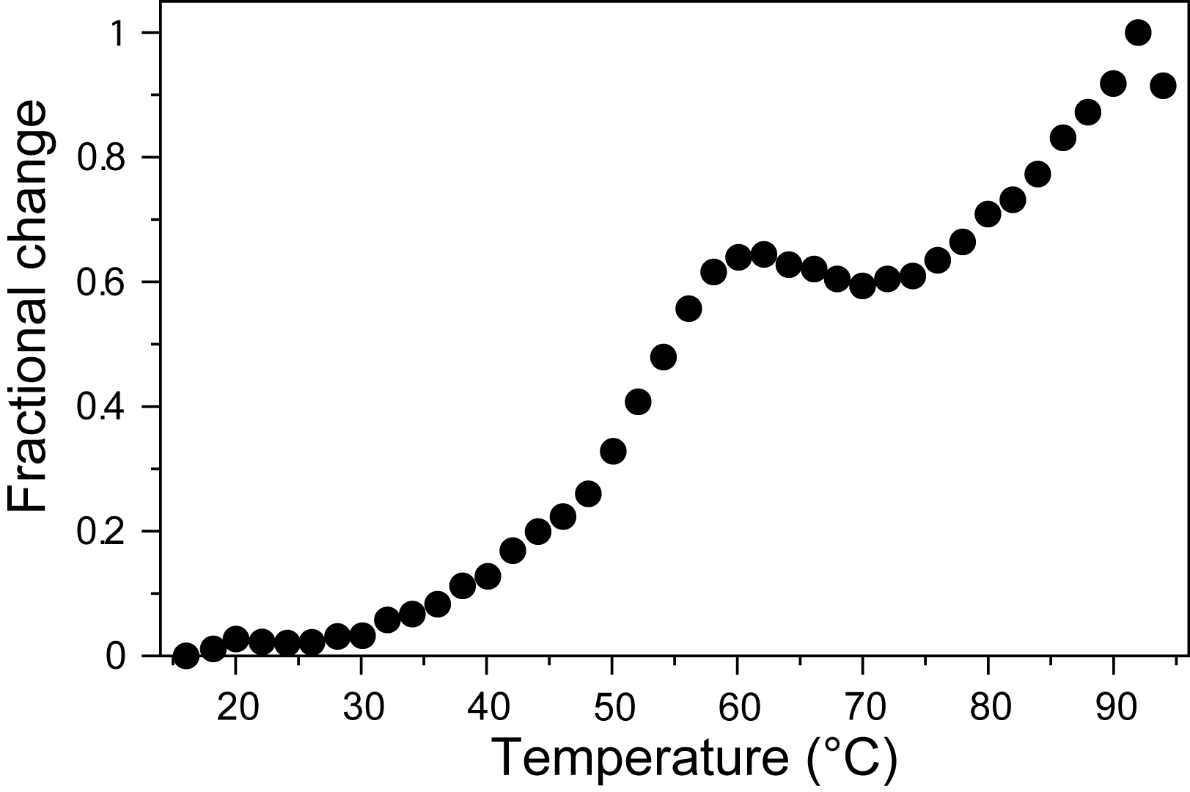


**S1 Fig. Thermal denaturation of CLD C-lobe^Ca^ monitored by circular dichroism spectroscopy.** The data was recorded at 222 nm using a ChiraScan spectrometer (Applied Photophysics Ltd). Sample conditions were 2 - 4 μM protein in 4 μM Tris pH 7.1, 0.3 mM NaCl and 20 μM CaCl_2_. The temperature was raised from 16°C to 94°C in 1°C increments and at each temperature the sample was equilibrated for one minute before measurements. The measurements were performed in triplicate. A representative profile is shown.
