# Supplementary material for: Biophysical characterization of the calmodulin-like domain of Plasmodium falciparum calcium dependent protein kinase 3
Source: PLoS One. 2017 Jul 26;12(7):e0181721. doi: 10.1371/journal.pone.0181721 (PMC5528832; doi:10.1371/journal.pone.0181721)
Supplement: S2 Fig — (DOCX) [file pone.0181721.s003.docx]

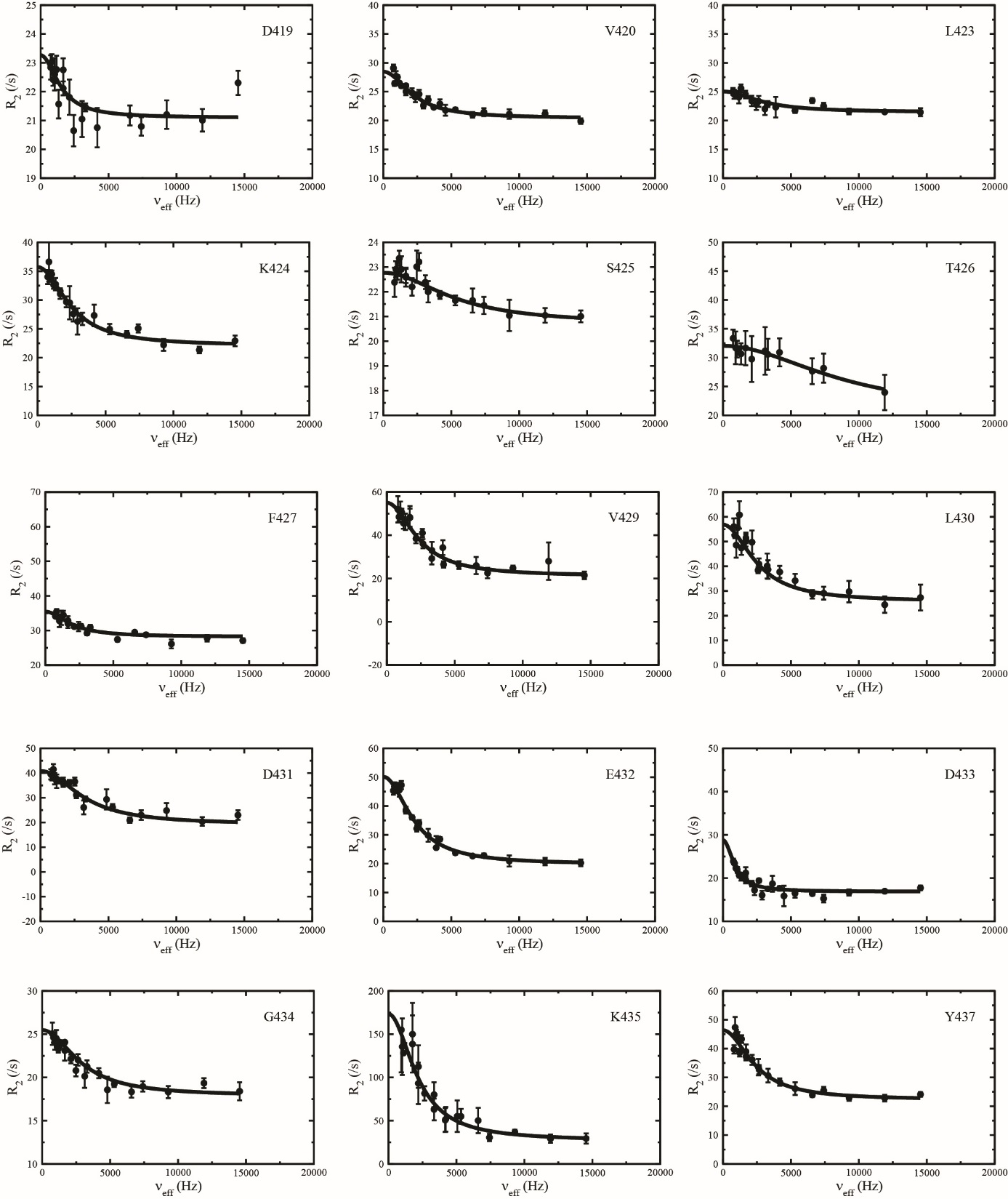

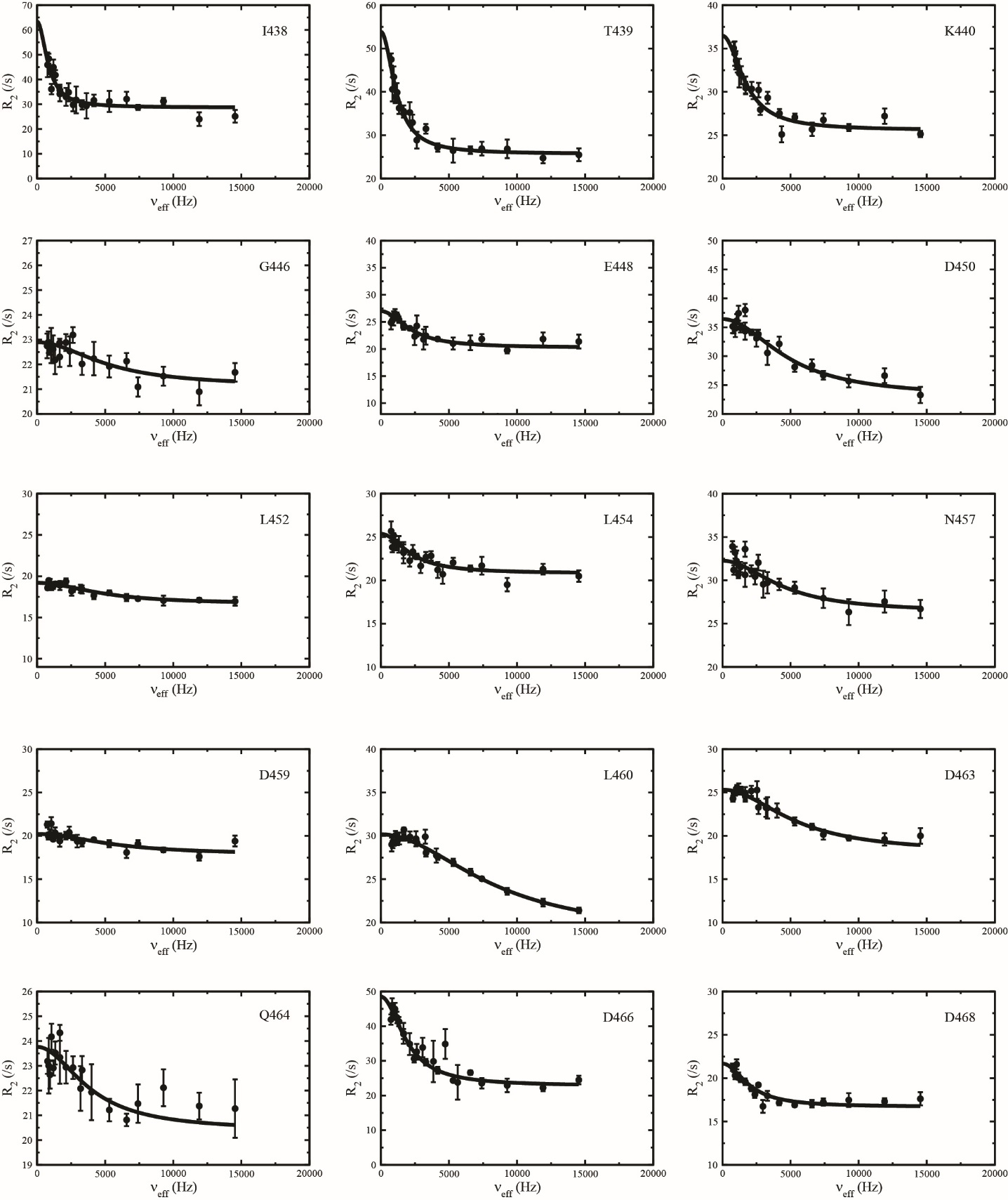

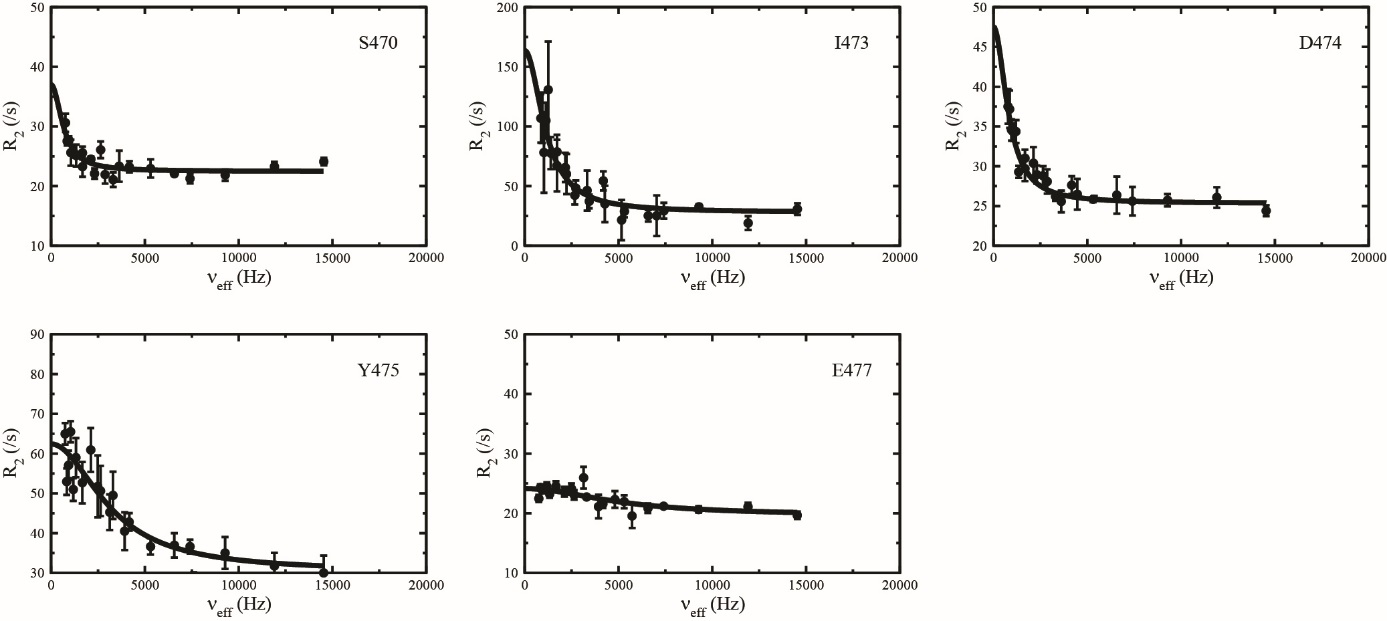


**S2 Fig.** **^1^HN *R*_1_*_ρ_* dispersions for CLD N-lobe^apo^ at 500 MHz and 25°C.** Filled circles represent experimental data and solid lines are the best fit to a two-state model on a per residue basis. Data for all residues with significant conformational dynamics (p<0.01) are shown.
