# Supplementary material for: Biophysical characterization of the calmodulin-like domain of Plasmodium falciparum calcium dependent protein kinase 3
Source: PLoS One. 2017 Jul 26;12(7):e0181721. doi: 10.1371/journal.pone.0181721 (PMC5528832; doi:10.1371/journal.pone.0181721)
Supplement: S4 Fig — (DOCX) [file pone.0181721.s005.docx]

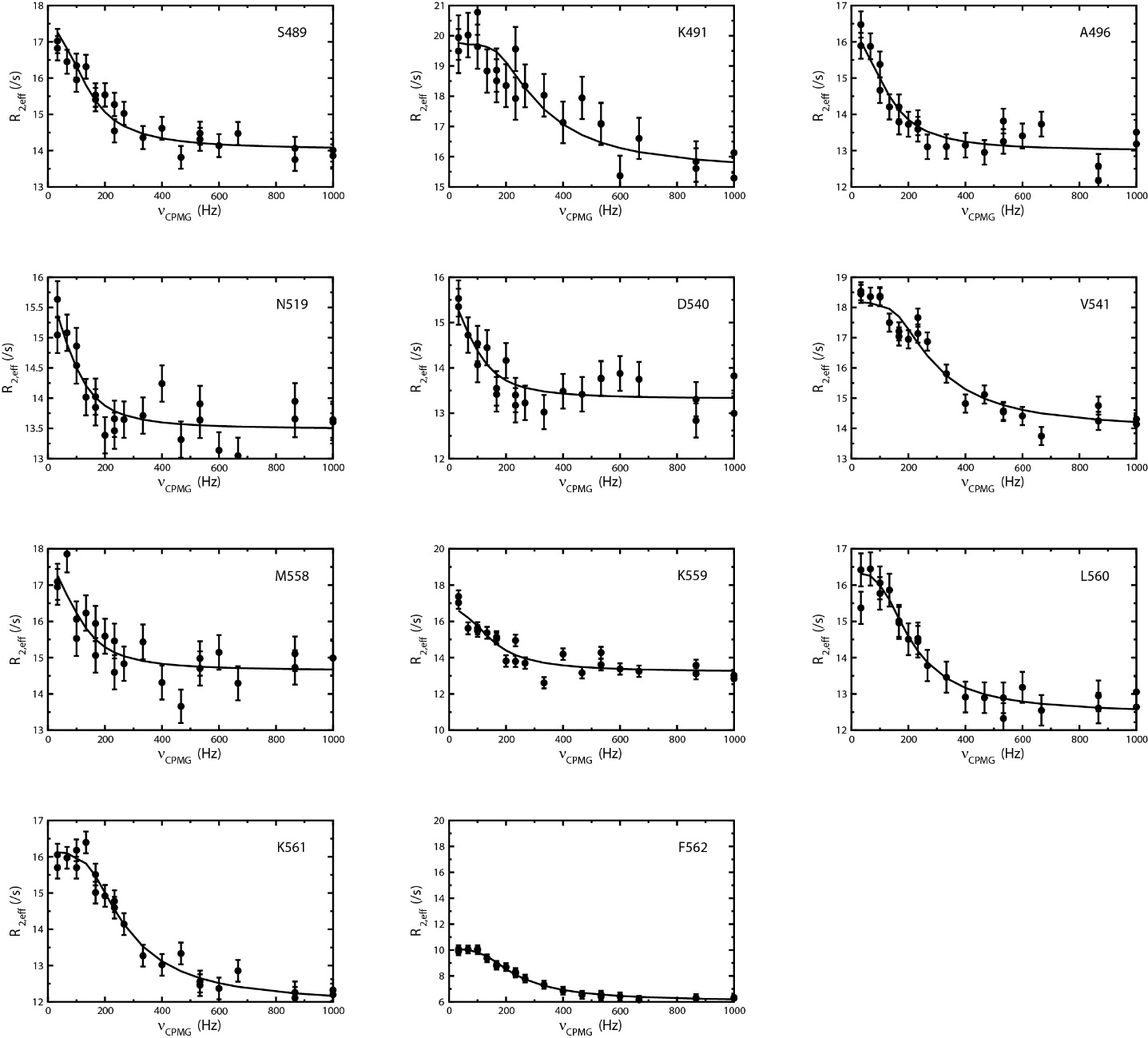


**S4 Fig.** ^15^N CPMG dispersions for CLD C-lobe^Ca^ at 600 MHz and 25°C. Filled circles represent experimental data and solid lines are the best fit to a global two-state model. Data for all residues with significant conformational dynamics (p<0.01) are shown.
