# Supplementary material for: Biophysical characterization of the calmodulin-like domain of Plasmodium falciparum calcium dependent protein kinase 3
Source: PLoS One. 2017 Jul 26;12(7):e0181721. doi: 10.1371/journal.pone.0181721 (PMC5528832; doi:10.1371/journal.pone.0181721)
Supplement: S1 Table — (DOCX) [file pone.0181721.s006.docx]

**S1 Table. Coordinates for the selected CS-Rosetta model of *pf*CDPK3 CLD N-lobe^apo^.**

ATOM 1 N GLY A 1 0.000 0.000 0.000 1.00 0.00 N

ATOM 2 CA GLY A 1 1.458 0.000 0.000 1.00 0.00 C

ATOM 3 C GLY A 1 2.009 1.420 0.000 1.00 0.00 C

ATOM 4 O GLY A 1 2.116 2.055 -1.049 1.00 0.00 O

ATOM 5 1H GLY A 1 -0.334 -0.943 -0.000 1.00 0.00 H

ATOM 6 2H GLY A 1 -0.334 0.471 0.816 1.00 0.00 H

ATOM 7 3H GLY A 1 -0.334 0.471 -0.816 1.00 0.00 H

ATOM 8 1HA GLY A 1 1.822 -0.535 0.877 1.00 0.00 H

ATOM 9 2HA GLY A 1 1.822 -0.535 -0.876 1.00 0.00 H

ATOM 10 N ASN A 2 2.358 1.914 1.183 1.00 0.00 N

ATOM 11 CA ASN A 2 2.936 3.246 1.317 1.00 0.00 C

ATOM 12 C ASN A 2 4.440 3.220 1.079 1.00 0.00 C

ATOM 13 O ASN A 2 5.009 2.177 0.755 1.00 0.00 O

ATOM 14 CB ASN A 2 2.621 3.830 2.682 1.00 0.00 C

ATOM 15 CG ASN A 2 3.311 3.095 3.798 1.00 0.00 C

ATOM 16 OD1 ASN A 2 4.385 2.514 3.605 1.00 0.00 O

ATOM 17 ND2 ASN A 2 2.714 3.110 4.963 1.00 0.00 N

ATOM 18 H ASN A 2 2.219 1.352 2.011 1.00 0.00 H

ATOM 19 HA ASN A 2 2.499 3.891 0.553 1.00 0.00 H

ATOM 20 1HB ASN A 2 2.925 4.877 2.709 1.00 0.00 H

ATOM 21 2HB ASN A 2 1.545 3.797 2.852 1.00 0.00 H

ATOM 22 1HD2 ASN A 2 3.128 2.637 5.742 1.00 0.00 H

ATOM 23 2HD2 ASN A 2 1.846 3.592 5.074 1.00 0.00 H

ATOM 24 N ASP A 3 5.080 4.373 1.243 1.00 0.00 N

ATOM 25 CA ASP A 3 6.518 4.488 1.028 1.00 0.00 C

ATOM 26 C ASP A 3 7.253 4.730 2.340 1.00 0.00 C

ATOM 27 O ASP A 3 8.334 5.319 2.358 1.00 0.00 O

ATOM 28 CB ASP A 3 6.824 5.623 0.048 1.00 0.00 C

ATOM 29 CG ASP A 3 6.322 5.340 -1.362 1.00 0.00 C

ATOM 30 OD1 ASP A 3 6.467 4.228 -1.811 1.00 0.00 O

ATOM 31 OD2 ASP A 3 5.797 6.238 -1.975 1.00 0.00 O

ATOM 32 H ASP A 3 4.557 5.190 1.523 1.00 0.00 H

ATOM 33 HA ASP A 3 6.880 3.552 0.602 1.00 0.00 H

ATOM 34 1HB ASP A 3 6.365 6.545 0.403 1.00 0.00 H

ATOM 35 2HB ASP A 3 7.901 5.788 0.008 1.00 0.00 H

ATOM 36 N TYR A 4 6.660 4.274 3.438 1.00 0.00 N

ATOM 37 CA TYR A 4 7.182 4.562 4.768 1.00 0.00 C

ATOM 38 C TYR A 4 7.340 3.288 5.587 1.00 0.00 C

ATOM 39 O TYR A 4 6.563 2.344 5.440 1.00 0.00 O

ATOM 40 CB TYR A 4 6.272 5.553 5.498 1.00 0.00 C

ATOM 41 CG TYR A 4 6.038 6.840 4.739 1.00 0.00 C

ATOM 42 CD1 TYR A 4 4.974 6.940 3.855 1.00 0.00 C

ATOM 43 CD2 TYR A 4 6.887 7.920 4.927 1.00 0.00 C

ATOM 44 CE1 TYR A 4 4.760 8.116 3.161 1.00 0.00 C

ATOM 45 CE2 TYR A 4 6.673 9.096 4.234 1.00 0.00 C

ATOM 46 CZ TYR A 4 5.615 9.196 3.355 1.00 0.00 C

ATOM 47 OH TYR A 4 5.402 10.367 2.664 1.00 0.00 O

ATOM 48 H TYR A 4 5.825 3.712 3.349 1.00 0.00 H

ATOM 49 HA TYR A 4 8.171 5.011 4.662 1.00 0.00 H

ATOM 50 1HB TYR A 4 5.303 5.087 5.685 1.00 0.00 H

ATOM 51 2HB TYR A 4 6.707 5.802 6.465 1.00 0.00 H

ATOM 52 HD1 TYR A 4 4.306 6.091 3.706 1.00 0.00 H

ATOM 53 HD2 TYR A 4 7.724 7.841 5.622 1.00 0.00 H

ATOM 54 HE1 TYR A 4 3.924 8.195 2.467 1.00 0.00 H

ATOM 55 HE2 TYR A 4 7.341 9.945 4.382 1.00 0.00 H

ATOM 56 HH TYR A 4 5.982 11.050 3.009 1.00 0.00 H

ATOM 57 N ASP A 5 8.349 3.266 6.450 1.00 0.00 N

ATOM 58 CA ASP A 5 8.636 2.090 7.264 1.00 0.00 C

ATOM 59 C ASP A 5 7.831 2.106 8.557 1.00 0.00 C

ATOM 60 O ASP A 5 8.165 2.825 9.499 1.00 0.00 O

ATOM 61 CB ASP A 5 10.130 2.013 7.587 1.00 0.00 C

ATOM 62 CG ASP A 5 10.505 0.756 8.360 1.00 0.00 C

ATOM 63 OD1 ASP A 5 9.686 0.274 9.105 1.00 0.00 O

ATOM 64 OD2 ASP A 5 11.607 0.290 8.197 1.00 0.00 O

ATOM 65 H ASP A 5 8.933 4.084 6.545 1.00 0.00 H

ATOM 66 HA ASP A 5 8.356 1.201 6.699 1.00 0.00 H

ATOM 67 1HB ASP A 5 10.705 2.038 6.661 1.00 0.00 H

ATOM 68 2HB ASP A 5 10.421 2.884 8.176 1.00 0.00 H

ATOM 69 N VAL A 6 6.769 1.309 8.596 1.00 0.00 N

ATOM 70 CA VAL A 6 5.827 1.341 9.709 1.00 0.00 C

ATOM 71 C VAL A 6 6.474 0.839 10.993 1.00 0.00 C

ATOM 72 O VAL A 6 6.171 1.324 12.083 1.00 0.00 O

ATOM 73 CB VAL A 6 4.591 0.480 9.388 1.00 0.00 C

ATOM 74 CG1 VAL A 6 3.672 0.397 10.598 1.00 0.00 C

ATOM 75 CG2 VAL A 6 3.855 1.057 8.189 1.00 0.00 C

ATOM 76 H VAL A 6 6.609 0.664 7.836 1.00 0.00 H

ATOM 77 HA VAL A 6 5.505 2.373 9.860 1.00 0.00 H

ATOM 78 HB VAL A 6 4.917 -0.536 9.162 1.00 0.00 H

ATOM 79 1HG1 VAL A 6 2.804 -0.216 10.353 1.00 0.00 H

ATOM 80 2HG1 VAL A 6 4.209 -0.052 11.433 1.00 0.00 H

ATOM 81 3HG1 VAL A 6 3.341 1.398 10.873 1.00 0.00 H

ATOM 82 1HG2 VAL A 6 2.983 0.442 7.968 1.00 0.00 H

ATOM 83 2HG2 VAL A 6 3.534 2.074 8.414 1.00 0.00 H

ATOM 84 3HG2 VAL A 6 4.519 1.068 7.325 1.00 0.00 H

ATOM 85 N GLU A 7 7.366 -0.136 10.858 1.00 0.00 N

ATOM 86 CA GLU A 7 8.006 -0.755 12.012 1.00 0.00 C

ATOM 87 C GLU A 7 8.914 0.232 12.734 1.00 0.00 C

ATOM 88 O GLU A 7 9.032 0.200 13.959 1.00 0.00 O

ATOM 89 CB GLU A 7 8.811 -1.983 11.582 1.00 0.00 C

ATOM 90 CG GLU A 7 7.964 -3.148 11.088 1.00 0.00 C

ATOM 91 CD GLU A 7 7.008 -3.659 12.129 1.00 0.00 C

ATOM 92 OE1 GLU A 7 7.436 -3.913 13.229 1.00 0.00 O

ATOM 93 OE2 GLU A 7 5.846 -3.795 11.824 1.00 0.00 O

ATOM 94 H GLU A 7 7.608 -0.456 9.931 1.00 0.00 H

ATOM 95 HA GLU A 7 7.229 -1.073 12.709 1.00 0.00 H

ATOM 96 1HB GLU A 7 9.497 -1.706 10.782 1.00 0.00 H

ATOM 97 2HB GLU A 7 9.411 -2.338 12.420 1.00 0.00 H

ATOM 98 1HG GLU A 7 7.396 -2.827 10.216 1.00 0.00 H

ATOM 99 2HG GLU A 7 8.624 -3.958 10.780 1.00 0.00 H

ATOM 100 N LYS A 8 9.556 1.107 11.967 1.00 0.00 N

ATOM 101 CA LYS A 8 10.361 2.180 12.539 1.00 0.00 C

ATOM 102 C LYS A 8 9.486 3.226 13.216 1.00 0.00 C

ATOM 103 O LYS A 8 9.864 3.798 14.239 1.00 0.00 O

ATOM 104 CB LYS A 8 11.223 2.836 11.458 1.00 0.00 C

ATOM 105 CG LYS A 8 12.400 1.989 10.992 1.00 0.00 C

ATOM 106 CD LYS A 8 13.202 2.703 9.915 1.00 0.00 C

ATOM 107 CE LYS A 8 14.365 1.848 9.431 1.00 0.00 C

ATOM 108 NZ LYS A 8 15.123 2.510 8.335 1.00 0.00 N

ATOM 109 H LYS A 8 9.483 1.028 10.963 1.00 0.00 H

ATOM 110 HA LYS A 8 11.018 1.753 13.297 1.00 0.00 H

ATOM 111 1HB LYS A 8 10.607 3.060 10.588 1.00 0.00 H

ATOM 112 2HB LYS A 8 11.618 3.781 11.832 1.00 0.00 H

ATOM 113 1HG LYS A 8 13.053 1.776 11.839 1.00 0.00 H

ATOM 114 2HG LYS A 8 12.032 1.044 10.593 1.00 0.00 H

ATOM 115 1HD LYS A 8 12.553 2.932 9.069 1.00 0.00 H

ATOM 116 2HD LYS A 8 13.594 3.639 10.314 1.00 0.00 H

ATOM 117 1HE LYS A 8 15.043 1.655 10.261 1.00 0.00 H

ATOM 118 2HE LYS A 8 13.987 0.892 9.069 1.00 0.00 H

ATOM 119 1HZ LYS A 8 15.883 1.912 8.042 1.00 0.00 H

ATOM 120 2HZ LYS A 8 14.507 2.677 7.552 1.00 0.00 H

ATOM 121 3HZ LYS A 8 15.495 3.389 8.664 1.00 0.00 H

ATOM 122 N LEU A 9 8.313 3.472 12.641 1.00 0.00 N

ATOM 123 CA LEU A 9 7.331 4.358 13.253 1.00 0.00 C

ATOM 124 C LEU A 9 6.837 3.800 14.581 1.00 0.00 C

ATOM 125 O LEU A 9 6.609 4.547 15.533 1.00 0.00 O

ATOM 126 CB LEU A 9 6.143 4.566 12.305 1.00 0.00 C

ATOM 127 CG LEU A 9 6.451 5.326 11.008 1.00 0.00 C

ATOM 128 CD1 LEU A 9 5.210 5.351 10.127 1.00 0.00 C

ATOM 129 CD2 LEU A 9 6.912 6.736 11.343 1.00 0.00 C

ATOM 130 H LEU A 9 8.098 3.033 11.757 1.00 0.00 H

ATOM 131 HA LEU A 9 7.803 5.322 13.439 1.00 0.00 H

ATOM 132 1HB LEU A 9 5.742 3.592 12.031 1.00 0.00 H

ATOM 133 2HB LEU A 9 5.367 5.118 12.836 1.00 0.00 H

ATOM 134 HG LEU A 9 7.238 4.806 10.461 1.00 0.00 H

ATOM 135 1HD1 LEU A 9 5.429 5.890 9.205 1.00 0.00 H

ATOM 136 2HD1 LEU A 9 4.913 4.329 9.887 1.00 0.00 H

ATOM 137 3HD1 LEU A 9 4.398 5.850 10.655 1.00 0.00 H

ATOM 138 1HD2 LEU A 9 7.132 7.276 10.421 1.00 0.00 H

ATOM 139 2HD2 LEU A 9 6.125 7.257 11.889 1.00 0.00 H

ATOM 140 3HD2 LEU A 9 7.811 6.689 11.959 1.00 0.00 H

ATOM 141 N LYS A 10 6.674 2.483 14.640 1.00 0.00 N

ATOM 142 CA LYS A 10 6.340 1.807 15.888 1.00 0.00 C

ATOM 143 C LYS A 10 7.406 2.052 16.949 1.00 0.00 C

ATOM 144 O LYS A 10 7.096 2.419 18.082 1.00 0.00 O

ATOM 145 CB LYS A 10 6.165 0.306 15.655 1.00 0.00 C

ATOM 146 CG LYS A 10 4.912 -0.067 14.874 1.00 0.00 C

ATOM 147 CD LYS A 10 4.847 -1.565 14.617 1.00 0.00 C

ATOM 148 CE LYS A 10 3.600 -1.939 13.829 1.00 0.00 C

ATOM 149 NZ LYS A 10 3.572 -3.387 13.486 1.00 0.00 N

ATOM 150 H LYS A 10 6.783 1.936 13.798 1.00 0.00 H

ATOM 151 HA LYS A 10 5.400 2.214 16.261 1.00 0.00 H

ATOM 152 1HB LYS A 10 7.027 -0.080 15.109 1.00 0.00 H

ATOM 153 2HB LYS A 10 6.128 -0.210 16.614 1.00 0.00 H

ATOM 154 1HG LYS A 10 4.029 0.235 15.437 1.00 0.00 H

ATOM 155 2HG LYS A 10 4.909 0.457 13.919 1.00 0.00 H

ATOM 156 1HD LYS A 10 5.728 -1.877 14.056 1.00 0.00 H

ATOM 157 2HD LYS A 10 4.836 -2.097 15.568 1.00 0.00 H

ATOM 158 1HE LYS A 10 2.714 -1.700 14.416 1.00 0.00 H

ATOM 159 2HE LYS A 10 3.565 -1.359 12.907 1.00 0.00 H

ATOM 160 1HZ LYS A 10 2.731 -3.593 12.964 1.00 0.00 H

ATOM 161 2HZ LYS A 10 4.380 -3.616 12.925 1.00 0.00 H

ATOM 162 3HZ LYS A 10 3.584 -3.935 14.334 1.00 0.00 H

ATOM 163 N SER A 11 8.664 1.846 16.574 1.00 0.00 N

ATOM 164 CA SER A 11 9.773 1.971 17.512 1.00 0.00 C

ATOM 165 C SER A 11 9.834 3.371 18.110 1.00 0.00 C

ATOM 166 O SER A 11 9.846 3.536 19.330 1.00 0.00 O

ATOM 167 CB SER A 11 11.083 1.651 16.819 1.00 0.00 C

ATOM 168 OG SER A 11 12.163 1.778 17.702 1.00 0.00 O

ATOM 169 H SER A 11 8.857 1.596 15.615 1.00 0.00 H

ATOM 170 HA SER A 11 9.622 1.259 18.325 1.00 0.00 H

ATOM 171 1HB SER A 11 11.048 0.635 16.426 1.00 0.00 H

ATOM 172 2HB SER A 11 11.220 2.323 15.973 1.00 0.00 H

ATOM 173 HG SER A 11 12.608 2.594 17.460 1.00 0.00 H

ATOM 174 N THR A 12 9.871 4.378 17.243 1.00 0.00 N

ATOM 175 CA THR A 12 10.020 5.760 17.681 1.00 0.00 C

ATOM 176 C THR A 12 8.915 6.152 18.654 1.00 0.00 C

ATOM 177 O THR A 12 9.173 6.781 19.680 1.00 0.00 O

ATOM 178 CB THR A 12 10.018 6.725 16.481 1.00 0.00 C

ATOM 179 OG1 THR A 12 11.141 6.443 15.636 1.00 0.00 O

ATOM 180 CG2 THR A 12 10.095 8.168 16.956 1.00 0.00 C

ATOM 181 H THR A 12 9.794 4.180 16.256 1.00 0.00 H

ATOM 182 HA THR A 12 10.974 5.859 18.199 1.00 0.00 H

ATOM 183 HB THR A 12 9.104 6.587 15.904 1.00 0.00 H

ATOM 184 HG1 THR A 12 11.854 7.055 15.837 1.00 0.00 H

ATOM 185 1HG2 THR A 12 10.093 8.835 16.094 1.00 0.00 H

ATOM 186 2HG2 THR A 12 9.236 8.389 17.589 1.00 0.00 H

ATOM 187 3HG2 THR A 12 11.013 8.315 17.525 1.00 0.00 H

ATOM 188 N PHE A 13 7.684 5.776 18.326 1.00 0.00 N

ATOM 189 CA PHE A 13 6.541 6.061 19.185 1.00 0.00 C

ATOM 190 C PHE A 13 6.758 5.512 20.590 1.00 0.00 C

ATOM 191 O PHE A 13 6.550 6.213 21.580 1.00 0.00 O

ATOM 192 CB PHE A 13 5.264 5.465 18.591 1.00 0.00 C

ATOM 193 CG PHE A 13 4.018 5.818 19.354 1.00 0.00 C

ATOM 194 CD1 PHE A 13 3.036 6.609 18.778 1.00 0.00 C

ATOM 195 CD2 PHE A 13 3.827 5.360 20.648 1.00 0.00 C

ATOM 196 CE1 PHE A 13 1.890 6.935 19.478 1.00 0.00 C

ATOM 197 CE2 PHE A 13 2.682 5.682 21.351 1.00 0.00 C

ATOM 198 CZ PHE A 13 1.713 6.471 20.765 1.00 0.00 C

ATOM 199 H PHE A 13 7.535 5.281 17.458 1.00 0.00 H

ATOM 200 HA PHE A 13 6.422 7.143 19.255 1.00 0.00 H

ATOM 201 1HB PHE A 13 5.144 5.811 17.565 1.00 0.00 H

ATOM 202 2HB PHE A 13 5.348 4.379 18.562 1.00 0.00 H

ATOM 203 HD1 PHE A 13 3.176 6.975 17.760 1.00 0.00 H

ATOM 204 HD2 PHE A 13 4.593 4.737 21.111 1.00 0.00 H

ATOM 205 HE1 PHE A 13 1.126 7.558 19.014 1.00 0.00 H

ATOM 206 HE2 PHE A 13 2.544 5.315 22.368 1.00 0.00 H

ATOM 207 HZ PHE A 13 0.811 6.728 21.318 1.00 0.00 H

ATOM 208 N LEU A 14 7.177 4.254 20.669 1.00 0.00 N

ATOM 209 CA LEU A 14 7.360 3.587 21.953 1.00 0.00 C

ATOM 210 C LEU A 14 8.512 4.204 22.735 1.00 0.00 C

ATOM 211 O LEU A 14 8.471 4.279 23.963 1.00 0.00 O

ATOM 212 CB LEU A 14 7.623 2.091 21.740 1.00 0.00 C

ATOM 213 CG LEU A 14 6.445 1.281 21.183 1.00 0.00 C

ATOM 214 CD1 LEU A 14 6.901 -0.141 20.886 1.00 0.00 C

ATOM 215 CD2 LEU A 14 5.302 1.290 22.188 1.00 0.00 C

ATOM 216 H LEU A 14 7.374 3.746 19.819 1.00 0.00 H

ATOM 217 HA LEU A 14 6.447 3.703 22.536 1.00 0.00 H

ATOM 218 1HB LEU A 14 8.457 1.979 21.049 1.00 0.00 H

ATOM 219 2HB LEU A 14 7.907 1.648 22.695 1.00 0.00 H

ATOM 220 HG LEU A 14 6.108 1.726 20.247 1.00 0.00 H

ATOM 221 1HD1 LEU A 14 6.064 -0.716 20.490 1.00 0.00 H

ATOM 222 2HD1 LEU A 14 7.705 -0.119 20.150 1.00 0.00 H

ATOM 223 3HD1 LEU A 14 7.260 -0.607 21.803 1.00 0.00 H

ATOM 224 1HD2 LEU A 14 4.465 0.715 21.792 1.00 0.00 H

ATOM 225 2HD2 LEU A 14 5.638 0.844 23.125 1.00 0.00 H

ATOM 226 3HD2 LEU A 14 4.985 2.317 22.369 1.00 0.00 H

ATOM 227 N VAL A 15 9.539 4.647 22.017 1.00 0.00 N

ATOM 228 CA VAL A 15 10.656 5.354 22.632 1.00 0.00 C

ATOM 229 C VAL A 15 10.210 6.691 23.210 1.00 0.00 C

ATOM 230 O VAL A 15 10.598 7.058 24.319 1.00 0.00 O

ATOM 231 CB VAL A 15 11.772 5.591 21.598 1.00 0.00 C

ATOM 232 CG1 VAL A 15 12.826 6.534 22.159 1.00 0.00 C

ATOM 233 CG2 VAL A 15 12.395 4.263 21.194 1.00 0.00 C

ATOM 234 H VAL A 15 9.545 4.489 21.019 1.00 0.00 H

ATOM 235 HA VAL A 15 11.052 4.738 23.440 1.00 0.00 H

ATOM 236 HB VAL A 15 11.344 6.074 20.719 1.00 0.00 H

ATOM 237 1HG1 VAL A 15 13.608 6.690 21.414 1.00 0.00 H

ATOM 238 2HG1 VAL A 15 12.365 7.489 22.407 1.00 0.00 H

ATOM 239 3HG1 VAL A 15 13.264 6.097 23.056 1.00 0.00 H

ATOM 240 1HG2 VAL A 15 13.183 4.439 20.462 1.00 0.00 H

ATOM 241 2HG2 VAL A 15 12.818 3.778 22.074 1.00 0.00 H

ATOM 242 3HG2 VAL A 15 11.631 3.621 20.757 1.00 0.00 H

ATOM 243 N LEU A 16 9.394 7.415 22.452 1.00 0.00 N

ATOM 244 CA LEU A 16 8.855 8.690 22.908 1.00 0.00 C

ATOM 245 C LEU A 16 7.945 8.504 24.115 1.00 0.00 C

ATOM 246 O LEU A 16 7.913 9.342 25.016 1.00 0.00 O

ATOM 247 CB LEU A 16 8.078 9.373 21.775 1.00 0.00 C

ATOM 248 CG LEU A 16 8.925 9.882 20.601 1.00 0.00 C

ATOM 249 CD1 LEU A 16 8.008 10.356 19.481 1.00 0.00 C

ATOM 250 CD2 LEU A 16 9.830 11.008 21.079 1.00 0.00 C

ATOM 251 H LEU A 16 9.140 7.073 21.536 1.00 0.00 H

ATOM 252 HA LEU A 16 9.686 9.332 23.200 1.00 0.00 H

ATOM 253 1HB LEU A 16 7.352 8.667 21.377 1.00 0.00 H

ATOM 254 2HB LEU A 16 7.538 10.225 22.188 1.00 0.00 H

ATOM 255 HG LEU A 16 9.534 9.067 20.211 1.00 0.00 H

ATOM 256 1HD1 LEU A 16 8.609 10.717 18.647 1.00 0.00 H

ATOM 257 2HD1 LEU A 16 7.385 9.526 19.146 1.00 0.00 H

ATOM 258 3HD1 LEU A 16 7.373 11.162 19.847 1.00 0.00 H

ATOM 259 1HD2 LEU A 16 10.432 11.369 20.244 1.00 0.00 H

ATOM 260 2HD2 LEU A 16 9.222 11.824 21.468 1.00 0.00 H

ATOM 261 3HD2 LEU A 16 10.487 10.637 21.866 1.00 0.00 H

ATOM 262 N ASP A 17 7.205 7.400 24.127 1.00 0.00 N

ATOM 263 CA ASP A 17 6.275 7.113 25.212 1.00 0.00 C

ATOM 264 C ASP A 17 7.015 6.715 26.482 1.00 0.00 C

ATOM 265 O ASP A 17 7.085 5.535 26.827 1.00 0.00 O

ATOM 266 CB ASP A 17 5.306 5.999 24.808 1.00 0.00 C

ATOM 267 CG ASP A 17 4.189 5.788 25.821 1.00 0.00 C

ATOM 268 OD1 ASP A 17 4.125 6.535 26.769 1.00 0.00 O

ATOM 269 OD2 ASP A 17 3.412 4.882 25.638 1.00 0.00 O

ATOM 270 H ASP A 17 7.290 6.743 23.365 1.00 0.00 H

ATOM 271 HA ASP A 17 5.699 8.015 25.422 1.00 0.00 H

ATOM 272 1HB ASP A 17 4.860 6.237 23.842 1.00 0.00 H

ATOM 273 2HB ASP A 17 5.855 5.064 24.694 1.00 0.00 H

ATOM 274 N GLU A 18 7.567 7.705 27.175 1.00 0.00 N

ATOM 275 CA GLU A 18 8.264 7.465 28.433 1.00 0.00 C

ATOM 276 C GLU A 18 7.281 7.261 29.578 1.00 0.00 C

ATOM 277 O GLU A 18 7.618 6.662 30.599 1.00 0.00 O

ATOM 278 CB GLU A 18 9.202 8.631 28.754 1.00 0.00 C

ATOM 279 CG GLU A 18 10.377 8.775 27.797 1.00 0.00 C

ATOM 280 CD GLU A 18 11.277 9.927 28.145 1.00 0.00 C

ATOM 281 OE1 GLU A 18 10.930 10.685 29.019 1.00 0.00 O

ATOM 282 OE2 GLU A 18 12.314 10.050 27.536 1.00 0.00 O

ATOM 283 H GLU A 18 7.501 8.649 26.822 1.00 0.00 H

ATOM 284 HA GLU A 18 8.859 6.556 28.330 1.00 0.00 H

ATOM 285 1HB GLU A 18 8.640 9.565 28.737 1.00 0.00 H

ATOM 286 2HB GLU A 18 9.603 8.508 29.760 1.00 0.00 H

ATOM 287 1HG GLU A 18 10.960 7.855 27.814 1.00 0.00 H

ATOM 288 2HG GLU A 18 9.994 8.911 26.787 1.00 0.00 H

ATOM 289 N ASP A 19 6.063 7.763 29.402 1.00 0.00 N

ATOM 290 CA ASP A 19 5.025 7.628 30.417 1.00 0.00 C

ATOM 291 C ASP A 19 4.475 6.209 30.456 1.00 0.00 C

ATOM 292 O ASP A 19 4.125 5.697 31.520 1.00 0.00 O

ATOM 293 CB ASP A 19 3.886 8.617 30.154 1.00 0.00 C

ATOM 294 CG ASP A 19 4.294 10.066 30.383 1.00 0.00 C

ATOM 295 OD1 ASP A 19 5.307 10.286 31.005 1.00 0.00 O

ATOM 296 OD2 ASP A 19 3.590 10.938 29.935 1.00 0.00 O

ATOM 297 H ASP A 19 5.852 8.251 28.544 1.00 0.00 H

ATOM 298 HA ASP A 19 5.462 7.852 31.391 1.00 0.00 H

ATOM 299 1HB ASP A 19 3.542 8.510 29.125 1.00 0.00 H

ATOM 300 2HB ASP A 19 3.044 8.384 30.807 1.00 0.00 H

ATOM 301 N GLY A 20 4.399 5.576 29.290 1.00 0.00 N

ATOM 302 CA GLY A 20 3.884 4.216 29.188 1.00 0.00 C

ATOM 303 C GLY A 20 2.363 4.208 29.107 1.00 0.00 C

ATOM 304 O GLY A 20 1.705 3.348 29.693 1.00 0.00 O

ATOM 305 H GLY A 20 4.707 6.050 28.453 1.00 0.00 H

ATOM 306 1HA GLY A 20 4.303 3.734 28.305 1.00 0.00 H

ATOM 307 2HA GLY A 20 4.209 3.639 30.053 1.00 0.00 H

ATOM 308 N LYS A 21 1.810 5.169 28.376 1.00 0.00 N

ATOM 309 CA LYS A 21 0.363 5.313 28.269 1.00 0.00 C

ATOM 310 C LYS A 21 -0.132 4.896 26.890 1.00 0.00 C

ATOM 311 O LYS A 21 -1.284 4.493 26.729 1.00 0.00 O

ATOM 312 CB LYS A 21 -0.055 6.755 28.563 1.00 0.00 C

ATOM 313 CG LYS A 21 0.333 7.252 29.949 1.00 0.00 C

ATOM 314 CD LYS A 21 -0.351 6.440 31.038 1.00 0.00 C

ATOM 315 CE LYS A 21 -0.020 6.978 32.422 1.00 0.00 C

ATOM 316 NZ LYS A 21 -0.661 6.177 33.500 1.00 0.00 N

ATOM 317 H LYS A 21 2.407 5.817 27.881 1.00 0.00 H

ATOM 318 HA LYS A 21 -0.106 4.658 29.004 1.00 0.00 H

ATOM 319 1HB LYS A 21 0.399 7.422 27.829 1.00 0.00 H

ATOM 320 2HB LYS A 21 -1.137 6.848 28.464 1.00 0.00 H

ATOM 321 1HG LYS A 21 1.413 7.175 30.075 1.00 0.00 H

ATOM 322 2HG LYS A 21 0.047 8.298 30.054 1.00 0.00 H

ATOM 323 1HD LYS A 21 -1.432 6.474 30.894 1.00 0.00 H

ATOM 324 2HD LYS A 21 -0.027 5.401 30.976 1.00 0.00 H

ATOM 325 1HE LYS A 21 1.059 6.964 32.569 1.00 0.00 H

ATOM 326 2HE LYS A 21 -0.361 8.010 32.503 1.00 0.00 H

ATOM 327 1HZ LYS A 21 -0.418 6.566 34.400 1.00 0.00 H

ATOM 328 2HZ LYS A 21 -1.665 6.199 33.384 1.00 0.00 H

ATOM 329 3HZ LYS A 21 -0.338 5.222 33.448 1.00 0.00 H

ATOM 330 N GLY A 22 0.746 4.994 25.897 1.00 0.00 N

ATOM 331 CA GLY A 22 0.378 4.698 24.517 1.00 0.00 C

ATOM 332 C GLY A 22 -0.325 5.883 23.868 1.00 0.00 C

ATOM 333 O GLY A 22 -1.054 5.724 22.889 1.00 0.00 O

ATOM 334 H GLY A 22 1.692 5.281 26.103 1.00 0.00 H

ATOM 335 1HA GLY A 22 1.273 4.444 23.949 1.00 0.00 H

ATOM 336 2HA GLY A 22 -0.275 3.826 24.494 1.00 0.00 H

ATOM 337 N TYR A 23 -0.101 7.071 24.419 1.00 0.00 N

ATOM 338 CA TYR A 23 -0.718 8.285 23.899 1.00 0.00 C

ATOM 339 C TYR A 23 0.297 9.416 23.788 1.00 0.00 C

ATOM 340 O TYR A 23 0.850 9.866 24.791 1.00 0.00 O

ATOM 341 CB TYR A 23 -1.892 8.710 24.784 1.00 0.00 C

ATOM 342 CG TYR A 23 -3.115 7.832 24.640 1.00 0.00 C

ATOM 343 CD1 TYR A 23 -3.280 6.733 25.470 1.00 0.00 C

ATOM 344 CD2 TYR A 23 -4.070 8.126 23.680 1.00 0.00 C

ATOM 345 CE1 TYR A 23 -4.398 5.930 25.338 1.00 0.00 C

ATOM 346 CE2 TYR A 23 -5.187 7.324 23.548 1.00 0.00 C

ATOM 347 CZ TYR A 23 -5.352 6.230 24.373 1.00 0.00 C

ATOM 348 OH TYR A 23 -6.465 5.432 24.242 1.00 0.00 O

ATOM 349 H TYR A 23 0.513 7.135 25.218 1.00 0.00 H

ATOM 350 HA TYR A 23 -1.095 8.080 22.896 1.00 0.00 H

ATOM 351 1HB TYR A 23 -1.583 8.695 25.831 1.00 0.00 H

ATOM 352 2HB TYR A 23 -2.177 9.734 24.543 1.00 0.00 H

ATOM 353 HD1 TYR A 23 -2.529 6.501 26.225 1.00 0.00 H

ATOM 354 HD2 TYR A 23 -3.940 8.990 23.027 1.00 0.00 H

ATOM 355 HE1 TYR A 23 -4.528 5.067 25.990 1.00 0.00 H

ATOM 356 HE2 TYR A 23 -5.938 7.555 22.792 1.00 0.00 H

ATOM 357 HH TYR A 23 -6.327 4.807 23.526 1.00 0.00 H

ATOM 358 N ILE A 24 0.537 9.871 22.563 1.00 0.00 N

ATOM 359 CA ILE A 24 1.508 10.930 22.316 1.00 0.00 C

ATOM 360 C ILE A 24 0.922 12.016 21.423 1.00 0.00 C

ATOM 361 O ILE A 24 0.185 11.728 20.480 1.00 0.00 O

ATOM 362 CB ILE A 24 2.784 10.364 21.666 1.00 0.00 C

ATOM 363 CG1 ILE A 24 3.416 9.301 22.569 1.00 0.00 C

ATOM 364 CG2 ILE A 24 3.775 11.481 21.380 1.00 0.00 C

ATOM 365 CD1 ILE A 24 4.614 8.613 21.955 1.00 0.00 C

ATOM 366 H ILE A 24 0.034 9.472 21.783 1.00 0.00 H

ATOM 367 HA ILE A 24 1.777 11.381 23.270 1.00 0.00 H

ATOM 368 HB ILE A 24 2.527 9.870 20.730 1.00 0.00 H

ATOM 369 1HG1 ILE A 24 3.729 9.759 23.507 1.00 0.00 H

ATOM 370 2HG1 ILE A 24 2.673 8.540 22.810 1.00 0.00 H

ATOM 371 1HG2 ILE A 24 4.672 11.064 20.922 1.00 0.00 H

ATOM 372 2HG2 ILE A 24 3.323 12.203 20.701 1.00 0.00 H

ATOM 373 3HG2 ILE A 24 4.042 11.979 22.313 1.00 0.00 H

ATOM 374 1HD1 ILE A 24 5.006 7.873 22.654 1.00 0.00 H

ATOM 375 2HD1 ILE A 24 4.315 8.116 21.032 1.00 0.00 H

ATOM 376 3HD1 ILE A 24 5.385 9.351 21.738 1.00 0.00 H

ATOM 377 N THR A 25 1.254 13.267 21.727 1.00 0.00 N

ATOM 378 CA THR A 25 0.737 14.401 20.971 1.00 0.00 C

ATOM 379 C THR A 25 1.354 14.465 19.580 1.00 0.00 C

ATOM 380 O THR A 25 2.450 13.952 19.353 1.00 0.00 O

ATOM 381 CB THR A 25 0.996 15.726 21.713 1.00 0.00 C

ATOM 382 OG1 THR A 25 2.409 15.940 21.833 1.00 0.00 O

ATOM 383 CG2 THR A 25 0.373 15.691 23.100 1.00 0.00 C

ATOM 384 H THR A 25 1.881 13.435 22.500 1.00 0.00 H

ATOM 385 HA THR A 25 -0.340 14.278 20.856 1.00 0.00 H

ATOM 386 HB THR A 25 0.564 16.551 21.147 1.00 0.00 H

ATOM 387 HG1 THR A 25 2.771 15.327 22.477 1.00 0.00 H

ATOM 388 1HG2 THR A 25 0.566 16.635 23.609 1.00 0.00 H

ATOM 389 2HG2 THR A 25 -0.703 15.540 23.012 1.00 0.00 H

ATOM 390 3HG2 THR A 25 0.808 14.874 23.674 1.00 0.00 H

ATOM 391 N LYS A 26 0.644 15.096 18.651 1.00 0.00 N

ATOM 392 CA LYS A 26 1.113 15.212 17.276 1.00 0.00 C

ATOM 393 C LYS A 26 2.435 15.967 17.206 1.00 0.00 C

ATOM 394 O LYS A 26 3.304 15.642 16.397 1.00 0.00 O

ATOM 395 CB LYS A 26 0.063 15.909 16.409 1.00 0.00 C

ATOM 396 CG LYS A 26 -1.194 15.086 16.160 1.00 0.00 C

ATOM 397 CD LYS A 26 -2.083 15.738 15.111 1.00 0.00 C

ATOM 398 CE LYS A 26 -2.719 17.015 15.640 1.00 0.00 C

ATOM 399 NZ LYS A 26 -3.600 17.658 14.627 1.00 0.00 N

ATOM 400 H LYS A 26 -0.243 15.507 18.905 1.00 0.00 H

ATOM 401 HA LYS A 26 1.281 14.209 16.882 1.00 0.00 H

ATOM 402 1HB LYS A 26 -0.237 16.844 16.882 1.00 0.00 H

ATOM 403 2HB LYS A 26 0.497 16.157 15.440 1.00 0.00 H

ATOM 404 1HG LYS A 26 -0.915 14.089 15.819 1.00 0.00 H

ATOM 405 2HG LYS A 26 -1.755 14.989 17.090 1.00 0.00 H

ATOM 406 1HD LYS A 26 -1.489 15.976 14.228 1.00 0.00 H

ATOM 407 2HD LYS A 26 -2.872 15.044 14.821 1.00 0.00 H

ATOM 408 1HE LYS A 26 -3.309 16.788 16.526 1.00 0.00 H

ATOM 409 2HE LYS A 26 -1.937 17.721 15.922 1.00 0.00 H

ATOM 410 1HZ LYS A 26 -4.001 18.500 15.015 1.00 0.00 H

ATOM 411 2HZ LYS A 26 -3.058 17.891 13.807 1.00 0.00 H

ATOM 412 3HZ LYS A 26 -4.340 17.020 14.371 1.00 0.00 H

ATOM 413 N GLU A 27 2.580 16.975 18.059 1.00 0.00 N

ATOM 414 CA GLU A 27 3.823 17.732 18.146 1.00 0.00 C

ATOM 415 C GLU A 27 4.993 16.829 18.516 1.00 0.00 C

ATOM 416 O GLU A 27 6.023 16.824 17.842 1.00 0.00 O

ATOM 417 CB GLU A 27 3.692 18.858 19.173 1.00 0.00 C

ATOM 418 CG GLU A 27 4.957 19.681 19.370 1.00 0.00 C

ATOM 419 CD GLU A 27 4.801 20.755 20.410 1.00 0.00 C

ATOM 420 OE1 GLU A 27 3.690 21.014 20.807 1.00 0.00 O

ATOM 421 OE2 GLU A 27 5.793 21.319 20.807 1.00 0.00 O

ATOM 422 H GLU A 27 1.809 17.225 18.662 1.00 0.00 H

ATOM 423 HA GLU A 27 4.030 18.172 17.169 1.00 0.00 H

ATOM 424 1HB GLU A 27 2.895 19.538 18.868 1.00 0.00 H

ATOM 425 2HB GLU A 27 3.411 18.439 20.139 1.00 0.00 H

ATOM 426 1HG GLU A 27 5.767 19.016 19.670 1.00 0.00 H

ATOM 427 2HG GLU A 27 5.232 20.138 18.420 1.00 0.00 H

ATOM 428 N GLN A 28 4.827 16.065 19.590 1.00 0.00 N

ATOM 429 CA GLN A 28 5.874 15.166 20.060 1.00 0.00 C

ATOM 430 C GLN A 28 6.215 14.119 19.008 1.00 0.00 C

ATOM 431 O GLN A 28 7.383 13.787 18.804 1.00 0.00 O

ATOM 432 CB GLN A 28 5.448 14.480 21.361 1.00 0.00 C

ATOM 433 CG GLN A 28 5.428 15.399 22.571 1.00 0.00 C

ATOM 434 CD GLN A 28 4.765 14.756 23.775 1.00 0.00 C

ATOM 435 OE1 GLN A 28 3.617 14.309 23.704 1.00 0.00 O

ATOM 436 NE2 GLN A 28 5.486 14.705 24.889 1.00 0.00 N

ATOM 437 H GLN A 28 3.952 16.107 20.094 1.00 0.00 H

ATOM 438 HA GLN A 28 6.772 15.753 20.254 1.00 0.00 H

ATOM 439 1HB GLN A 28 4.449 14.060 21.241 1.00 0.00 H

ATOM 440 2HB GLN A 28 6.126 13.655 21.577 1.00 0.00 H

ATOM 441 1HG GLN A 28 6.453 15.651 22.840 1.00 0.00 H

ATOM 442 2HG GLN A 28 4.874 16.303 22.319 1.00 0.00 H

ATOM 443 1HE2 GLN A 28 5.101 14.292 25.716 1.00 0.00 H

ATOM 444 2HE2 GLN A 28 6.413 15.079 24.903 1.00 0.00 H

ATOM 445 N LEU A 29 5.189 13.601 18.342 1.00 0.00 N

ATOM 446 CA LEU A 29 5.376 12.575 17.323 1.00 0.00 C

ATOM 447 C LEU A 29 6.236 13.089 16.175 1.00 0.00 C

ATOM 448 O LEU A 29 7.189 12.431 15.758 1.00 0.00 O

ATOM 449 CB LEU A 29 4.018 12.109 16.784 1.00 0.00 C

ATOM 450 CG LEU A 29 3.177 11.260 17.745 1.00 0.00 C

ATOM 451 CD1 LEU A 29 1.783 11.063 17.163 1.00 0.00 C

ATOM 452 CD2 LEU A 29 3.866 9.923 17.979 1.00 0.00 C

ATOM 453 H LEU A 29 4.255 13.927 18.546 1.00 0.00 H

ATOM 454 HA LEU A 29 5.883 11.725 17.778 1.00 0.00 H

ATOM 455 1HB LEU A 29 3.431 12.986 16.517 1.00 0.00 H

ATOM 456 2HB LEU A 29 4.185 11.521 15.882 1.00 0.00 H

ATOM 457 HG LEU A 29 3.070 11.784 18.696 1.00 0.00 H

ATOM 458 1HD1 LEU A 29 1.186 10.459 17.847 1.00 0.00 H

ATOM 459 2HD1 LEU A 29 1.306 12.033 17.025 1.00 0.00 H

ATOM 460 3HD1 LEU A 29 1.859 10.554 16.203 1.00 0.00 H

ATOM 461 1HD2 LEU A 29 3.268 9.320 18.663 1.00 0.00 H

ATOM 462 2HD2 LEU A 29 3.972 9.398 17.029 1.00 0.00 H

ATOM 463 3HD2 LEU A 29 4.852 10.093 18.411 1.00 0.00 H

ATOM 464 N LYS A 30 5.893 14.268 15.667 1.00 0.00 N

ATOM 465 CA LYS A 30 6.591 14.842 14.524 1.00 0.00 C

ATOM 466 C LYS A 30 8.036 15.176 14.872 1.00 0.00 C

ATOM 467 O LYS A 30 8.952 14.895 14.098 1.00 0.00 O

ATOM 468 CB LYS A 30 5.867 16.094 14.027 1.00 0.00 C

ATOM 469 CG LYS A 30 4.562 15.817 13.293 1.00 0.00 C

ATOM 470 CD LYS A 30 3.932 17.103 12.780 1.00 0.00 C

ATOM 471 CE LYS A 30 2.725 16.817 11.900 1.00 0.00 C

ATOM 472 NZ LYS A 30 2.139 18.063 11.335 1.00 0.00 N

ATOM 473 H LYS A 30 5.128 14.779 16.084 1.00 0.00 H

ATOM 474 HA LYS A 30 6.603 14.105 13.720 1.00 0.00 H

ATOM 475 1HB LYS A 30 5.643 16.745 14.873 1.00 0.00 H

ATOM 476 2HB LYS A 30 6.519 16.648 13.352 1.00 0.00 H

ATOM 477 1HG LYS A 30 4.753 15.154 12.448 1.00 0.00 H

ATOM 478 2HG LYS A 30 3.863 15.324 13.968 1.00 0.00 H

ATOM 479 1HD LYS A 30 3.617 17.717 13.626 1.00 0.00 H

ATOM 480 2HD LYS A 30 4.667 17.663 12.202 1.00 0.00 H

ATOM 481 1HE LYS A 30 3.020 16.164 11.079 1.00 0.00 H

ATOM 482 2HE LYS A 30 1.961 16.304 12.485 1.00 0.00 H

ATOM 483 1HZ LYS A 30 1.343 17.830 10.759 1.00 0.00 H

ATOM 484 2HZ LYS A 30 1.844 18.668 12.089 1.00 0.00 H

ATOM 485 3HZ LYS A 30 2.832 18.537 10.775 1.00 0.00 H

ATOM 486 N LYS A 31 8.234 15.777 16.040 1.00 0.00 N

ATOM 487 CA LYS A 31 9.562 16.199 16.469 1.00 0.00 C

ATOM 488 C LYS A 31 10.469 15.000 16.713 1.00 0.00 C

ATOM 489 O LYS A 31 11.630 14.993 16.302 1.00 0.00 O

ATOM 490 CB LYS A 31 9.470 17.054 17.733 1.00 0.00 C

ATOM 491 CG LYS A 31 8.913 18.454 17.507 1.00 0.00 C

ATOM 492 CD LYS A 31 8.833 19.234 18.810 1.00 0.00 C

ATOM 493 CE LYS A 31 8.449 20.686 18.564 1.00 0.00 C

ATOM 494 NZ LYS A 31 8.300 21.445 19.835 1.00 0.00 N

ATOM 495 H LYS A 31 7.445 15.946 16.647 1.00 0.00 H

ATOM 496 HA LYS A 31 10.008 16.798 15.674 1.00 0.00 H

ATOM 497 1HB LYS A 31 8.834 16.555 18.465 1.00 0.00 H

ATOM 498 2HB LYS A 31 10.461 17.157 18.176 1.00 0.00 H

ATOM 499 1HG LYS A 31 9.554 18.993 16.809 1.00 0.00 H

ATOM 500 2HG LYS A 31 7.915 18.382 17.075 1.00 0.00 H

ATOM 501 1HD LYS A 31 8.090 18.776 19.464 1.00 0.00 H

ATOM 502 2HD LYS A 31 9.801 19.205 19.311 1.00 0.00 H

ATOM 503 1HE LYS A 31 9.213 21.166 17.956 1.00 0.00 H

ATOM 504 2HE LYS A 31 7.505 20.725 18.020 1.00 0.00 H

ATOM 505 1HZ LYS A 31 8.045 22.401 19.629 1.00 0.00 H

ATOM 506 2HZ LYS A 31 7.579 21.019 20.400 1.00 0.00 H

ATOM 507 3HZ LYS A 31 9.175 21.432 20.340 1.00 0.00 H

ATOM 508 N GLY A 32 9.933 13.986 17.384 1.00 0.00 N

ATOM 509 CA GLY A 32 10.683 12.765 17.655 1.00 0.00 C

ATOM 510 C GLY A 32 11.059 12.051 16.364 1.00 0.00 C

ATOM 511 O GLY A 32 12.201 11.626 16.187 1.00 0.00 O

ATOM 512 H GLY A 32 8.982 14.062 17.714 1.00 0.00 H

ATOM 513 1HA GLY A 32 11.586 13.010 18.216 1.00 0.00 H

ATOM 514 2HA GLY A 32 10.086 12.103 18.281 1.00 0.00 H

ATOM 515 N LEU A 33 10.092 11.920 15.462 1.00 0.00 N

ATOM 516 CA LEU A 33 10.323 11.271 14.178 1.00 0.00 C

ATOM 517 C LEU A 33 11.357 12.030 13.356 1.00 0.00 C

ATOM 518 O LEU A 33 12.209 11.427 12.703 1.00 0.00 O

ATOM 519 CB LEU A 33 9.011 11.168 13.391 1.00 0.00 C

ATOM 520 CG LEU A 33 7.987 10.163 13.934 1.00 0.00 C

ATOM 521 CD1 LEU A 33 6.646 10.380 13.245 1.00 0.00 C

ATOM 522 CD2 LEU A 33 8.495 8.747 13.706 1.00 0.00 C

ATOM 523 H LEU A 33 9.172 12.280 15.673 1.00 0.00 H

ATOM 524 HA LEU A 33 10.701 10.266 14.362 1.00 0.00 H

ATOM 525 1HB LEU A 33 8.537 12.148 13.377 1.00 0.00 H

ATOM 526 2HB LEU A 33 9.242 10.884 12.365 1.00 0.00 H

ATOM 527 HG LEU A 33 7.844 10.330 15.002 1.00 0.00 H

ATOM 528 1HD1 LEU A 33 5.919 9.666 13.631 1.00 0.00 H

ATOM 529 2HD1 LEU A 33 6.297 11.394 13.441 1.00 0.00 H

ATOM 530 3HD1 LEU A 33 6.762 10.236 12.172 1.00 0.00 H

ATOM 531 1HD2 LEU A 33 7.768 8.032 14.093 1.00 0.00 H

ATOM 532 2HD2 LEU A 33 8.637 8.579 12.638 1.00 0.00 H

ATOM 533 3HD2 LEU A 33 9.445 8.614 14.224 1.00 0.00 H

ATOM 534 N GLU A 34 11.277 13.355 13.392 1.00 0.00 N

ATOM 535 CA GLU A 34 12.188 14.199 12.627 1.00 0.00 C

ATOM 536 C GLU A 34 13.623 14.042 13.113 1.00 0.00 C

ATOM 537 O GLU A 34 14.560 14.026 12.315 1.00 0.00 O

ATOM 538 CB GLU A 34 11.764 15.666 12.724 1.00 0.00 C

ATOM 539 CG GLU A 34 12.618 16.623 11.904 1.00 0.00 C

ATOM 540 CD GLU A 34 12.529 16.367 10.426 1.00 0.00 C

ATOM 541 OE1 GLU A 34 11.573 15.761 10.004 1.00 0.00 O

ATOM 542 OE2 GLU A 34 13.418 16.777 9.717 1.00 0.00 O

ATOM 543 H GLU A 34 10.567 13.790 13.964 1.00 0.00 H

ATOM 544 HA GLU A 34 12.148 13.892 11.582 1.00 0.00 H

ATOM 545 1HB GLU A 34 10.731 15.770 12.390 1.00 0.00 H

ATOM 546 2HB GLU A 34 11.805 15.989 13.765 1.00 0.00 H

ATOM 547 1HG GLU A 34 12.295 17.644 12.104 1.00 0.00 H

ATOM 548 2HG GLU A 34 13.656 16.531 12.223 1.00 0.00 H

ATOM 549 N LYS A 35 13.788 13.927 14.426 1.00 0.00 N

ATOM 550 CA LYS A 35 15.102 13.694 15.015 1.00 0.00 C

ATOM 551 C LYS A 35 15.683 12.362 14.557 1.00 0.00 C

ATOM 552 O LYS A 35 16.874 12.262 14.265 1.00 0.00 O

ATOM 553 CB LYS A 35 15.019 13.733 16.542 1.00 0.00 C

ATOM 554 CG LYS A 35 14.790 15.122 17.124 1.00 0.00 C

ATOM 555 CD LYS A 35 14.668 15.073 18.639 1.00 0.00 C

ATOM 556 CE LYS A 35 14.421 16.457 19.221 1.00 0.00 C

ATOM 557 NZ LYS A 35 14.283 16.423 20.702 1.00 0.00 N

ATOM 558 H LYS A 35 12.984 14.002 15.032 1.00 0.00 H

ATOM 559 HA LYS A 35 15.775 14.484 14.682 1.00 0.00 H

ATOM 560 1HB LYS A 35 14.204 13.091 16.879 1.00 0.00 H

ATOM 561 2HB LYS A 35 15.943 13.341 16.967 1.00 0.00 H

ATOM 562 1HG LYS A 35 15.624 15.771 16.855 1.00 0.00 H

ATOM 563 2HG LYS A 35 13.876 15.544 16.708 1.00 0.00 H

ATOM 564 1HD LYS A 35 13.841 14.418 18.917 1.00 0.00 H

ATOM 565 2HD LYS A 35 15.587 14.670 19.065 1.00 0.00 H

ATOM 566 1HE LYS A 35 15.250 17.114 18.960 1.00 0.00 H

ATOM 567 2HE LYS A 35 13.509 16.874 18.793 1.00 0.00 H

ATOM 568 1HZ LYS A 35 14.120 17.358 21.047 1.00 0.00 H

ATOM 569 2HZ LYS A 35 13.504 15.830 20.954 1.00 0.00 H

ATOM 570 3HZ LYS A 35 15.131 16.056 21.111 1.00 0.00 H

ATOM 571 N ASP A 36 14.834 11.342 14.495 1.00 0.00 N

ATOM 572 CA ASP A 36 15.251 10.026 14.026 1.00 0.00 C

ATOM 573 C ASP A 36 15.482 10.026 12.520 1.00 0.00 C

ATOM 574 O ASP A 36 16.327 9.289 12.012 1.00 0.00 O

ATOM 575 CB ASP A 36 14.202 8.971 14.387 1.00 0.00 C

ATOM 576 CG ASP A 36 14.179 8.644 15.874 1.00 0.00 C

ATOM 577 OD1 ASP A 36 15.086 9.043 16.565 1.00 0.00 O

ATOM 578 OD2 ASP A 36 13.253 7.999 16.305 1.00 0.00 O

ATOM 579 H ASP A 36 13.875 11.481 14.781 1.00 0.00 H

ATOM 580 HA ASP A 36 16.190 9.765 14.515 1.00 0.00 H

ATOM 581 1HB ASP A 36 13.214 9.323 14.092 1.00 0.00 H

ATOM 582 2HB ASP A 36 14.402 8.054 13.832 1.00 0.00 H

ATOM 583 N GLY A 37 14.725 10.856 11.811 1.00 0.00 N

ATOM 584 CA GLY A 37 14.865 10.974 10.364 1.00 0.00 C

ATOM 585 C GLY A 37 13.739 10.247 9.641 1.00 0.00 C

ATOM 586 O GLY A 37 13.888 9.842 8.488 1.00 0.00 O

ATOM 587 H GLY A 37 14.035 11.421 12.285 1.00 0.00 H

ATOM 588 1HA GLY A 37 14.863 12.027 10.082 1.00 0.00 H

ATOM 589 2HA GLY A 37 15.826 10.561 10.057 1.00 0.00 H

ATOM 590 N LEU A 38 12.612 10.083 10.325 1.00 0.00 N

ATOM 591 CA LEU A 38 11.454 9.413 9.745 1.00 0.00 C

ATOM 592 C LEU A 38 10.407 10.420 9.286 1.00 0.00 C

ATOM 593 O LEU A 38 10.257 11.488 9.879 1.00 0.00 O

ATOM 594 CB LEU A 38 10.833 8.450 10.765 1.00 0.00 C

ATOM 595 CG LEU A 38 11.757 7.338 11.278 1.00 0.00 C

ATOM 596 CD1 LEU A 38 11.036 6.531 12.349 1.00 0.00 C

ATOM 597 CD2 LEU A 38 12.177 6.450 10.115 1.00 0.00 C

ATOM 598 H LEU A 38 12.555 10.432 11.271 1.00 0.00 H

ATOM 599 HA LEU A 38 11.784 8.841 8.879 1.00 0.00 H

ATOM 600 1HB LEU A 38 10.498 9.025 11.627 1.00 0.00 H

ATOM 601 2HB LEU A 38 9.964 7.975 10.311 1.00 0.00 H

ATOM 602 HG LEU A 38 12.643 7.781 11.734 1.00 0.00 H

ATOM 603 1HD1 LEU A 38 11.693 5.741 12.713 1.00 0.00 H

ATOM 604 2HD1 LEU A 38 10.765 7.187 13.177 1.00 0.00 H

ATOM 605 3HD1 LEU A 38 10.135 6.088 11.926 1.00 0.00 H

ATOM 606 1HD2 LEU A 38 12.834 5.660 10.479 1.00 0.00 H

ATOM 607 2HD2 LEU A 38 11.292 6.005 9.659 1.00 0.00 H

ATOM 608 3HD2 LEU A 38 12.706 7.049 9.373 1.00 0.00 H

ATOM 609 N LYS A 39 9.685 10.073 8.226 1.00 0.00 N

ATOM 610 CA LYS A 39 8.673 10.960 7.665 1.00 0.00 C

ATOM 611 C LYS A 39 7.293 10.316 7.702 1.00 0.00 C

ATOM 612 O LYS A 39 7.167 9.108 7.903 1.00 0.00 O

ATOM 613 CB LYS A 39 9.036 11.345 6.230 1.00 0.00 C

ATOM 614 CG LYS A 39 10.375 12.055 6.088 1.00 0.00 C

ATOM 615 CD LYS A 39 10.348 13.424 6.752 1.00 0.00 C

ATOM 616 CE LYS A 39 11.654 14.173 6.532 1.00 0.00 C

ATOM 617 NZ LYS A 39 11.642 15.515 7.175 1.00 0.00 N

ATOM 618 H LYS A 39 9.840 9.171 7.799 1.00 0.00 H

ATOM 619 HA LYS A 39 8.634 11.867 8.269 1.00 0.00 H

ATOM 620 1HB LYS A 39 9.065 10.449 5.609 1.00 0.00 H

ATOM 621 2HB LYS A 39 8.265 12.000 5.823 1.00 0.00 H

ATOM 622 1HG LYS A 39 11.158 11.452 6.549 1.00 0.00 H

ATOM 623 2HG LYS A 39 10.612 12.178 5.031 1.00 0.00 H

ATOM 624 1HD LYS A 39 9.528 14.012 6.340 1.00 0.00 H

ATOM 625 2HD LYS A 39 10.185 13.305 7.823 1.00 0.00 H

ATOM 626 1HE LYS A 39 12.479 13.594 6.944 1.00 0.00 H

ATOM 627 2HE LYS A 39 11.826 14.298 5.463 1.00 0.00 H

ATOM 628 1HZ LYS A 39 12.523 15.978 7.006 1.00 0.00 H

ATOM 629 2HZ LYS A 39 10.891 16.068 6.787 1.00 0.00 H

ATOM 630 3HZ LYS A 39 11.502 15.410 8.170 1.00 0.00 H

ATOM 631 N LEU A 40 6.261 11.129 7.507 1.00 0.00 N

ATOM 632 CA LEU A 40 4.887 10.644 7.540 1.00 0.00 C

ATOM 633 C LEU A 40 4.156 10.970 6.243 1.00 0.00 C

ATOM 634 O LEU A 40 4.483 11.943 5.564 1.00 0.00 O

ATOM 635 CB LEU A 40 4.135 11.260 8.726 1.00 0.00 C

ATOM 636 CG LEU A 40 4.686 10.915 10.115 1.00 0.00 C

ATOM 637 CD1 LEU A 40 3.998 11.778 11.164 1.00 0.00 C

ATOM 638 CD2 LEU A 40 4.468 9.436 10.395 1.00 0.00 C

ATOM 639 H LEU A 40 6.433 12.109 7.332 1.00 0.00 H

ATOM 640 HA LEU A 40 4.905 9.561 7.661 1.00 0.00 H

ATOM 641 1HB LEU A 40 4.152 12.344 8.623 1.00 0.00 H

ATOM 642 2HB LEU A 40 3.097 10.929 8.690 1.00 0.00 H

ATOM 643 HG LEU A 40 5.753 11.137 10.148 1.00 0.00 H

ATOM 644 1HD1 LEU A 40 4.390 11.533 12.151 1.00 0.00 H

ATOM 645 2HD1 LEU A 40 4.187 12.830 10.951 1.00 0.00 H

ATOM 646 3HD1 LEU A 40 2.925 11.590 11.143 1.00 0.00 H

ATOM 647 1HD2 LEU A 40 4.861 9.190 11.382 1.00 0.00 H

ATOM 648 2HD2 LEU A 40 3.401 9.213 10.363 1.00 0.00 H

ATOM 649 3HD2 LEU A 40 4.985 8.842 9.641 1.00 0.00 H

ATOM 650 N PRO A 41 3.167 10.150 5.905 1.00 0.00 N

ATOM 651 CA PRO A 41 2.337 10.394 4.731 1.00 0.00 C

ATOM 652 C PRO A 41 1.739 11.795 4.761 1.00 0.00 C

ATOM 653 O PRO A 41 1.458 12.338 5.830 1.00 0.00 O

ATOM 654 CB PRO A 41 1.255 9.316 4.844 1.00 0.00 C

ATOM 655 CG PRO A 41 1.913 8.215 5.603 1.00 0.00 C

ATOM 656 CD PRO A 41 2.779 8.922 6.612 1.00 0.00 C

ATOM 657 HA PRO A 41 2.942 10.251 3.824 1.00 0.00 H

ATOM 658 1HB PRO A 41 0.372 9.721 5.360 1.00 0.00 H

ATOM 659 2HB PRO A 41 0.926 9.006 3.841 1.00 0.00 H

ATOM 660 1HG PRO A 41 1.155 7.571 6.073 1.00 0.00 H

ATOM 661 2HG PRO A 41 2.494 7.577 4.920 1.00 0.00 H

ATOM 662 1HD PRO A 41 2.188 9.139 7.515 1.00 0.00 H

ATOM 663 2HD PRO A 41 3.646 8.291 6.859 1.00 0.00 H

ATOM 664 N TYR A 42 1.547 12.376 3.582 1.00 0.00 N

ATOM 665 CA TYR A 42 1.059 13.746 3.473 1.00 0.00 C

ATOM 666 C TYR A 42 -0.263 13.919 4.210 1.00 0.00 C

ATOM 667 O TYR A 42 -0.471 14.912 4.908 1.00 0.00 O

ATOM 668 CB TYR A 42 0.903 14.143 2.003 1.00 0.00 C

ATOM 669 CG TYR A 42 0.405 15.558 1.803 1.00 0.00 C

ATOM 670 CD1 TYR A 42 1.273 16.628 1.967 1.00 0.00 C

ATOM 671 CD2 TYR A 42 -0.918 15.784 1.455 1.00 0.00 C

ATOM 672 CE1 TYR A 42 0.818 17.919 1.784 1.00 0.00 C

ATOM 673 CE2 TYR A 42 -1.372 17.076 1.272 1.00 0.00 C

ATOM 674 CZ TYR A 42 -0.510 18.141 1.436 1.00 0.00 C

ATOM 675 OH TYR A 42 -0.962 19.427 1.253 1.00 0.00 O

ATOM 676 H TYR A 42 1.745 11.856 2.739 1.00 0.00 H

ATOM 677 HA TYR A 42 1.786 14.411 3.939 1.00 0.00 H

ATOM 678 1HB TYR A 42 1.864 14.045 1.496 1.00 0.00 H

ATOM 679 2HB TYR A 42 0.204 13.465 1.515 1.00 0.00 H

ATOM 680 HD1 TYR A 42 2.313 16.449 2.241 1.00 0.00 H

ATOM 681 HD2 TYR A 42 -1.600 14.944 1.327 1.00 0.00 H

ATOM 682 HE1 TYR A 42 1.499 18.760 1.913 1.00 0.00 H

ATOM 683 HE2 TYR A 42 -2.413 17.254 1.000 1.00 0.00 H

ATOM 684 HH TYR A 42 -1.894 19.408 1.022 1.00 0.00 H

ATOM 685 N ASN A 43 -1.155 12.947 4.051 1.00 0.00 N

ATOM 686 CA ASN A 43 -2.494 13.036 4.621 1.00 0.00 C

ATOM 687 C ASN A 43 -2.574 12.307 5.956 1.00 0.00 C

ATOM 688 O ASN A 43 -3.662 11.999 6.443 1.00 0.00 O

ATOM 689 CB ASN A 43 -3.525 12.487 3.651 1.00 0.00 C

ATOM 690 CG ASN A 43 -3.707 13.364 2.444 1.00 0.00 C

ATOM 691 OD1 ASN A 43 -3.824 14.589 2.563 1.00 0.00 O

ATOM 692 ND2 ASN A 43 -3.733 12.761 1.283 1.00 0.00 N

ATOM 693 H ASN A 43 -0.899 12.126 3.521 1.00 0.00 H

ATOM 694 HA ASN A 43 -2.721 14.086 4.811 1.00 0.00 H

ATOM 695 1HB ASN A 43 -3.221 11.492 3.321 1.00 0.00 H

ATOM 696 2HB ASN A 43 -4.484 12.384 4.159 1.00 0.00 H

ATOM 697 1HD2 ASN A 43 -3.852 13.294 0.445 1.00 0.00 H

ATOM 698 2HD2 ASN A 43 -3.634 11.767 1.233 1.00 0.00 H

ATOM 699 N PHE A 44 -1.414 12.035 6.546 1.00 0.00 N

ATOM 700 CA PHE A 44 -1.349 11.313 7.811 1.00 0.00 C

ATOM 701 C PHE A 44 -1.957 12.130 8.943 1.00 0.00 C

ATOM 702 O PHE A 44 -2.628 11.588 9.822 1.00 0.00 O

ATOM 703 CB PHE A 44 0.101 10.961 8.151 1.00 0.00 C

ATOM 704 CG PHE A 44 0.236 9.956 9.259 1.00 0.00 C

ATOM 705 CD1 PHE A 44 -0.055 8.618 9.041 1.00 0.00 C

ATOM 706 CD2 PHE A 44 0.655 10.347 10.522 1.00 0.00 C

ATOM 707 CE1 PHE A 44 0.069 7.693 10.061 1.00 0.00 C

ATOM 708 CE2 PHE A 44 0.781 9.425 11.542 1.00 0.00 C

ATOM 709 CZ PHE A 44 0.487 8.096 11.311 1.00 0.00 C

ATOM 710 H PHE A 44 -0.556 12.336 6.106 1.00 0.00 H

ATOM 711 HA PHE A 44 -1.918 10.388 7.711 1.00 0.00 H

ATOM 712 1HB PHE A 44 0.595 10.560 7.267 1.00 0.00 H

ATOM 713 2HB PHE A 44 0.635 11.864 8.443 1.00 0.00 H

ATOM 714 HD1 PHE A 44 -0.385 8.299 8.052 1.00 0.00 H

ATOM 715 HD2 PHE A 44 0.887 11.397 10.705 1.00 0.00 H

ATOM 716 HE1 PHE A 44 -0.164 6.645 9.876 1.00 0.00 H

ATOM 717 HE2 PHE A 44 1.112 9.745 12.530 1.00 0.00 H

ATOM 718 HZ PHE A 44 0.584 7.368 12.115 1.00 0.00 H

ATOM 719 N ASP A 45 -1.720 13.437 8.917 1.00 0.00 N

ATOM 720 CA ASP A 45 -2.283 14.338 9.915 1.00 0.00 C

ATOM 721 C ASP A 45 -3.803 14.243 9.948 1.00 0.00 C

ATOM 722 O ASP A 45 -4.414 14.287 11.015 1.00 0.00 O

ATOM 723 CB ASP A 45 -1.862 15.782 9.632 1.00 0.00 C

ATOM 724 CG ASP A 45 -0.405 16.054 9.981 1.00 0.00 C

ATOM 725 OD1 ASP A 45 0.207 15.210 10.591 1.00 0.00 O

ATOM 726 OD2 ASP A 45 0.082 17.103 9.633 1.00 0.00 O

ATOM 727 H ASP A 45 -1.133 13.816 8.187 1.00 0.00 H

ATOM 728 HA ASP A 45 -1.902 14.050 10.896 1.00 0.00 H

ATOM 729 1HB ASP A 45 -2.016 16.006 8.576 1.00 0.00 H

ATOM 730 2HB ASP A 45 -2.491 16.464 10.206 1.00 0.00 H

ATOM 731 N LEU A 46 -4.408 14.112 8.772 1.00 0.00 N

ATOM 732 CA LEU A 46 -5.852 13.941 8.669 1.00 0.00 C

ATOM 733 C LEU A 46 -6.302 12.645 9.331 1.00 0.00 C

ATOM 734 O LEU A 46 -7.335 12.604 9.999 1.00 0.00 O

ATOM 735 CB LEU A 46 -6.282 13.946 7.196 1.00 0.00 C

ATOM 736 CG LEU A 46 -6.130 15.285 6.463 1.00 0.00 C

ATOM 737 CD1 LEU A 46 -6.420 15.089 4.982 1.00 0.00 C

ATOM 738 CD2 LEU A 46 -7.075 16.308 7.075 1.00 0.00 C

ATOM 739 H LEU A 46 -3.853 14.132 7.929 1.00 0.00 H

ATOM 740 HA LEU A 46 -6.336 14.773 9.178 1.00 0.00 H

ATOM 741 1HB LEU A 46 -5.691 13.205 6.659 1.00 0.00 H

ATOM 742 2HB LEU A 46 -7.331 13.654 7.139 1.00 0.00 H

ATOM 743 HG LEU A 46 -5.103 15.637 6.558 1.00 0.00 H

ATOM 744 1HD1 LEU A 46 -6.312 16.040 4.461 1.00 0.00 H

ATOM 745 2HD1 LEU A 46 -5.718 14.366 4.565 1.00 0.00 H

ATOM 746 3HD1 LEU A 46 -7.438 14.720 4.856 1.00 0.00 H

ATOM 747 1HD2 LEU A 46 -6.966 17.260 6.555 1.00 0.00 H

ATOM 748 2HD2 LEU A 46 -8.103 15.958 6.980 1.00 0.00 H

ATOM 749 3HD2 LEU A 46 -6.833 16.442 8.130 1.00 0.00 H

ATOM 750 N LEU A 47 -5.520 11.587 9.143 1.00 0.00 N

ATOM 751 CA LEU A 47 -5.778 10.315 9.806 1.00 0.00 C

ATOM 752 C LEU A 47 -5.664 10.450 11.319 1.00 0.00 C

ATOM 753 O LEU A 47 -6.449 9.864 12.065 1.00 0.00 O

ATOM 754 CB LEU A 47 -4.797 9.248 9.306 1.00 0.00 C

ATOM 755 CG LEU A 47 -4.965 8.823 7.842 1.00 0.00 C

ATOM 756 CD1 LEU A 47 -3.812 7.913 7.441 1.00 0.00 C

ATOM 757 CD2 LEU A 47 -6.303 8.120 7.668 1.00 0.00 C

ATOM 758 H LEU A 47 -4.725 11.667 8.524 1.00 0.00 H

ATOM 759 HA LEU A 47 -6.793 9.999 9.565 1.00 0.00 H

ATOM 760 1HB LEU A 47 -3.782 9.624 9.427 1.00 0.00 H

ATOM 761 2HB LEU A 47 -4.908 8.357 9.925 1.00 0.00 H

ATOM 762 HG LEU A 47 -4.932 9.705 7.201 1.00 0.00 H

ATOM 763 1HD1 LEU A 47 -3.931 7.611 6.400 1.00 0.00 H

ATOM 764 2HD1 LEU A 47 -2.869 8.448 7.557 1.00 0.00 H

ATOM 765 3HD1 LEU A 47 -3.809 7.028 8.077 1.00 0.00 H

ATOM 766 1HD2 LEU A 47 -6.423 7.818 6.626 1.00 0.00 H

ATOM 767 2HD2 LEU A 47 -6.336 7.237 8.307 1.00 0.00 H

ATOM 768 3HD2 LEU A 47 -7.110 8.798 7.944 1.00 0.00 H

ATOM 769 N LEU A 48 -4.682 11.225 11.767 1.00 0.00 N

ATOM 770 CA LEU A 48 -4.458 11.431 13.193 1.00 0.00 C

ATOM 771 C LEU A 48 -5.623 12.177 13.831 1.00 0.00 C

ATOM 772 O LEU A 48 -6.006 11.894 14.966 1.00 0.00 O

ATOM 773 CB LEU A 48 -3.158 12.213 13.417 1.00 0.00 C

ATOM 774 CG LEU A 48 -1.865 11.473 13.051 1.00 0.00 C

ATOM 775 CD1 LEU A 48 -0.676 12.406 13.236 1.00 0.00 C

ATOM 776 CD2 LEU A 48 -1.726 10.232 13.919 1.00 0.00 C

ATOM 777 H LEU A 48 -4.074 11.682 11.102 1.00 0.00 H

ATOM 778 HA LEU A 48 -4.369 10.457 13.673 1.00 0.00 H

ATOM 779 1HB LEU A 48 -3.195 13.127 12.826 1.00 0.00 H

ATOM 780 2HB LEU A 48 -3.095 12.488 14.470 1.00 0.00 H

ATOM 781 HG LEU A 48 -1.898 11.180 12.001 1.00 0.00 H

ATOM 782 1HD1 LEU A 48 0.243 11.881 12.975 1.00 0.00 H

ATOM 783 2HD1 LEU A 48 -0.791 13.276 12.589 1.00 0.00 H

ATOM 784 3HD1 LEU A 48 -0.626 12.730 14.275 1.00 0.00 H

ATOM 785 1HD2 LEU A 48 -0.808 9.706 13.658 1.00 0.00 H

ATOM 786 2HD2 LEU A 48 -1.691 10.524 14.969 1.00 0.00 H

ATOM 787 3HD2 LEU A 48 -2.580 9.575 13.754 1.00 0.00 H

ATOM 788 N ASP A 49 -6.183 13.131 13.095 1.00 0.00 N

ATOM 789 CA ASP A 49 -7.340 13.882 13.565 1.00 0.00 C

ATOM 790 C ASP A 49 -8.582 13.001 13.625 1.00 0.00 C

ATOM 791 O ASP A 49 -9.423 13.156 14.510 1.00 0.00 O

ATOM 792 CB ASP A 49 -7.604 15.085 12.657 1.00 0.00 C

ATOM 793 CG ASP A 49 -6.568 16.189 12.820 1.00 0.00 C

ATOM 794 OD1 ASP A 49 -5.820 16.140 13.767 1.00 0.00 O

ATOM 795 OD2 ASP A 49 -6.534 17.070 11.994 1.00 0.00 O

ATOM 796 H ASP A 49 -5.797 13.341 12.185 1.00 0.00 H

ATOM 797 HA ASP A 49 -7.131 14.244 14.572 1.00 0.00 H

ATOM 798 1HB ASP A 49 -7.609 14.761 11.616 1.00 0.00 H

ATOM 799 2HB ASP A 49 -8.590 15.497 12.876 1.00 0.00 H

ATOM 800 N GLN A 50 -8.690 12.075 12.677 1.00 0.00 N

ATOM 801 CA GLN A 50 -9.756 11.081 12.697 1.00 0.00 C

ATOM 802 C GLN A 50 -9.621 10.152 13.897 1.00 0.00 C

ATOM 803 O GLN A 50 -10.617 9.761 14.506 1.00 0.00 O

ATOM 804 CB GLN A 50 -9.750 10.264 11.402 1.00 0.00 C

ATOM 805 CG GLN A 50 -10.199 11.040 10.175 1.00 0.00 C

ATOM 806 CD GLN A 50 -10.063 10.232 8.898 1.00 0.00 C

ATOM 807 OE1 GLN A 50 -9.489 9.140 8.896 1.00 0.00 O

ATOM 808 NE2 GLN A 50 -10.592 10.766 7.803 1.00 0.00 N

ATOM 809 H GLN A 50 -8.017 12.061 11.925 1.00 0.00 H

ATOM 810 HA GLN A 50 -10.711 11.599 12.778 1.00 0.00 H

ATOM 811 1HB GLN A 50 -8.745 9.888 11.213 1.00 0.00 H

ATOM 812 2HB GLN A 50 -10.407 9.401 11.513 1.00 0.00 H

ATOM 813 1HG GLN A 50 -11.247 11.316 10.295 1.00 0.00 H

ATOM 814 2HG GLN A 50 -9.585 11.936 10.080 1.00 0.00 H

ATOM 815 1HE2 GLN A 50 -10.533 10.279 6.930 1.00 0.00 H

ATOM 816 2HE2 GLN A 50 -11.049 11.654 7.849 1.00 0.00 H

ATOM 817 N ILE A 51 -8.384 9.803 14.233 1.00 0.00 N

ATOM 818 CA ILE A 51 -8.110 8.998 15.417 1.00 0.00 C

ATOM 819 C ILE A 51 -8.524 9.729 16.688 1.00 0.00 C

ATOM 820 O ILE A 51 -9.240 9.181 17.525 1.00 0.00 O

ATOM 821 CB ILE A 51 -6.617 8.631 15.498 1.00 0.00 C

ATOM 822 CG1 ILE A 51 -6.245 7.657 14.377 1.00 0.00 C

ATOM 823 CG2 ILE A 51 -6.289 8.033 16.858 1.00 0.00 C

ATOM 824 CD1 ILE A 51 -4.755 7.475 14.196 1.00 0.00 C

ATOM 825 H ILE A 51 -7.614 10.102 13.652 1.00 0.00 H

ATOM 826 HA ILE A 51 -8.688 8.077 15.350 1.00 0.00 H

ATOM 827 HB ILE A 51 -6.014 9.527 15.351 1.00 0.00 H

ATOM 828 1HG1 ILE A 51 -6.685 6.682 14.581 1.00 0.00 H

ATOM 829 2HG1 ILE A 51 -6.661 8.012 13.434 1.00 0.00 H

ATOM 830 1HG2 ILE A 51 -5.230 7.779 16.898 1.00 0.00 H

ATOM 831 2HG2 ILE A 51 -6.518 8.758 17.638 1.00 0.00 H

ATOM 832 3HG2 ILE A 51 -6.884 7.133 17.012 1.00 0.00 H

ATOM 833 1HD1 ILE A 51 -4.570 6.772 13.384 1.00 0.00 H

ATOM 834 2HD1 ILE A 51 -4.297 8.436 13.955 1.00 0.00 H

ATOM 835 3HD1 ILE A 51 -4.321 7.087 15.116 1.00 0.00 H

ATOM 836 N ASP A 52 -8.068 10.969 16.826 1.00 0.00 N

ATOM 837 CA ASP A 52 -8.327 11.752 18.028 1.00 0.00 C

ATOM 838 C ASP A 52 -9.668 12.470 17.940 1.00 0.00 C

ATOM 839 O ASP A 52 -9.732 13.697 18.018 1.00 0.00 O

ATOM 840 CB ASP A 52 -7.209 12.772 18.257 1.00 0.00 C

ATOM 841 CG ASP A 52 -7.305 13.463 19.610 1.00 0.00 C

ATOM 842 OD1 ASP A 52 -7.968 12.942 20.476 1.00 0.00 O

ATOM 843 OD2 ASP A 52 -6.713 14.504 19.766 1.00 0.00 O

ATOM 844 H ASP A 52 -7.528 11.381 16.078 1.00 0.00 H

ATOM 845 HA ASP A 52 -8.360 11.074 18.882 1.00 0.00 H

ATOM 846 1HB ASP A 52 -6.242 12.273 18.187 1.00 0.00 H

ATOM 847 2HB ASP A 52 -7.244 13.531 17.474 1.00 0.00 H

ATOM 848 N SER A 53 -10.737 11.698 17.776 1.00 0.00 N

ATOM 849 CA SER A 53 -12.070 12.263 17.606 1.00 0.00 C

ATOM 850 C SER A 53 -12.581 12.871 18.906 1.00 0.00 C

ATOM 851 O SER A 53 -13.522 13.664 18.904 1.00 0.00 O

ATOM 852 CB SER A 53 -13.033 11.194 17.126 1.00 0.00 C

ATOM 853 OG SER A 53 -13.254 10.234 18.122 1.00 0.00 O

ATOM 854 H SER A 53 -10.623 10.695 17.770 1.00 0.00 H

ATOM 855 HA SER A 53 -12.019 13.053 16.856 1.00 0.00 H

ATOM 856 1HB SER A 53 -13.979 11.656 16.845 1.00 0.00 H

ATOM 857 2HB SER A 53 -12.627 10.713 16.237 1.00 0.00 H

ATOM 858 HG SER A 53 -13.389 10.723 18.936 1.00 0.00 H

ATOM 859 N ASP A 54 -11.954 12.496 20.016 1.00 0.00 N

ATOM 860 CA ASP A 54 -12.341 13.007 21.325 1.00 0.00 C

ATOM 861 C ASP A 54 -11.739 14.383 21.579 1.00 0.00 C

ATOM 862 O ASP A 54 -12.177 15.107 22.473 1.00 0.00 O

ATOM 863 CB ASP A 54 -11.904 12.041 22.429 1.00 0.00 C

ATOM 864 CG ASP A 54 -12.657 10.718 22.390 1.00 0.00 C

ATOM 865 OD1 ASP A 54 -13.705 10.670 21.790 1.00 0.00 O

ATOM 866 OD2 ASP A 54 -12.177 9.767 22.961 1.00 0.00 O

ATOM 867 H ASP A 54 -11.189 11.839 19.950 1.00 0.00 H

ATOM 868 HA ASP A 54 -13.427 13.100 21.353 1.00 0.00 H

ATOM 869 1HB ASP A 54 -10.837 11.838 22.334 1.00 0.00 H

ATOM 870 2HB ASP A 54 -12.064 12.504 23.403 1.00 0.00 H

ATOM 871 N GLY A 55 -10.732 14.738 20.788 1.00 0.00 N

ATOM 872 CA GLY A 55 -10.039 16.009 20.954 1.00 0.00 C

ATOM 873 C GLY A 55 -9.163 16.000 22.201 1.00 0.00 C

ATOM 874 O GLY A 55 -9.094 16.990 22.930 1.00 0.00 O

ATOM 875 H GLY A 55 -10.440 14.111 20.052 1.00 0.00 H

ATOM 876 1HA GLY A 55 -9.424 16.207 20.075 1.00 0.00 H

ATOM 877 2HA GLY A 55 -10.768 16.815 21.024 1.00 0.00 H

ATOM 878 N SER A 56 -8.496 14.877 22.441 1.00 0.00 N

ATOM 879 CA SER A 56 -7.656 14.722 23.623 1.00 0.00 C

ATOM 880 C SER A 56 -6.304 15.395 23.430 1.00 0.00 C

ATOM 881 O SER A 56 -5.667 15.820 24.394 1.00 0.00 O

ATOM 882 CB SER A 56 -7.459 13.251 23.934 1.00 0.00 C

ATOM 883 OG SER A 56 -6.676 12.631 22.952 1.00 0.00 O

ATOM 884 H SER A 56 -8.572 14.110 21.788 1.00 0.00 H

ATOM 885 HA SER A 56 -8.157 15.197 24.468 1.00 0.00 H

ATOM 886 1HB SER A 56 -6.978 13.147 24.907 1.00 0.00 H

ATOM 887 2HB SER A 56 -8.429 12.759 23.994 1.00 0.00 H

ATOM 888 HG SER A 56 -7.164 12.718 22.130 1.00 0.00 H

ATOM 889 N GLY A 57 -5.869 15.490 22.178 1.00 0.00 N

ATOM 890 CA GLY A 57 -4.576 16.085 21.858 1.00 0.00 C

ATOM 891 C GLY A 57 -3.511 15.014 21.662 1.00 0.00 C

ATOM 892 O GLY A 57 -2.410 15.299 21.192 1.00 0.00 O

ATOM 893 H GLY A 57 -6.449 15.141 21.428 1.00 0.00 H

ATOM 894 1HA GLY A 57 -4.666 16.685 20.952 1.00 0.00 H

ATOM 895 2HA GLY A 57 -4.277 16.757 22.661 1.00 0.00 H

ATOM 896 N LYS A 58 -3.845 13.780 22.024 1.00 0.00 N

ATOM 897 CA LYS A 58 -2.893 12.678 21.958 1.00 0.00 C

ATOM 898 C LYS A 58 -3.391 11.575 21.033 1.00 0.00 C

ATOM 899 O LYS A 58 -4.595 11.413 20.835 1.00 0.00 O

ATOM 900 CB LYS A 58 -2.630 12.113 23.355 1.00 0.00 C

ATOM 901 CG LYS A 58 -1.942 13.084 24.306 1.00 0.00 C

ATOM 902 CD LYS A 58 -1.673 12.437 25.656 1.00 0.00 C

ATOM 903 CE LYS A 58 -0.981 13.403 26.607 1.00 0.00 C

ATOM 904 NZ LYS A 58 -0.726 12.789 27.937 1.00 0.00 N

ATOM 905 H LYS A 58 -4.783 13.601 22.354 1.00 0.00 H

ATOM 906 HA LYS A 58 -1.954 13.055 21.552 1.00 0.00 H

ATOM 907 1HB LYS A 58 -3.574 11.812 23.810 1.00 0.00 H

ATOM 908 2HB LYS A 58 -2.005 11.223 23.276 1.00 0.00 H

ATOM 909 1HG LYS A 58 -0.995 13.409 23.873 1.00 0.00 H

ATOM 910 2HG LYS A 58 -2.574 13.959 24.452 1.00 0.00 H

ATOM 911 1HD LYS A 58 -2.616 12.115 26.101 1.00 0.00 H

ATOM 912 2HD LYS A 58 -1.038 11.561 25.520 1.00 0.00 H

ATOM 913 1HE LYS A 58 -0.032 13.718 26.177 1.00 0.00 H

ATOM 914 2HE LYS A 58 -1.604 14.287 26.743 1.00 0.00 H

ATOM 915 1HZ LYS A 58 -0.268 13.461 28.537 1.00 0.00 H

ATOM 916 2HZ LYS A 58 -1.603 12.509 28.354 1.00 0.00 H

ATOM 917 3HZ LYS A 58 -0.133 11.979 27.826 1.00 0.00 H

ATOM 918 N ILE A 59 -2.457 10.817 20.468 1.00 0.00 N

ATOM 919 CA ILE A 59 -2.796 9.760 19.523 1.00 0.00 C

ATOM 920 C ILE A 59 -2.596 8.383 20.141 1.00 0.00 C

ATOM 921 O ILE A 59 -1.529 8.080 20.675 1.00 0.00 O

ATOM 922 CB ILE A 59 -1.949 9.876 18.242 1.00 0.00 C

ATOM 923 CG1 ILE A 59 -2.084 11.276 17.638 1.00 0.00 C

ATOM 924 CG2 ILE A 59 -2.363 8.816 17.233 1.00 0.00 C

ATOM 925 CD1 ILE A 59 -3.494 11.629 17.221 1.00 0.00 C

ATOM 926 H ILE A 59 -1.487 10.978 20.699 1.00 0.00 H

ATOM 927 HA ILE A 59 -3.846 9.863 19.252 1.00 0.00 H

ATOM 928 HB ILE A 59 -0.897 9.739 18.488 1.00 0.00 H

ATOM 929 1HG1 ILE A 59 -1.746 12.018 18.361 1.00 0.00 H

ATOM 930 2HG1 ILE A 59 -1.440 11.358 16.762 1.00 0.00 H

ATOM 931 1HG2 ILE A 59 -1.755 8.912 16.334 1.00 0.00 H

ATOM 932 2HG2 ILE A 59 -2.216 7.827 17.665 1.00 0.00 H

ATOM 933 3HG2 ILE A 59 -3.414 8.948 16.976 1.00 0.00 H

ATOM 934 1HD1 ILE A 59 -3.509 12.635 16.802 1.00 0.00 H

ATOM 935 2HD1 ILE A 59 -3.840 10.918 16.470 1.00 0.00 H

ATOM 936 3HD1 ILE A 59 -4.150 11.588 18.089 1.00 0.00 H

ATOM 937 N ASP A 60 -3.629 7.551 20.067 1.00 0.00 N

ATOM 938 CA ASP A 60 -3.542 6.176 20.545 1.00 0.00 C

ATOM 939 C ASP A 60 -2.518 5.379 19.748 1.00 0.00 C

ATOM 940 O ASP A 60 -2.490 5.440 18.518 1.00 0.00 O

ATOM 941 CB ASP A 60 -4.908 5.491 20.462 1.00 0.00 C

ATOM 942 CG ASP A 60 -4.898 4.077 21.027 1.00 0.00 C

ATOM 943 OD1 ASP A 60 -5.321 3.903 22.145 1.00 0.00 O

ATOM 944 OD2 ASP A 60 -4.467 3.186 20.336 1.00 0.00 O

ATOM 945 H ASP A 60 -4.497 7.878 19.668 1.00 0.00 H

ATOM 946 HA ASP A 60 -3.224 6.192 21.588 1.00 0.00 H

ATOM 947 1HB ASP A 60 -5.645 6.080 21.008 1.00 0.00 H

ATOM 948 2HB ASP A 60 -5.230 5.448 19.421 1.00 0.00 H

ATOM 949 N TYR A 61 -1.676 4.632 20.454 1.00 0.00 N

ATOM 950 CA TYR A 61 -0.616 3.860 19.817 1.00 0.00 C

ATOM 951 C TYR A 61 -1.181 2.902 18.777 1.00 0.00 C

ATOM 952 O TYR A 61 -0.740 2.886 17.628 1.00 0.00 O

ATOM 953 CB TYR A 61 0.191 3.091 20.865 1.00 0.00 C

ATOM 954 CG TYR A 61 1.189 2.120 20.275 1.00 0.00 C

ATOM 955 CD1 TYR A 61 2.235 2.589 19.494 1.00 0.00 C

ATOM 956 CD2 TYR A 61 1.058 0.760 20.514 1.00 0.00 C

ATOM 957 CE1 TYR A 61 3.147 1.702 18.956 1.00 0.00 C

ATOM 958 CE2 TYR A 61 1.970 -0.127 19.976 1.00 0.00 C

ATOM 959 CZ TYR A 61 3.011 0.340 19.199 1.00 0.00 C

ATOM 960 OH TYR A 61 3.919 -0.544 18.662 1.00 0.00 O

ATOM 961 H TYR A 61 -1.774 4.597 21.459 1.00 0.00 H

ATOM 962 HA TYR A 61 0.052 4.551 19.301 1.00 0.00 H

ATOM 963 1HB TYR A 61 0.734 3.796 21.496 1.00 0.00 H

ATOM 964 2HB TYR A 61 -0.489 2.532 21.508 1.00 0.00 H

ATOM 965 HD1 TYR A 61 2.338 3.658 19.306 1.00 0.00 H

ATOM 966 HD2 TYR A 61 0.235 0.391 21.127 1.00 0.00 H

ATOM 967 HE1 TYR A 61 3.969 2.071 18.342 1.00 0.00 H

ATOM 968 HE2 TYR A 61 1.867 -1.196 20.163 1.00 0.00 H

ATOM 969 HH TYR A 61 3.714 -1.433 18.962 1.00 0.00 H

ATOM 970 N THR A 62 -2.160 2.102 19.187 1.00 0.00 N

ATOM 971 CA THR A 62 -2.750 1.100 18.307 1.00 0.00 C

ATOM 972 C THR A 62 -3.329 1.740 17.052 1.00 0.00 C

ATOM 973 O THR A 62 -3.155 1.229 15.946 1.00 0.00 O

ATOM 974 CB THR A 62 -3.848 0.302 19.034 1.00 0.00 C

ATOM 975 OG1 THR A 62 -3.278 -0.392 20.152 1.00 0.00 O

ATOM 976 CG2 THR A 62 -4.488 -0.705 18.091 1.00 0.00 C

ATOM 977 H THR A 62 -2.505 2.191 20.132 1.00 0.00 H

ATOM 978 HA THR A 62 -1.967 0.406 18.000 1.00 0.00 H

ATOM 979 HB THR A 62 -4.613 0.986 19.400 1.00 0.00 H

ATOM 980 HG1 THR A 62 -3.442 0.108 20.955 1.00 0.00 H

ATOM 981 1HG2 THR A 62 -5.262 -1.260 18.622 1.00 0.00 H

ATOM 982 2HG2 THR A 62 -4.933 -0.180 17.246 1.00 0.00 H

ATOM 983 3HG2 THR A 62 -3.730 -1.398 17.729 1.00 0.00 H

ATOM 984 N GLU A 63 -4.018 2.862 17.231 1.00 0.00 N

ATOM 985 CA GLU A 63 -4.608 3.585 16.111 1.00 0.00 C

ATOM 986 C GLU A 63 -3.537 4.267 15.269 1.00 0.00 C

ATOM 987 O GLU A 63 -3.660 4.360 14.048 1.00 0.00 O

ATOM 988 CB GLU A 63 -5.612 4.624 16.616 1.00 0.00 C

ATOM 989 CG GLU A 63 -6.854 4.033 17.268 1.00 0.00 C

ATOM 990 CD GLU A 63 -7.670 3.196 16.322 1.00 0.00 C

ATOM 991 OE1 GLU A 63 -7.862 3.613 15.205 1.00 0.00 O

ATOM 992 OE2 GLU A 63 -8.102 2.139 16.717 1.00 0.00 O

ATOM 993 H GLU A 63 -4.136 3.224 18.166 1.00 0.00 H

ATOM 994 HA GLU A 63 -5.134 2.871 15.477 1.00 0.00 H

ATOM 995 1HB GLU A 63 -5.128 5.273 17.345 1.00 0.00 H

ATOM 996 2HB GLU A 63 -5.937 5.250 15.785 1.00 0.00 H

ATOM 997 1HG GLU A 63 -6.549 3.414 18.112 1.00 0.00 H

ATOM 998 2HG GLU A 63 -7.470 4.844 17.653 1.00 0.00 H

ATOM 999 N PHE A 64 -2.487 4.743 15.929 1.00 0.00 N

ATOM 1000 CA PHE A 64 -1.362 5.360 15.237 1.00 0.00 C

ATOM 1001 C PHE A 64 -0.735 4.398 14.237 1.00 0.00 C

ATOM 1002 O PHE A 64 -0.547 4.737 13.069 1.00 0.00 O

ATOM 1003 CB PHE A 64 -0.305 5.822 16.241 1.00 0.00 C

ATOM 1004 CG PHE A 64 0.939 6.372 15.602 1.00 0.00 C

ATOM 1005 CD1 PHE A 64 1.007 7.703 15.218 1.00 0.00 C

ATOM 1006 CD2 PHE A 64 2.041 5.560 15.384 1.00 0.00 C

ATOM 1007 CE1 PHE A 64 2.151 8.210 14.630 1.00 0.00 C

ATOM 1008 CE2 PHE A 64 3.186 6.065 14.798 1.00 0.00 C

ATOM 1009 CZ PHE A 64 3.240 7.391 14.420 1.00 0.00 C

ATOM 1010 H PHE A 64 -2.467 4.674 16.937 1.00 0.00 H

ATOM 1011 HA PHE A 64 -1.728 6.231 14.691 1.00 0.00 H

ATOM 1012 1HB PHE A 64 -0.726 6.593 16.885 1.00 0.00 H

ATOM 1013 2HB PHE A 64 -0.018 4.985 16.878 1.00 0.00 H

ATOM 1014 HD1 PHE A 64 0.146 8.350 15.385 1.00 0.00 H

ATOM 1015 HD2 PHE A 64 1.999 4.512 15.682 1.00 0.00 H

ATOM 1016 HE1 PHE A 64 2.191 9.257 14.332 1.00 0.00 H

ATOM 1017 HE2 PHE A 64 4.046 5.416 14.632 1.00 0.00 H

ATOM 1018 HZ PHE A 64 4.141 7.790 13.956 1.00 0.00 H

ATOM 1019 N ILE A 65 -0.413 3.196 14.703 1.00 0.00 N

ATOM 1020 CA ILE A 65 0.251 2.203 13.867 1.00 0.00 C

ATOM 1021 C ILE A 65 -0.720 1.585 12.869 1.00 0.00 C

ATOM 1022 O ILE A 65 -0.313 1.090 11.818 1.00 0.00 O

ATOM 1023 CB ILE A 65 0.880 1.092 14.728 1.00 0.00 C

ATOM 1024 CG1 ILE A 65 -0.210 0.296 15.450 1.00 0.00 C

ATOM 1025 CG2 ILE A 65 1.862 1.684 15.727 1.00 0.00 C

ATOM 1026 CD1 ILE A 65 0.312 -0.903 16.209 1.00 0.00 C

ATOM 1027 H ILE A 65 -0.634 2.964 15.661 1.00 0.00 H

ATOM 1028 HA ILE A 65 1.045 2.697 13.308 1.00 0.00 H

ATOM 1029 HB ILE A 65 1.409 0.389 14.085 1.00 0.00 H

ATOM 1030 1HG1 ILE A 65 -0.729 0.945 16.154 1.00 0.00 H

ATOM 1031 2HG1 ILE A 65 -0.946 -0.055 14.726 1.00 0.00 H

ATOM 1032 1HG2 ILE A 65 2.296 0.886 16.327 1.00 0.00 H

ATOM 1033 2HG2 ILE A 65 2.653 2.208 15.192 1.00 0.00 H

ATOM 1034 3HG2 ILE A 65 1.339 2.385 16.378 1.00 0.00 H

ATOM 1035 1HD1 ILE A 65 -0.519 -1.417 16.694 1.00 0.00 H

ATOM 1036 2HD1 ILE A 65 0.806 -1.586 15.517 1.00 0.00 H

ATOM 1037 3HD1 ILE A 65 1.024 -0.574 16.965 1.00 0.00 H

ATOM 1038 N ALA A 66 -2.005 1.617 13.204 1.00 0.00 N

ATOM 1039 CA ALA A 66 -3.049 1.198 12.277 1.00 0.00 C

ATOM 1040 C ALA A 66 -3.143 2.145 11.088 1.00 0.00 C

ATOM 1041 O ALA A 66 -3.271 1.710 9.944 1.00 0.00 O

ATOM 1042 CB ALA A 66 -4.390 1.113 12.992 1.00 0.00 C

ATOM 1043 H ALA A 66 -2.265 1.940 14.125 1.00 0.00 H

ATOM 1044 HA ALA A 66 -2.788 0.210 11.897 1.00 0.00 H

ATOM 1045 1HB ALA A 66 -5.159 0.799 12.287 1.00 0.00 H

ATOM 1046 2HB ALA A 66 -4.324 0.388 13.804 1.00 0.00 H

ATOM 1047 3HB ALA A 66 -4.648 2.089 13.399 1.00 0.00 H

ATOM 1048 N ALA A 67 -3.079 3.443 11.366 1.00 0.00 N

ATOM 1049 CA ALA A 67 -3.054 4.451 10.313 1.00 0.00 C

ATOM 1050 C ALA A 67 -1.771 4.364 9.497 1.00 0.00 C

ATOM 1051 O ALA A 67 -1.788 4.525 8.276 1.00 0.00 O

ATOM 1052 CB ALA A 67 -3.208 5.843 10.909 1.00 0.00 C

ATOM 1053 H ALA A 67 -3.046 3.739 12.331 1.00 0.00 H

ATOM 1054 HA ALA A 67 -3.891 4.260 9.641 1.00 0.00 H

ATOM 1055 1HB ALA A 67 -3.188 6.585 10.110 1.00 0.00 H

ATOM 1056 2HB ALA A 67 -4.158 5.908 11.440 1.00 0.00 H

ATOM 1057 3HB ALA A 67 -2.391 6.034 11.602 1.00 0.00 H

ATOM 1058 N ALA A 68 -0.659 4.109 10.177 1.00 0.00 N

ATOM 1059 CA ALA A 68 0.630 3.963 9.512 1.00 0.00 C

ATOM 1060 C ALA A 68 0.640 2.751 8.590 1.00 0.00 C

ATOM 1061 O ALA A 68 1.198 2.797 7.493 1.00 0.00 O

ATOM 1062 CB ALA A 68 1.748 3.854 10.540 1.00 0.00 C

ATOM 1063 H ALA A 68 -0.708 4.013 11.182 1.00 0.00 H

ATOM 1064 HA ALA A 68 0.798 4.849 8.901 1.00 0.00 H

ATOM 1065 1HB ALA A 68 2.704 3.746 10.028 1.00 0.00 H

ATOM 1066 2HB ALA A 68 1.765 4.754 11.154 1.00 0.00 H

ATOM 1067 3HB ALA A 68 1.576 2.986 11.174 1.00 0.00 H

ATOM 1068 N LEU A 69 0.020 1.666 9.040 1.00 0.00 N

ATOM 1069 CA LEU A 69 -0.021 0.430 8.267 1.00 0.00 C

ATOM 1070 C LEU A 69 -0.708 0.642 6.925 1.00 0.00 C

ATOM 1071 O LEU A 69 -1.866 1.056 6.867 1.00 0.00 O

ATOM 1072 CB LEU A 69 -0.752 -0.664 9.056 1.00 0.00 C

ATOM 1073 CG LEU A 69 -0.778 -2.051 8.401 1.00 0.00 C

ATOM 1074 CD1 LEU A 69 0.639 -2.604 8.324 1.00 0.00 C

ATOM 1075 CD2 LEU A 69 -1.683 -2.975 9.203 1.00 0.00 C

ATOM 1076 H LEU A 69 -0.436 1.698 9.941 1.00 0.00 H

ATOM 1077 HA LEU A 69 1.002 0.104 8.082 1.00 0.00 H

ATOM 1078 1HB LEU A 69 -0.277 -0.767 10.030 1.00 0.00 H

ATOM 1079 2HB LEU A 69 -1.785 -0.351 9.209 1.00 0.00 H

ATOM 1080 HG LEU A 69 -1.158 -1.966 7.382 1.00 0.00 H

ATOM 1081 1HD1 LEU A 69 0.620 -3.590 7.859 1.00 0.00 H

ATOM 1082 2HD1 LEU A 69 1.259 -1.934 7.729 1.00 0.00 H

ATOM 1083 3HD1 LEU A 69 1.052 -2.685 9.329 1.00 0.00 H

ATOM 1084 1HD2 LEU A 69 -1.702 -3.960 8.737 1.00 0.00 H

ATOM 1085 2HD2 LEU A 69 -1.303 -3.062 10.221 1.00 0.00 H

ATOM 1086 3HD2 LEU A 69 -2.693 -2.564 9.226 1.00 0.00 H

ATOM 1087 N ASP A 70 0.013 0.356 5.845 1.00 0.00 N

ATOM 1088 CA ASP A 70 -0.530 0.504 4.500 1.00 0.00 C

ATOM 1089 C ASP A 70 0.374 -0.154 3.466 1.00 0.00 C

ATOM 1090 O ASP A 70 0.330 -1.341 3.300 1.00 0.00 O

ATOM 1091 OXT ASP A 70 1.129 0.516 2.817 1.00 0.00 O

ATOM 1092 CB ASP A 70 -0.716 1.984 4.157 1.00 0.00 C

ATOM 1093 CG ASP A 70 -1.444 2.199 2.837 1.00 0.00 C

ATOM 1094 OD1 ASP A 70 -1.278 1.395 1.951 1.00 0.00 O

ATOM 1095 OD2 ASP A 70 -2.160 3.166 2.727 1.00 0.00 O

ATOM 1096 H ASP A 70 0.961 0.028 5.960 1.00 0.00 H

ATOM 1097 HA ASP A 70 -1.503 0.014 4.464 1.00 0.00 H

ATOM 1098 1HB ASP A 70 -1.280 2.474 4.951 1.00 0.00 H

ATOM 1099 2HB ASP A 70 0.259 2.470 4.101 1.00 0.00 H

TER
