# Supplementary material for: Biophysical characterization of the calmodulin-like domain of Plasmodium falciparum calcium dependent protein kinase 3
Source: PLoS One. 2017 Jul 26;12(7):e0181721. doi: 10.1371/journal.pone.0181721 (PMC5528832; doi:10.1371/journal.pone.0181721)
Supplement: S2 Table — (DOCX) [file pone.0181721.s007.docx]

**S2 Table. Coordinates for the selected CS-Rosetta model of *pf*CDPK3 CLD N-lobe^Ca^.**

ATOM 1 N GLY A 1 0.000 0.000 0.000 1.00 0.00 N

ATOM 2 CA GLY A 1 1.458 0.000 0.000 1.00 0.00 C

ATOM 3 C GLY A 1 2.009 1.420 0.000 1.00 0.00 C

ATOM 4 O GLY A 1 1.298 2.372 -0.321 1.00 0.00 O

ATOM 5 1H GLY A 1 -0.334 -0.943 -0.000 1.00 0.00 H

ATOM 6 2H GLY A 1 -0.334 0.471 0.816 1.00 0.00 H

ATOM 7 3H GLY A 1 -0.334 0.471 -0.816 1.00 0.00 H

ATOM 8 1HA GLY A 1 1.822 -0.535 0.877 1.00 0.00 H

ATOM 9 2HA GLY A 1 1.822 -0.535 -0.876 1.00 0.00 H

ATOM 10 N ASN A 2 3.280 1.556 0.362 1.00 0.00 N

ATOM 11 CA ASN A 2 3.924 2.863 0.422 1.00 0.00 C

ATOM 12 C ASN A 2 4.651 3.180 -0.878 1.00 0.00 C

ATOM 13 O ASN A 2 5.056 2.276 -1.610 1.00 0.00 O

ATOM 14 CB ASN A 2 4.879 2.934 1.599 1.00 0.00 C

ATOM 15 CG ASN A 2 4.170 2.881 2.924 1.00 0.00 C

ATOM 16 OD1 ASN A 2 3.314 3.723 3.218 1.00 0.00 O

ATOM 17 ND2 ASN A 2 4.509 1.907 3.729 1.00 0.00 N

ATOM 18 H ASN A 2 3.814 0.733 0.602 1.00 0.00 H

ATOM 19 HA ASN A 2 3.152 3.623 0.555 1.00 0.00 H

ATOM 20 1HB ASN A 2 5.586 2.105 1.545 1.00 0.00 H

ATOM 21 2HB ASN A 2 5.455 3.858 1.546 1.00 0.00 H

ATOM 22 1HD2 ASN A 2 4.069 1.821 4.624 1.00 0.00 H

ATOM 23 2HD2 ASN A 2 5.206 1.247 3.450 1.00 0.00 H

ATOM 24 N ASP A 3 4.813 4.468 -1.161 1.00 0.00 N

ATOM 25 CA ASP A 3 5.359 4.908 -2.439 1.00 0.00 C

ATOM 26 C ASP A 3 6.879 4.815 -2.451 1.00 0.00 C

ATOM 27 O ASP A 3 7.574 5.821 -2.308 1.00 0.00 O

ATOM 28 CB ASP A 3 4.928 6.346 -2.739 1.00 0.00 C

ATOM 29 CG ASP A 3 5.323 6.804 -4.137 1.00 0.00 C

ATOM 30 OD1 ASP A 3 5.816 5.996 -4.888 1.00 0.00 O

ATOM 31 OD2 ASP A 3 5.127 7.956 -4.440 1.00 0.00 O

ATOM 32 H ASP A 3 4.552 5.159 -0.473 1.00 0.00 H

ATOM 33 HA ASP A 3 4.971 4.258 -3.224 1.00 0.00 H

ATOM 34 1HB ASP A 3 3.846 6.431 -2.637 1.00 0.00 H

ATOM 35 2HB ASP A 3 5.379 7.021 -2.011 1.00 0.00 H

ATOM 36 N TYR A 4 7.391 3.600 -2.622 1.00 0.00 N

ATOM 37 CA TYR A 4 8.825 3.388 -2.776 1.00 0.00 C

ATOM 38 C TYR A 4 9.126 2.520 -3.991 1.00 0.00 C

ATOM 39 O TYR A 4 8.338 1.646 -4.352 1.00 0.00 O

ATOM 40 CB TYR A 4 9.411 2.753 -1.513 1.00 0.00 C

ATOM 41 CG TYR A 4 9.358 3.652 -0.298 1.00 0.00 C

ATOM 42 CD1 TYR A 4 8.296 3.557 0.590 1.00 0.00 C

ATOM 43 CD2 TYR A 4 10.371 4.571 -0.071 1.00 0.00 C

ATOM 44 CE1 TYR A 4 8.248 4.378 1.700 1.00 0.00 C

ATOM 45 CE2 TYR A 4 10.323 5.392 1.039 1.00 0.00 C

ATOM 46 CZ TYR A 4 9.267 5.298 1.922 1.00 0.00 C

ATOM 47 OH TYR A 4 9.219 6.115 3.028 1.00 0.00 O

ATOM 48 H TYR A 4 6.770 2.804 -2.646 1.00 0.00 H

ATOM 49 HA TYR A 4 9.303 4.355 -2.934 1.00 0.00 H

ATOM 50 1HB TYR A 4 8.868 1.835 -1.281 1.00 0.00 H

ATOM 51 2HB TYR A 4 10.451 2.482 -1.691 1.00 0.00 H

ATOM 52 HD1 TYR A 4 7.500 2.834 0.412 1.00 0.00 H

ATOM 53 HD2 TYR A 4 11.205 4.647 -0.768 1.00 0.00 H

ATOM 54 HE1 TYR A 4 7.414 4.304 2.397 1.00 0.00 H

ATOM 55 HE2 TYR A 4 11.120 6.116 1.217 1.00 0.00 H

ATOM 56 HH TYR A 4 8.997 5.593 3.802 1.00 0.00 H

ATOM 57 N ASP A 5 10.270 2.766 -4.620 1.00 0.00 N

ATOM 58 CA ASP A 5 10.707 1.967 -5.758 1.00 0.00 C

ATOM 59 C ASP A 5 11.087 0.556 -5.326 1.00 0.00 C

ATOM 60 O ASP A 5 11.485 0.332 -4.183 1.00 0.00 O

ATOM 61 CB ASP A 5 11.897 2.631 -6.455 1.00 0.00 C

ATOM 62 CG ASP A 5 11.522 3.928 -7.159 1.00 0.00 C

ATOM 63 OD1 ASP A 5 10.356 4.138 -7.395 1.00 0.00 O

ATOM 64 OD2 ASP A 5 12.406 4.697 -7.453 1.00 0.00 O

ATOM 65 H ASP A 5 10.853 3.527 -4.300 1.00 0.00 H

ATOM 66 HA ASP A 5 9.883 1.896 -6.468 1.00 0.00 H

ATOM 67 1HB ASP A 5 12.676 2.844 -5.722 1.00 0.00 H

ATOM 68 2HB ASP A 5 12.318 1.944 -7.190 1.00 0.00 H

ATOM 69 N VAL A 6 10.961 -0.393 -6.248 1.00 0.00 N

ATOM 70 CA VAL A 6 11.251 -1.791 -5.952 1.00 0.00 C

ATOM 71 C VAL A 6 12.677 -1.963 -5.445 1.00 0.00 C

ATOM 72 O VAL A 6 12.918 -2.682 -4.475 1.00 0.00 O

ATOM 73 CB VAL A 6 11.049 -2.656 -7.210 1.00 0.00 C

ATOM 74 CG1 VAL A 6 11.566 -4.068 -6.975 1.00 0.00 C

ATOM 75 CG2 VAL A 6 9.577 -2.678 -7.593 1.00 0.00 C

ATOM 76 H VAL A 6 10.657 -0.140 -7.177 1.00 0.00 H

ATOM 77 HA VAL A 6 10.561 -2.131 -5.179 1.00 0.00 H

ATOM 78 HB VAL A 6 11.632 -2.233 -8.028 1.00 0.00 H

ATOM 79 1HG1 VAL A 6 11.416 -4.665 -7.874 1.00 0.00 H

ATOM 80 2HG1 VAL A 6 12.629 -4.031 -6.738 1.00 0.00 H

ATOM 81 3HG1 VAL A 6 11.024 -4.520 -6.145 1.00 0.00 H

ATOM 82 1HG2 VAL A 6 9.442 -3.291 -8.484 1.00 0.00 H

ATOM 83 2HG2 VAL A 6 8.994 -3.096 -6.772 1.00 0.00 H

ATOM 84 3HG2 VAL A 6 9.239 -1.662 -7.797 1.00 0.00 H

ATOM 85 N GLU A 7 13.619 -1.299 -6.106 1.00 0.00 N

ATOM 86 CA GLU A 7 15.026 -1.392 -5.735 1.00 0.00 C

ATOM 87 C GLU A 7 15.285 -0.732 -4.387 1.00 0.00 C

ATOM 88 O GLU A 7 16.146 -1.171 -3.625 1.00 0.00 O

ATOM 89 CB GLU A 7 15.904 -0.746 -6.808 1.00 0.00 C

ATOM 90 CG GLU A 7 15.895 -1.469 -8.148 1.00 0.00 C

ATOM 91 CD GLU A 7 16.378 -2.889 -8.049 1.00 0.00 C

ATOM 92 OE1 GLU A 7 17.408 -3.107 -7.457 1.00 0.00 O

ATOM 93 OE2 GLU A 7 15.715 -3.758 -8.566 1.00 0.00 O

ATOM 94 H GLU A 7 13.353 -0.714 -6.885 1.00 0.00 H

ATOM 95 HA GLU A 7 15.293 -2.446 -5.653 1.00 0.00 H

ATOM 96 1HB GLU A 7 15.575 0.279 -6.979 1.00 0.00 H

ATOM 97 2HB GLU A 7 16.936 -0.704 -6.458 1.00 0.00 H

ATOM 98 1HG GLU A 7 14.879 -1.469 -8.542 1.00 0.00 H

ATOM 99 2HG GLU A 7 16.527 -0.923 -8.847 1.00 0.00 H

ATOM 100 N LYS A 8 14.535 0.327 -4.098 1.00 0.00 N

ATOM 101 CA LYS A 8 14.611 0.989 -2.801 1.00 0.00 C

ATOM 102 C LYS A 8 14.062 0.097 -1.695 1.00 0.00 C

ATOM 103 O LYS A 8 14.551 0.121 -0.566 1.00 0.00 O

ATOM 104 CB LYS A 8 13.853 2.316 -2.833 1.00 0.00 C

ATOM 105 CG LYS A 8 14.515 3.400 -3.674 1.00 0.00 C

ATOM 106 CD LYS A 8 13.688 4.676 -3.681 1.00 0.00 C

ATOM 107 CE LYS A 8 14.335 5.752 -4.541 1.00 0.00 C

ATOM 108 NZ LYS A 8 13.510 6.989 -4.601 1.00 0.00 N

ATOM 109 H LYS A 8 13.896 0.682 -4.795 1.00 0.00 H

ATOM 110 HA LYS A 8 15.659 1.191 -2.577 1.00 0.00 H

ATOM 111 1HB LYS A 8 12.850 2.153 -3.227 1.00 0.00 H

ATOM 112 2HB LYS A 8 13.747 2.699 -1.817 1.00 0.00 H

ATOM 113 1HG LYS A 8 15.504 3.620 -3.270 1.00 0.00 H

ATOM 114 2HG LYS A 8 14.630 3.046 -4.697 1.00 0.00 H

ATOM 115 1HD LYS A 8 12.691 4.463 -4.070 1.00 0.00 H

ATOM 116 2HD LYS A 8 13.588 5.051 -2.662 1.00 0.00 H

ATOM 117 1HE LYS A 8 15.314 6.002 -4.135 1.00 0.00 H

ATOM 118 2HE LYS A 8 14.473 5.373 -5.554 1.00 0.00 H

ATOM 119 1HZ LYS A 8 13.973 7.675 -5.180 1.00 0.00 H

ATOM 120 2HZ LYS A 8 12.605 6.772 -4.994 1.00 0.00 H

ATOM 121 3HZ LYS A 8 13.392 7.360 -3.669 1.00 0.00 H

ATOM 122 N LEU A 9 13.043 -0.688 -2.026 1.00 0.00 N

ATOM 123 CA LEU A 9 12.483 -1.653 -1.088 1.00 0.00 C

ATOM 124 C LEU A 9 13.455 -2.797 -0.829 1.00 0.00 C

ATOM 125 O LEU A 9 13.545 -3.308 0.288 1.00 0.00 O

ATOM 126 CB LEU A 9 11.159 -2.210 -1.627 1.00 0.00 C

ATOM 127 CG LEU A 9 9.991 -1.218 -1.677 1.00 0.00 C

ATOM 128 CD1 LEU A 9 8.842 -1.825 -2.470 1.00 0.00 C

ATOM 129 CD2 LEU A 9 9.556 -0.873 -0.260 1.00 0.00 C

ATOM 130 H LEU A 9 12.645 -0.614 -2.951 1.00 0.00 H

ATOM 131 HA LEU A 9 12.291 -1.146 -0.143 1.00 0.00 H

ATOM 132 1HB LEU A 9 11.324 -2.579 -2.638 1.00 0.00 H

ATOM 133 2HB LEU A 9 10.855 -3.049 -1.001 1.00 0.00 H

ATOM 134 HG LEU A 9 10.307 -0.309 -2.190 1.00 0.00 H

ATOM 135 1HD1 LEU A 9 8.012 -1.119 -2.506 1.00 0.00 H

ATOM 136 2HD1 LEU A 9 9.175 -2.043 -3.485 1.00 0.00 H

ATOM 137 3HD1 LEU A 9 8.514 -2.746 -1.989 1.00 0.00 H

ATOM 138 1HD2 LEU A 9 8.726 -0.167 -0.296 1.00 0.00 H

ATOM 139 2HD2 LEU A 9 9.238 -1.781 0.254 1.00 0.00 H

ATOM 140 3HD2 LEU A 9 10.391 -0.425 0.278 1.00 0.00 H

ATOM 141 N LYS A 10 14.181 -3.196 -1.868 1.00 0.00 N

ATOM 142 CA LYS A 10 15.228 -4.201 -1.729 1.00 0.00 C

ATOM 143 C LYS A 10 16.297 -3.751 -0.741 1.00 0.00 C

ATOM 144 O LYS A 10 16.700 -4.510 0.140 1.00 0.00 O

ATOM 145 CB LYS A 10 15.862 -4.504 -3.088 1.00 0.00 C

ATOM 146 CG LYS A 10 14.964 -5.279 -4.043 1.00 0.00 C

ATOM 147 CD LYS A 10 15.650 -5.514 -5.380 1.00 0.00 C

ATOM 148 CE LYS A 10 14.750 -6.280 -6.338 1.00 0.00 C

ATOM 149 NZ LYS A 10 15.372 -6.436 -7.681 1.00 0.00 N

ATOM 150 H LYS A 10 14.003 -2.793 -2.776 1.00 0.00 H

ATOM 151 HA LYS A 10 14.780 -5.117 -1.342 1.00 0.00 H

ATOM 152 1HB LYS A 10 16.142 -3.570 -3.576 1.00 0.00 H

ATOM 153 2HB LYS A 10 16.774 -5.084 -2.943 1.00 0.00 H

ATOM 154 1HG LYS A 10 14.708 -6.243 -3.601 1.00 0.00 H

ATOM 155 2HG LYS A 10 14.043 -4.720 -4.210 1.00 0.00 H

ATOM 156 1HD LYS A 10 15.912 -4.555 -5.828 1.00 0.00 H

ATOM 157 2HD LYS A 10 16.566 -6.084 -5.223 1.00 0.00 H

ATOM 158 1HE LYS A 10 14.543 -7.268 -5.930 1.00 0.00 H

ATOM 159 2HE LYS A 10 13.804 -5.751 -6.449 1.00 0.00 H

ATOM 160 1HZ LYS A 10 14.745 -6.947 -8.286 1.00 0.00 H

ATOM 161 2HZ LYS A 10 15.552 -5.524 -8.077 1.00 0.00 H

ATOM 162 3HZ LYS A 10 16.242 -6.942 -7.593 1.00 0.00 H

ATOM 163 N SER A 11 16.752 -2.512 -0.894 1.00 0.00 N

ATOM 164 CA SER A 11 17.738 -1.941 0.016 1.00 0.00 C

ATOM 165 C SER A 11 17.184 -1.835 1.431 1.00 0.00 C

ATOM 166 O SER A 11 17.818 -2.273 2.391 1.00 0.00 O

ATOM 167 CB SER A 11 18.167 -0.571 -0.472 1.00 0.00 C

ATOM 168 OG SER A 11 19.103 0.002 0.399 1.00 0.00 O

ATOM 169 H SER A 11 16.407 -1.952 -1.660 1.00 0.00 H

ATOM 170 HA SER A 11 18.610 -2.596 0.038 1.00 0.00 H

ATOM 171 1HB SER A 11 18.600 -0.660 -1.468 1.00 0.00 H

ATOM 172 2HB SER A 11 17.295 0.077 -0.551 1.00 0.00 H

ATOM 173 HG SER A 11 18.674 0.773 0.778 1.00 0.00 H

ATOM 174 N THR A 12 15.997 -1.250 1.555 1.00 0.00 N

ATOM 175 CA THR A 12 15.367 -1.061 2.856 1.00 0.00 C

ATOM 176 C THR A 12 15.193 -2.389 3.582 1.00 0.00 C

ATOM 177 O THR A 12 15.412 -2.480 4.790 1.00 0.00 O

ATOM 178 CB THR A 12 14.001 -0.365 2.715 1.00 0.00 C

ATOM 179 OG1 THR A 12 14.174 0.912 2.089 1.00 0.00 O

ATOM 180 CG2 THR A 12 13.356 -0.174 4.079 1.00 0.00 C

ATOM 181 H THR A 12 15.519 -0.928 0.725 1.00 0.00 H

ATOM 182 HA THR A 12 16.014 -0.428 3.464 1.00 0.00 H

ATOM 183 HB THR A 12 13.345 -0.974 2.092 1.00 0.00 H

ATOM 184 HG1 THR A 12 14.315 0.790 1.147 1.00 0.00 H

ATOM 185 1HG2 THR A 12 12.392 0.319 3.960 1.00 0.00 H

ATOM 186 2HG2 THR A 12 13.213 -1.145 4.553 1.00 0.00 H

ATOM 187 3HG2 THR A 12 14.003 0.442 4.703 1.00 0.00 H

ATOM 188 N PHE A 13 14.799 -3.417 2.838 1.00 0.00 N

ATOM 189 CA PHE A 13 14.677 -4.760 3.391 1.00 0.00 C

ATOM 190 C PHE A 13 15.975 -5.204 4.052 1.00 0.00 C

ATOM 191 O PHE A 13 15.976 -5.662 5.195 1.00 0.00 O

ATOM 192 CB PHE A 13 14.291 -5.756 2.295 1.00 0.00 C

ATOM 193 CG PHE A 13 14.258 -7.184 2.758 1.00 0.00 C

ATOM 194 CD1 PHE A 13 13.089 -7.739 3.259 1.00 0.00 C

ATOM 195 CD2 PHE A 13 15.394 -7.976 2.696 1.00 0.00 C

ATOM 196 CE1 PHE A 13 13.057 -9.053 3.686 1.00 0.00 C

ATOM 197 CE2 PHE A 13 15.365 -9.290 3.120 1.00 0.00 C

ATOM 198 CZ PHE A 13 14.195 -9.829 3.616 1.00 0.00 C

ATOM 199 H PHE A 13 14.578 -3.263 1.864 1.00 0.00 H

ATOM 200 HA PHE A 13 13.892 -4.753 4.148 1.00 0.00 H

ATOM 201 1HB PHE A 13 13.306 -5.502 1.904 1.00 0.00 H

ATOM 202 2HB PHE A 13 14.999 -5.682 1.471 1.00 0.00 H

ATOM 203 HD1 PHE A 13 12.189 -7.126 3.313 1.00 0.00 H

ATOM 204 HD2 PHE A 13 16.319 -7.550 2.304 1.00 0.00 H

ATOM 205 HE1 PHE A 13 12.133 -9.476 4.077 1.00 0.00 H

ATOM 206 HE2 PHE A 13 16.266 -9.902 3.065 1.00 0.00 H

ATOM 207 HZ PHE A 13 14.171 -10.864 3.954 1.00 0.00 H

ATOM 208 N LEU A 14 17.080 -5.065 3.328 1.00 0.00 N

ATOM 209 CA LEU A 14 18.380 -5.506 3.820 1.00 0.00 C

ATOM 210 C LEU A 14 18.807 -4.704 5.042 1.00 0.00 C

ATOM 211 O LEU A 14 19.432 -5.237 5.959 1.00 0.00 O

ATOM 212 CB LEU A 14 19.437 -5.373 2.717 1.00 0.00 C

ATOM 213 CG LEU A 14 19.274 -6.319 1.521 1.00 0.00 C

ATOM 214 CD1 LEU A 14 20.289 -5.958 0.444 1.00 0.00 C

ATOM 215 CD2 LEU A 14 19.456 -7.757 1.982 1.00 0.00 C

ATOM 216 H LEU A 14 17.018 -4.643 2.412 1.00 0.00 H

ATOM 217 HA LEU A 14 18.303 -6.554 4.107 1.00 0.00 H

ATOM 218 1HB LEU A 14 19.416 -4.353 2.337 1.00 0.00 H

ATOM 219 2HB LEU A 14 20.419 -5.556 3.153 1.00 0.00 H

ATOM 220 HG LEU A 14 18.278 -6.198 1.094 1.00 0.00 H

ATOM 221 1HD1 LEU A 14 20.172 -6.630 -0.406 1.00 0.00 H

ATOM 222 2HD1 LEU A 14 20.123 -4.930 0.119 1.00 0.00 H

ATOM 223 3HD1 LEU A 14 21.296 -6.053 0.847 1.00 0.00 H

ATOM 224 1HD2 LEU A 14 19.339 -8.430 1.132 1.00 0.00 H

ATOM 225 2HD2 LEU A 14 20.452 -7.880 2.408 1.00 0.00 H

ATOM 226 3HD2 LEU A 14 18.707 -7.994 2.738 1.00 0.00 H

ATOM 227 N VAL A 15 18.467 -3.419 5.049 1.00 0.00 N

ATOM 228 CA VAL A 15 18.779 -2.550 6.177 1.00 0.00 C

ATOM 229 C VAL A 15 18.029 -2.983 7.430 1.00 0.00 C

ATOM 230 O VAL A 15 18.606 -3.062 8.514 1.00 0.00 O

ATOM 231 CB VAL A 15 18.416 -1.091 5.844 1.00 0.00 C

ATOM 232 CG1 VAL A 15 18.514 -0.220 7.087 1.00 0.00 C

ATOM 233 CG2 VAL A 15 19.330 -0.568 4.746 1.00 0.00 C

ATOM 234 H VAL A 15 17.979 -3.035 4.252 1.00 0.00 H

ATOM 235 HA VAL A 15 19.850 -2.609 6.373 1.00 0.00 H

ATOM 236 HB VAL A 15 17.380 -1.052 5.505 1.00 0.00 H

ATOM 237 1HG1 VAL A 15 18.253 0.808 6.833 1.00 0.00 H

ATOM 238 2HG1 VAL A 15 17.825 -0.591 7.846 1.00 0.00 H

ATOM 239 3HG1 VAL A 15 19.532 -0.251 7.474 1.00 0.00 H

ATOM 240 1HG2 VAL A 15 19.067 0.464 4.515 1.00 0.00 H

ATOM 241 2HG2 VAL A 15 20.366 -0.612 5.083 1.00 0.00 H

ATOM 242 3HG2 VAL A 15 19.212 -1.181 3.852 1.00 0.00 H

ATOM 243 N LEU A 16 16.739 -3.263 7.275 1.00 0.00 N

ATOM 244 CA LEU A 16 15.900 -3.658 8.400 1.00 0.00 C

ATOM 245 C LEU A 16 16.315 -5.018 8.945 1.00 0.00 C

ATOM 246 O LEU A 16 16.290 -5.246 10.155 1.00 0.00 O

ATOM 247 CB LEU A 16 14.427 -3.698 7.974 1.00 0.00 C

ATOM 248 CG LEU A 16 13.776 -2.336 7.698 1.00 0.00 C

ATOM 249 CD1 LEU A 16 12.403 -2.546 7.076 1.00 0.00 C

ATOM 250 CD2 LEU A 16 13.674 -1.551 8.997 1.00 0.00 C

ATOM 251 H LEU A 16 16.330 -3.200 6.354 1.00 0.00 H

ATOM 252 HA LEU A 16 16.015 -2.920 9.193 1.00 0.00 H

ATOM 253 1HB LEU A 16 14.344 -4.294 7.066 1.00 0.00 H

ATOM 254 2HB LEU A 16 13.851 -4.188 8.759 1.00 0.00 H

ATOM 255 HG LEU A 16 14.385 -1.780 6.984 1.00 0.00 H

ATOM 256 1HD1 LEU A 16 11.940 -1.578 6.880 1.00 0.00 H

ATOM 257 2HD1 LEU A 16 12.507 -3.093 6.139 1.00 0.00 H

ATOM 258 3HD1 LEU A 16 11.776 -3.115 7.761 1.00 0.00 H

ATOM 259 1HD2 LEU A 16 13.213 -0.582 8.801 1.00 0.00 H

ATOM 260 2HD2 LEU A 16 13.065 -2.105 9.711 1.00 0.00 H

ATOM 261 3HD2 LEU A 16 14.672 -1.400 9.410 1.00 0.00 H

ATOM 262 N ASP A 17 16.695 -5.920 8.047 1.00 0.00 N

ATOM 263 CA ASP A 17 17.183 -7.236 8.441 1.00 0.00 C

ATOM 264 C ASP A 17 18.618 -7.162 8.947 1.00 0.00 C

ATOM 265 O ASP A 17 19.549 -7.598 8.270 1.00 0.00 O

ATOM 266 CB ASP A 17 17.100 -8.213 7.265 1.00 0.00 C

ATOM 267 CG ASP A 17 17.454 -9.641 7.656 1.00 0.00 C

ATOM 268 OD1 ASP A 17 17.619 -9.893 8.827 1.00 0.00 O

ATOM 269 OD2 ASP A 17 17.558 -10.467 6.781 1.00 0.00 O

ATOM 270 H ASP A 17 16.645 -5.687 7.065 1.00 0.00 H

ATOM 271 HA ASP A 17 16.555 -7.610 9.250 1.00 0.00 H

ATOM 272 1HB ASP A 17 16.090 -8.204 6.855 1.00 0.00 H

ATOM 273 2HB ASP A 17 17.777 -7.889 6.474 1.00 0.00 H

ATOM 274 N GLU A 18 18.791 -6.608 10.142 1.00 0.00 N

ATOM 275 CA GLU A 18 20.116 -6.447 10.728 1.00 0.00 C

ATOM 276 C GLU A 18 20.750 -7.798 11.035 1.00 0.00 C

ATOM 277 O GLU A 18 21.968 -7.956 10.950 1.00 0.00 O

ATOM 278 CB GLU A 18 20.037 -5.608 12.005 1.00 0.00 C

ATOM 279 CG GLU A 18 19.690 -4.144 11.775 1.00 0.00 C

ATOM 280 CD GLU A 18 19.579 -3.360 13.053 1.00 0.00 C

ATOM 281 OE1 GLU A 18 19.552 -3.966 14.098 1.00 0.00 O

ATOM 282 OE2 GLU A 18 19.522 -2.155 12.985 1.00 0.00 O

ATOM 283 H GLU A 18 17.983 -6.289 10.658 1.00 0.00 H

ATOM 284 HA GLU A 18 20.752 -5.929 10.009 1.00 0.00 H

ATOM 285 1HB GLU A 18 19.283 -6.029 12.671 1.00 0.00 H

ATOM 286 2HB GLU A 18 20.993 -5.647 12.526 1.00 0.00 H

ATOM 287 1HG GLU A 18 20.461 -3.692 11.151 1.00 0.00 H

ATOM 288 2HG GLU A 18 18.745 -4.085 11.236 1.00 0.00 H

ATOM 289 N ASP A 19 19.917 -8.769 11.392 1.00 0.00 N

ATOM 290 CA ASP A 19 20.396 -10.103 11.732 1.00 0.00 C

ATOM 291 C ASP A 19 20.936 -10.824 10.503 1.00 0.00 C

ATOM 292 O ASP A 19 21.905 -11.579 10.590 1.00 0.00 O

ATOM 293 CB ASP A 19 19.275 -10.931 12.365 1.00 0.00 C

ATOM 294 CG ASP A 19 18.897 -10.450 13.759 1.00 0.00 C

ATOM 295 OD1 ASP A 19 19.638 -9.678 14.321 1.00 0.00 O

ATOM 296 OD2 ASP A 19 17.871 -10.858 14.249 1.00 0.00 O

ATOM 297 H ASP A 19 18.926 -8.578 11.430 1.00 0.00 H

ATOM 298 HA ASP A 19 21.207 -10.006 12.454 1.00 0.00 H

ATOM 299 1HB ASP A 19 18.390 -10.891 11.730 1.00 0.00 H

ATOM 300 2HB ASP A 19 19.585 -11.975 12.428 1.00 0.00 H

ATOM 301 N GLY A 20 20.305 -10.586 9.359 1.00 0.00 N

ATOM 302 CA GLY A 20 20.716 -11.218 8.111 1.00 0.00 C

ATOM 303 C GLY A 20 20.142 -12.623 7.990 1.00 0.00 C

ATOM 304 O GLY A 20 20.814 -13.541 7.518 1.00 0.00 O

ATOM 305 H GLY A 20 19.519 -9.951 9.353 1.00 0.00 H

ATOM 306 1HA GLY A 20 20.383 -10.611 7.269 1.00 0.00 H

ATOM 307 2HA GLY A 20 21.804 -11.260 8.066 1.00 0.00 H

ATOM 308 N LYS A 21 18.895 -12.786 8.418 1.00 0.00 N

ATOM 309 CA LYS A 21 18.260 -14.099 8.448 1.00 0.00 C

ATOM 310 C LYS A 21 17.514 -14.379 7.150 1.00 0.00 C

ATOM 311 O LYS A 21 17.075 -15.503 6.905 1.00 0.00 O

ATOM 312 CB LYS A 21 17.304 -14.205 9.637 1.00 0.00 C

ATOM 313 CG LYS A 21 17.988 -14.186 10.998 1.00 0.00 C

ATOM 314 CD LYS A 21 16.970 -14.216 12.128 1.00 0.00 C

ATOM 315 CE LYS A 21 17.653 -14.248 13.488 1.00 0.00 C

ATOM 316 NZ LYS A 21 16.672 -14.169 14.605 1.00 0.00 N

ATOM 317 H LYS A 21 18.373 -11.980 8.732 1.00 0.00 H

ATOM 318 HA LYS A 21 19.037 -14.856 8.557 1.00 0.00 H

ATOM 319 1HB LYS A 21 16.594 -13.378 9.608 1.00 0.00 H

ATOM 320 2HB LYS A 21 16.732 -15.130 9.563 1.00 0.00 H

ATOM 321 1HG LYS A 21 18.644 -15.053 11.088 1.00 0.00 H

ATOM 322 2HG LYS A 21 18.593 -13.284 11.088 1.00 0.00 H

ATOM 323 1HD LYS A 21 16.336 -13.330 12.070 1.00 0.00 H

ATOM 324 2HD LYS A 21 16.340 -15.099 12.027 1.00 0.00 H

ATOM 325 1HE LYS A 21 18.223 -15.171 13.586 1.00 0.00 H

ATOM 326 2HE LYS A 21 18.344 -13.410 13.568 1.00 0.00 H

ATOM 327 1HZ LYS A 21 17.164 -14.193 15.487 1.00 0.00 H

ATOM 328 2HZ LYS A 21 16.150 -13.307 14.534 1.00 0.00 H

ATOM 329 3HZ LYS A 21 16.037 -14.952 14.552 1.00 0.00 H

ATOM 330 N GLY A 22 17.375 -13.351 6.319 1.00 0.00 N

ATOM 331 CA GLY A 22 16.683 -13.486 5.043 1.00 0.00 C

ATOM 332 C GLY A 22 15.217 -13.090 5.166 1.00 0.00 C

ATOM 333 O GLY A 22 14.428 -13.302 4.245 1.00 0.00 O

ATOM 334 H GLY A 22 17.757 -12.453 6.579 1.00 0.00 H

ATOM 335 1HA GLY A 22 17.172 -12.859 4.297 1.00 0.00 H

ATOM 336 2HA GLY A 22 16.758 -14.516 4.696 1.00 0.00 H

ATOM 337 N TYR A 23 14.859 -12.514 6.308 1.00 0.00 N

ATOM 338 CA TYR A 23 13.497 -12.045 6.536 1.00 0.00 C

ATOM 339 C TYR A 23 13.449 -11.020 7.662 1.00 0.00 C

ATOM 340 O TYR A 23 14.400 -10.883 8.431 1.00 0.00 O

ATOM 341 CB TYR A 23 12.572 -13.223 6.850 1.00 0.00 C

ATOM 342 CG TYR A 23 12.987 -14.018 8.068 1.00 0.00 C

ATOM 343 CD1 TYR A 23 12.585 -13.611 9.332 1.00 0.00 C

ATOM 344 CD2 TYR A 23 13.770 -15.153 7.921 1.00 0.00 C

ATOM 345 CE1 TYR A 23 12.965 -14.338 10.444 1.00 0.00 C

ATOM 346 CE2 TYR A 23 14.149 -15.880 9.033 1.00 0.00 C

ATOM 347 CZ TYR A 23 13.749 -15.475 10.291 1.00 0.00 C

ATOM 348 OH TYR A 23 14.127 -16.199 11.398 1.00 0.00 O

ATOM 349 H TYR A 23 15.548 -12.398 7.038 1.00 0.00 H

ATOM 350 HA TYR A 23 13.144 -11.556 5.628 1.00 0.00 H

ATOM 351 1HB TYR A 23 11.558 -12.855 7.013 1.00 0.00 H

ATOM 352 2HB TYR A 23 12.541 -13.899 5.997 1.00 0.00 H

ATOM 353 HD1 TYR A 23 11.970 -12.719 9.448 1.00 0.00 H

ATOM 354 HD2 TYR A 23 14.086 -15.472 6.928 1.00 0.00 H

ATOM 355 HE1 TYR A 23 12.649 -14.018 11.437 1.00 0.00 H

ATOM 356 HE2 TYR A 23 14.765 -16.772 8.918 1.00 0.00 H

ATOM 357 HH TYR A 23 14.660 -16.949 11.123 1.00 0.00 H

ATOM 358 N ILE A 24 12.335 -10.302 7.753 1.00 0.00 N

ATOM 359 CA ILE A 24 12.178 -9.255 8.756 1.00 0.00 C

ATOM 360 C ILE A 24 11.096 -9.616 9.765 1.00 0.00 C

ATOM 361 O ILE A 24 9.965 -9.930 9.391 1.00 0.00 O

ATOM 362 CB ILE A 24 11.836 -7.908 8.095 1.00 0.00 C

ATOM 363 CG1 ILE A 24 12.956 -7.480 7.143 1.00 0.00 C

ATOM 364 CG2 ILE A 24 11.596 -6.842 9.153 1.00 0.00 C

ATOM 365 CD1 ILE A 24 12.602 -6.292 6.278 1.00 0.00 C

ATOM 366 H ILE A 24 11.577 -10.486 7.112 1.00 0.00 H

ATOM 367 HA ILE A 24 13.120 -9.146 9.292 1.00 0.00 H

ATOM 368 HB ILE A 24 10.935 -8.017 7.492 1.00 0.00 H

ATOM 369 1HG1 ILE A 24 13.847 -7.230 7.718 1.00 0.00 H

ATOM 370 2HG1 ILE A 24 13.215 -8.313 6.488 1.00 0.00 H

ATOM 371 1HG2 ILE A 24 11.356 -5.896 8.668 1.00 0.00 H

ATOM 372 2HG2 ILE A 24 10.767 -7.143 9.791 1.00 0.00 H

ATOM 373 3HG2 ILE A 24 12.495 -6.721 9.758 1.00 0.00 H

ATOM 374 1HD1 ILE A 24 13.445 -6.050 5.630 1.00 0.00 H

ATOM 375 2HD1 ILE A 24 11.732 -6.534 5.666 1.00 0.00 H

ATOM 376 3HD1 ILE A 24 12.374 -5.436 6.911 1.00 0.00 H

ATOM 377 N THR A 25 11.448 -9.570 11.045 1.00 0.00 N

ATOM 378 CA THR A 25 10.488 -9.823 12.113 1.00 0.00 C

ATOM 379 C THR A 25 9.784 -8.540 12.537 1.00 0.00 C

ATOM 380 O THR A 25 10.214 -7.441 12.187 1.00 0.00 O

ATOM 381 CB THR A 25 11.174 -10.464 13.334 1.00 0.00 C

ATOM 382 OG1 THR A 25 12.076 -9.521 13.928 1.00 0.00 O

ATOM 383 CG2 THR A 25 11.946 -11.707 12.921 1.00 0.00 C

ATOM 384 H THR A 25 12.405 -9.354 11.285 1.00 0.00 H

ATOM 385 HA THR A 25 9.732 -10.515 11.742 1.00 0.00 H

ATOM 386 HB THR A 25 10.420 -10.740 14.072 1.00 0.00 H

ATOM 387 HG1 THR A 25 12.851 -9.984 14.256 1.00 0.00 H

ATOM 388 1HG2 THR A 25 12.424 -12.147 13.796 1.00 0.00 H

ATOM 389 2HG2 THR A 25 11.261 -12.431 12.479 1.00 0.00 H

ATOM 390 3HG2 THR A 25 12.708 -11.436 12.191 1.00 0.00 H

ATOM 391 N LYS A 26 8.700 -8.688 13.291 1.00 0.00 N

ATOM 392 CA LYS A 26 7.970 -7.540 13.816 1.00 0.00 C

ATOM 393 C LYS A 26 8.839 -6.721 14.762 1.00 0.00 C

ATOM 394 O LYS A 26 8.718 -5.497 14.829 1.00 0.00 O

ATOM 395 CB LYS A 26 6.698 -7.996 14.531 1.00 0.00 C

ATOM 396 CG LYS A 26 5.619 -8.545 13.607 1.00 0.00 C

ATOM 397 CD LYS A 26 4.377 -8.949 14.387 1.00 0.00 C

ATOM 398 CE LYS A 26 3.272 -9.432 13.459 1.00 0.00 C

ATOM 399 NZ LYS A 26 2.066 -9.872 14.211 1.00 0.00 N

ATOM 400 H LYS A 26 8.374 -9.619 13.507 1.00 0.00 H

ATOM 401 HA LYS A 26 7.690 -6.897 12.981 1.00 0.00 H

ATOM 402 1HB LYS A 26 6.946 -8.772 15.255 1.00 0.00 H

ATOM 403 2HB LYS A 26 6.271 -7.158 15.083 1.00 0.00 H

ATOM 404 1HG LYS A 26 5.347 -7.785 12.874 1.00 0.00 H

ATOM 405 2HG LYS A 26 6.004 -9.416 13.077 1.00 0.00 H

ATOM 406 1HD LYS A 26 4.629 -9.748 15.085 1.00 0.00 H

ATOM 407 2HD LYS A 26 4.011 -8.095 14.957 1.00 0.00 H

ATOM 408 1HE LYS A 26 2.989 -8.628 12.781 1.00 0.00 H

ATOM 409 2HE LYS A 26 3.638 -10.268 12.862 1.00 0.00 H

ATOM 410 1HZ LYS A 26 1.359 -10.185 13.560 1.00 0.00 H

ATOM 411 2HZ LYS A 26 2.313 -10.632 14.830 1.00 0.00 H

ATOM 412 3HZ LYS A 26 1.706 -9.100 14.753 1.00 0.00 H

ATOM 413 N GLU A 27 9.714 -7.402 15.494 1.00 0.00 N

ATOM 414 CA GLU A 27 10.577 -6.743 16.467 1.00 0.00 C

ATOM 415 C GLU A 27 11.656 -5.919 15.777 1.00 0.00 C

ATOM 416 O GLU A 27 12.037 -4.851 16.257 1.00 0.00 O

ATOM 417 CB GLU A 27 11.224 -7.777 17.392 1.00 0.00 C

ATOM 418 CG GLU A 27 10.245 -8.511 18.297 1.00 0.00 C

ATOM 419 CD GLU A 27 9.470 -7.585 19.193 1.00 0.00 C

ATOM 420 OE1 GLU A 27 10.076 -6.742 19.811 1.00 0.00 O

ATOM 421 OE2 GLU A 27 8.271 -7.721 19.260 1.00 0.00 O

ATOM 422 H GLU A 27 9.783 -8.402 15.372 1.00 0.00 H

ATOM 423 HA GLU A 27 9.967 -6.069 17.069 1.00 0.00 H

ATOM 424 1HB GLU A 27 11.750 -8.521 16.794 1.00 0.00 H

ATOM 425 2HB GLU A 27 11.962 -7.286 18.026 1.00 0.00 H

ATOM 426 1HG GLU A 27 9.543 -9.070 17.678 1.00 0.00 H

ATOM 427 2HG GLU A 27 10.796 -9.224 18.908 1.00 0.00 H

ATOM 428 N GLN A 28 12.146 -6.421 14.648 1.00 0.00 N

ATOM 429 CA GLN A 28 13.078 -5.669 13.816 1.00 0.00 C

ATOM 430 C GLN A 28 12.424 -4.413 13.254 1.00 0.00 C

ATOM 431 O GLN A 28 13.048 -3.355 13.183 1.00 0.00 O

ATOM 432 CB GLN A 28 13.601 -6.542 12.673 1.00 0.00 C

ATOM 433 CG GLN A 28 14.589 -7.611 13.109 1.00 0.00 C

ATOM 434 CD GLN A 28 15.044 -8.484 11.955 1.00 0.00 C

ATOM 435 OE1 GLN A 28 14.233 -9.150 11.305 1.00 0.00 O

ATOM 436 NE2 GLN A 28 16.346 -8.486 11.693 1.00 0.00 N

ATOM 437 H GLN A 28 11.865 -7.347 14.359 1.00 0.00 H

ATOM 438 HA GLN A 28 13.921 -5.360 14.434 1.00 0.00 H

ATOM 439 1HB GLN A 28 12.764 -7.038 12.182 1.00 0.00 H

ATOM 440 2HB GLN A 28 14.092 -5.914 11.930 1.00 0.00 H

ATOM 441 1HG GLN A 28 15.466 -7.127 13.538 1.00 0.00 H

ATOM 442 2HG GLN A 28 14.113 -8.249 13.854 1.00 0.00 H

ATOM 443 1HE2 GLN A 28 16.705 -9.043 10.943 1.00 0.00 H

ATOM 444 2HE2 GLN A 28 16.968 -7.932 12.246 1.00 0.00 H

ATOM 445 N LEU A 29 11.163 -4.537 12.854 1.00 0.00 N

ATOM 446 CA LEU A 29 10.401 -3.398 12.357 1.00 0.00 C

ATOM 447 C LEU A 29 10.171 -2.368 13.456 1.00 0.00 C

ATOM 448 O LEU A 29 10.240 -1.163 13.215 1.00 0.00 O

ATOM 449 CB LEU A 29 9.052 -3.866 11.796 1.00 0.00 C

ATOM 450 CG LEU A 29 9.118 -4.673 10.493 1.00 0.00 C

ATOM 451 CD1 LEU A 29 7.753 -5.282 10.203 1.00 0.00 C

ATOM 452 CD2 LEU A 29 9.565 -3.766 9.356 1.00 0.00 C

ATOM 453 H LEU A 29 10.721 -5.444 12.894 1.00 0.00 H

ATOM 454 HA LEU A 29 10.969 -2.924 11.557 1.00 0.00 H

ATOM 455 1HB LEU A 29 8.561 -4.485 12.544 1.00 0.00 H

ATOM 456 2HB LEU A 29 8.430 -2.990 11.612 1.00 0.00 H

ATOM 457 HG LEU A 29 9.831 -5.490 10.607 1.00 0.00 H

ATOM 458 1HD1 LEU A 29 7.800 -5.856 9.277 1.00 0.00 H

ATOM 459 2HD1 LEU A 29 7.467 -5.941 11.023 1.00 0.00 H

ATOM 460 3HD1 LEU A 29 7.015 -4.488 10.100 1.00 0.00 H

ATOM 461 1HD2 LEU A 29 9.613 -4.340 8.430 1.00 0.00 H

ATOM 462 2HD2 LEU A 29 8.852 -2.949 9.240 1.00 0.00 H

ATOM 463 3HD2 LEU A 29 10.551 -3.359 9.581 1.00 0.00 H

ATOM 464 N LYS A 30 9.897 -2.850 14.663 1.00 0.00 N

ATOM 465 CA LYS A 30 9.767 -1.979 15.825 1.00 0.00 C

ATOM 466 C LYS A 30 11.036 -1.166 16.048 1.00 0.00 C

ATOM 467 O LYS A 30 10.982 0.052 16.221 1.00 0.00 O

ATOM 468 CB LYS A 30 9.441 -2.797 17.075 1.00 0.00 C

ATOM 469 CG LYS A 30 8.026 -3.358 17.108 1.00 0.00 C

ATOM 470 CD LYS A 30 7.799 -4.214 18.346 1.00 0.00 C

ATOM 471 CE LYS A 30 6.427 -4.871 18.322 1.00 0.00 C

ATOM 472 NZ LYS A 30 6.218 -5.767 19.491 1.00 0.00 N

ATOM 473 H LYS A 30 9.775 -3.846 14.779 1.00 0.00 H

ATOM 474 HA LYS A 30 8.951 -1.279 15.644 1.00 0.00 H

ATOM 475 1HB LYS A 30 10.135 -3.635 17.153 1.00 0.00 H

ATOM 476 2HB LYS A 30 9.576 -2.177 17.962 1.00 0.00 H

ATOM 477 1HG LYS A 30 7.308 -2.537 17.108 1.00 0.00 H

ATOM 478 2HG LYS A 30 7.855 -3.966 16.220 1.00 0.00 H

ATOM 479 1HD LYS A 30 8.564 -4.990 18.397 1.00 0.00 H

ATOM 480 2HD LYS A 30 7.878 -3.592 19.237 1.00 0.00 H

ATOM 481 1HE LYS A 30 5.656 -4.102 18.326 1.00 0.00 H

ATOM 482 2HE LYS A 30 6.320 -5.456 17.408 1.00 0.00 H

ATOM 483 1HZ LYS A 30 5.299 -6.182 19.438 1.00 0.00 H

ATOM 484 2HZ LYS A 30 6.918 -6.496 19.486 1.00 0.00 H

ATOM 485 3HZ LYS A 30 6.296 -5.232 20.344 1.00 0.00 H

ATOM 486 N LYS A 31 12.177 -1.846 16.041 1.00 0.00 N

ATOM 487 CA LYS A 31 13.465 -1.184 16.216 1.00 0.00 C

ATOM 488 C LYS A 31 13.711 -0.159 15.117 1.00 0.00 C

ATOM 489 O LYS A 31 14.170 0.952 15.382 1.00 0.00 O

ATOM 490 CB LYS A 31 14.597 -2.212 16.240 1.00 0.00 C

ATOM 491 CG LYS A 31 15.978 -1.622 16.492 1.00 0.00 C

ATOM 492 CD LYS A 31 17.046 -2.705 16.513 1.00 0.00 C

ATOM 493 CE LYS A 31 18.421 -2.121 16.804 1.00 0.00 C

ATOM 494 NZ LYS A 31 19.489 -3.156 16.747 1.00 0.00 N

ATOM 495 H LYS A 31 12.153 -2.847 15.911 1.00 0.00 H

ATOM 496 HA LYS A 31 13.455 -0.655 17.170 1.00 0.00 H

ATOM 497 1HB LYS A 31 14.402 -2.950 17.019 1.00 0.00 H

ATOM 498 2HB LYS A 31 14.628 -2.742 15.288 1.00 0.00 H

ATOM 499 1HG LYS A 31 16.215 -0.904 15.706 1.00 0.00 H

ATOM 500 2HG LYS A 31 15.982 -1.100 17.448 1.00 0.00 H

ATOM 501 1HD LYS A 31 16.803 -3.441 17.281 1.00 0.00 H

ATOM 502 2HD LYS A 31 17.073 -3.208 15.547 1.00 0.00 H

ATOM 503 1HE LYS A 31 18.647 -1.343 16.077 1.00 0.00 H

ATOM 504 2HE LYS A 31 18.422 -1.671 17.797 1.00 0.00 H

ATOM 505 1HZ LYS A 31 20.383 -2.730 16.946 1.00 0.00 H

ATOM 506 2HZ LYS A 31 19.299 -3.875 17.432 1.00 0.00 H

ATOM 507 3HZ LYS A 31 19.510 -3.568 15.825 1.00 0.00 H

ATOM 508 N GLY A 32 13.404 -0.539 13.881 1.00 0.00 N

ATOM 509 CA GLY A 32 13.586 0.349 12.739 1.00 0.00 C

ATOM 510 C GLY A 32 12.838 1.661 12.938 1.00 0.00 C

ATOM 511 O GLY A 32 13.380 2.739 12.696 1.00 0.00 O

ATOM 512 H GLY A 32 13.036 -1.467 13.729 1.00 0.00 H

ATOM 513 1HA GLY A 32 14.648 0.549 12.599 1.00 0.00 H

ATOM 514 2HA GLY A 32 13.230 -0.143 11.835 1.00 0.00 H

ATOM 515 N LEU A 33 11.588 1.563 13.380 1.00 0.00 N

ATOM 516 CA LEU A 33 10.776 2.744 13.649 1.00 0.00 C

ATOM 517 C LEU A 33 11.351 3.556 14.802 1.00 0.00 C

ATOM 518 O LEU A 33 11.365 4.787 14.761 1.00 0.00 O

ATOM 519 CB LEU A 33 9.335 2.332 13.974 1.00 0.00 C

ATOM 520 CG LEU A 33 8.519 1.780 12.798 1.00 0.00 C

ATOM 521 CD1 LEU A 33 7.192 1.238 13.310 1.00 0.00 C

ATOM 522 CD2 LEU A 33 8.301 2.880 11.770 1.00 0.00 C

ATOM 523 H LEU A 33 11.191 0.647 13.533 1.00 0.00 H

ATOM 524 HA LEU A 33 10.769 3.370 12.758 1.00 0.00 H

ATOM 525 1HB LEU A 33 9.358 1.568 14.749 1.00 0.00 H

ATOM 526 2HB LEU A 33 8.805 3.201 14.366 1.00 0.00 H

ATOM 527 HG LEU A 33 9.061 0.954 12.336 1.00 0.00 H

ATOM 528 1HD1 LEU A 33 6.612 0.845 12.475 1.00 0.00 H

ATOM 529 2HD1 LEU A 33 7.377 0.440 14.029 1.00 0.00 H

ATOM 530 3HD1 LEU A 33 6.634 2.039 13.793 1.00 0.00 H

ATOM 531 1HD2 LEU A 33 7.722 2.487 10.934 1.00 0.00 H

ATOM 532 2HD2 LEU A 33 7.758 3.706 12.231 1.00 0.00 H

ATOM 533 3HD2 LEU A 33 9.265 3.236 11.408 1.00 0.00 H

ATOM 534 N GLU A 34 11.825 2.862 15.831 1.00 0.00 N

ATOM 535 CA GLU A 34 12.448 3.516 16.975 1.00 0.00 C

ATOM 536 C GLU A 34 13.713 4.258 16.564 1.00 0.00 C

ATOM 537 O GLU A 34 14.006 5.337 17.078 1.00 0.00 O

ATOM 538 CB GLU A 34 12.778 2.490 18.062 1.00 0.00 C

ATOM 539 CG GLU A 34 11.562 1.932 18.789 1.00 0.00 C

ATOM 540 CD GLU A 34 11.912 0.835 19.756 1.00 0.00 C

ATOM 541 OE1 GLU A 34 13.026 0.370 19.721 1.00 0.00 O

ATOM 542 OE2 GLU A 34 11.063 0.461 20.531 1.00 0.00 O

ATOM 543 H GLU A 34 11.751 1.854 15.820 1.00 0.00 H

ATOM 544 HA GLU A 34 11.746 4.243 17.383 1.00 0.00 H

ATOM 545 1HB GLU A 34 13.316 1.651 17.619 1.00 0.00 H

ATOM 546 2HB GLU A 34 13.433 2.944 18.805 1.00 0.00 H

ATOM 547 1HG GLU A 34 11.077 2.741 19.336 1.00 0.00 H

ATOM 548 2HG GLU A 34 10.855 1.551 18.054 1.00 0.00 H

ATOM 549 N LYS A 35 14.460 3.672 15.634 1.00 0.00 N

ATOM 550 CA LYS A 35 15.656 4.311 15.098 1.00 0.00 C

ATOM 551 C LYS A 35 15.309 5.595 14.355 1.00 0.00 C

ATOM 552 O LYS A 35 16.030 6.589 14.444 1.00 0.00 O

ATOM 553 CB LYS A 35 16.406 3.353 14.170 1.00 0.00 C

ATOM 554 CG LYS A 35 17.096 2.198 14.883 1.00 0.00 C

ATOM 555 CD LYS A 35 17.528 1.121 13.900 1.00 0.00 C

ATOM 556 CE LYS A 35 18.745 1.558 13.098 1.00 0.00 C

ATOM 557 NZ LYS A 35 19.174 0.517 12.125 1.00 0.00 N

ATOM 558 H LYS A 35 14.193 2.761 15.290 1.00 0.00 H

ATOM 559 HA LYS A 35 16.311 4.574 15.930 1.00 0.00 H

ATOM 560 1HB LYS A 35 15.710 2.931 13.445 1.00 0.00 H

ATOM 561 2HB LYS A 35 17.164 3.904 13.614 1.00 0.00 H

ATOM 562 1HG LYS A 35 17.974 2.569 15.412 1.00 0.00 H

ATOM 563 2HG LYS A 35 16.413 1.761 15.612 1.00 0.00 H

ATOM 564 1HD LYS A 35 17.770 0.207 14.444 1.00 0.00 H

ATOM 565 2HD LYS A 35 16.710 0.907 13.212 1.00 0.00 H

ATOM 566 1HE LYS A 35 18.513 2.472 12.554 1.00 0.00 H

ATOM 567 2HE LYS A 35 19.572 1.765 13.777 1.00 0.00 H

ATOM 568 1HZ LYS A 35 19.982 0.844 11.615 1.00 0.00 H

ATOM 569 2HZ LYS A 35 19.410 -0.332 12.621 1.00 0.00 H

ATOM 570 3HZ LYS A 35 18.421 0.330 11.478 1.00 0.00 H

ATOM 571 N ASP A 36 14.201 5.568 13.623 1.00 0.00 N

ATOM 572 CA ASP A 36 13.702 6.758 12.944 1.00 0.00 C

ATOM 573 C ASP A 36 13.142 7.765 13.940 1.00 0.00 C

ATOM 574 O ASP A 36 13.230 8.975 13.729 1.00 0.00 O

ATOM 575 CB ASP A 36 12.621 6.382 11.927 1.00 0.00 C

ATOM 576 CG ASP A 36 13.180 5.663 10.707 1.00 0.00 C

ATOM 577 OD1 ASP A 36 14.373 5.702 10.513 1.00 0.00 O

ATOM 578 OD2 ASP A 36 12.410 5.084 9.979 1.00 0.00 O

ATOM 579 H ASP A 36 13.691 4.700 13.534 1.00 0.00 H

ATOM 580 HA ASP A 36 14.531 7.227 12.414 1.00 0.00 H

ATOM 581 1HB ASP A 36 11.882 5.737 12.403 1.00 0.00 H

ATOM 582 2HB ASP A 36 12.104 7.282 11.596 1.00 0.00 H

ATOM 583 N GLY A 37 12.565 7.260 15.025 1.00 0.00 N

ATOM 584 CA GLY A 37 11.966 8.115 16.043 1.00 0.00 C

ATOM 585 C GLY A 37 10.447 8.128 15.927 1.00 0.00 C

ATOM 586 O GLY A 37 9.798 9.117 16.268 1.00 0.00 O

ATOM 587 H GLY A 37 12.540 6.258 15.147 1.00 0.00 H

ATOM 588 1HA GLY A 37 12.256 7.760 17.032 1.00 0.00 H

ATOM 589 2HA GLY A 37 12.351 9.128 15.939 1.00 0.00 H

ATOM 590 N LEU A 38 9.886 7.025 15.445 1.00 0.00 N

ATOM 591 CA LEU A 38 8.447 6.928 15.229 1.00 0.00 C

ATOM 592 C LEU A 38 7.804 5.972 16.225 1.00 0.00 C

ATOM 593 O LEU A 38 8.443 5.034 16.701 1.00 0.00 O

ATOM 594 CB LEU A 38 8.156 6.459 13.798 1.00 0.00 C

ATOM 595 CG LEU A 38 8.754 7.323 12.680 1.00 0.00 C

ATOM 596 CD1 LEU A 38 8.465 6.679 11.331 1.00 0.00 C

ATOM 597 CD2 LEU A 38 8.169 8.725 12.754 1.00 0.00 C

ATOM 598 H LEU A 38 10.471 6.232 15.222 1.00 0.00 H

ATOM 599 HA LEU A 38 8.010 7.916 15.370 1.00 0.00 H

ATOM 600 1HB LEU A 38 8.543 5.448 13.679 1.00 0.00 H

ATOM 601 2HB LEU A 38 7.076 6.431 13.654 1.00 0.00 H

ATOM 602 HG LEU A 38 9.837 7.375 12.800 1.00 0.00 H

ATOM 603 1HD1 LEU A 38 8.890 7.292 10.537 1.00 0.00 H

ATOM 604 2HD1 LEU A 38 8.912 5.685 11.299 1.00 0.00 H

ATOM 605 3HD1 LEU A 38 7.388 6.598 11.190 1.00 0.00 H

ATOM 606 1HD2 LEU A 38 8.594 9.339 11.959 1.00 0.00 H

ATOM 607 2HD2 LEU A 38 7.086 8.674 12.633 1.00 0.00 H

ATOM 608 3HD2 LEU A 38 8.405 9.168 13.721 1.00 0.00 H

ATOM 609 N LYS A 39 6.535 6.215 16.536 1.00 0.00 N

ATOM 610 CA LYS A 39 5.796 5.363 17.460 1.00 0.00 C

ATOM 611 C LYS A 39 5.006 4.295 16.714 1.00 0.00 C

ATOM 612 O LYS A 39 4.709 4.444 15.529 1.00 0.00 O

ATOM 613 CB LYS A 39 4.856 6.203 18.327 1.00 0.00 C

ATOM 614 CG LYS A 39 5.563 7.172 19.265 1.00 0.00 C

ATOM 615 CD LYS A 39 4.566 7.947 20.113 1.00 0.00 C

ATOM 616 CE LYS A 39 5.271 8.915 21.052 1.00 0.00 C

ATOM 617 NZ LYS A 39 4.307 9.691 21.878 1.00 0.00 N

ATOM 618 H LYS A 39 6.070 7.010 16.121 1.00 0.00 H

ATOM 619 HA LYS A 39 6.510 4.857 18.110 1.00 0.00 H

ATOM 620 1HB LYS A 39 4.191 6.783 17.687 1.00 0.00 H

ATOM 621 2HB LYS A 39 4.234 5.544 18.933 1.00 0.00 H

ATOM 622 1HG LYS A 39 6.234 6.618 19.923 1.00 0.00 H

ATOM 623 2HG LYS A 39 6.156 7.876 18.682 1.00 0.00 H

ATOM 624 1HD LYS A 39 3.894 8.509 19.463 1.00 0.00 H

ATOM 625 2HD LYS A 39 3.972 7.251 20.705 1.00 0.00 H

ATOM 626 1HE LYS A 39 5.935 8.362 21.714 1.00 0.00 H

ATOM 627 2HE LYS A 39 5.874 9.613 20.470 1.00 0.00 H

ATOM 628 1HZ LYS A 39 4.813 10.320 22.485 1.00 0.00 H

ATOM 629 2HZ LYS A 39 3.697 10.224 21.274 1.00 0.00 H

ATOM 630 3HZ LYS A 39 3.755 9.056 22.437 1.00 0.00 H

ATOM 631 N LEU A 40 4.670 3.218 17.416 1.00 0.00 N

ATOM 632 CA LEU A 40 3.921 2.118 16.818 1.00 0.00 C

ATOM 633 C LEU A 40 2.445 2.467 16.681 1.00 0.00 C

ATOM 634 O LEU A 40 1.809 2.906 17.640 1.00 0.00 O

ATOM 635 CB LEU A 40 4.077 0.850 17.666 1.00 0.00 C

ATOM 636 CG LEU A 40 5.510 0.323 17.818 1.00 0.00 C

ATOM 637 CD1 LEU A 40 5.512 -0.880 18.752 1.00 0.00 C

ATOM 638 CD2 LEU A 40 6.063 -0.047 16.450 1.00 0.00 C

ATOM 639 H LEU A 40 4.939 3.160 18.387 1.00 0.00 H

ATOM 640 HA LEU A 40 4.323 1.927 15.824 1.00 0.00 H

ATOM 641 1HB LEU A 40 3.690 1.049 18.664 1.00 0.00 H

ATOM 642 2HB LEU A 40 3.478 0.057 17.218 1.00 0.00 H

ATOM 643 HG LEU A 40 6.136 1.094 18.266 1.00 0.00 H

ATOM 644 1HD1 LEU A 40 6.530 -1.254 18.860 1.00 0.00 H

ATOM 645 2HD1 LEU A 40 5.130 -0.582 19.729 1.00 0.00 H

ATOM 646 3HD1 LEU A 40 4.880 -1.664 18.338 1.00 0.00 H

ATOM 647 1HD2 LEU A 40 7.081 -0.421 16.558 1.00 0.00 H

ATOM 648 2HD2 LEU A 40 5.438 -0.820 16.001 1.00 0.00 H

ATOM 649 3HD2 LEU A 40 6.066 0.834 15.808 1.00 0.00 H

ATOM 650 N PRO A 41 1.904 2.269 15.484 1.00 0.00 N

ATOM 651 CA PRO A 41 0.492 2.528 15.229 1.00 0.00 C

ATOM 652 C PRO A 41 -0.387 1.452 15.854 1.00 0.00 C

ATOM 653 O PRO A 41 0.070 0.340 16.119 1.00 0.00 O

ATOM 654 CB PRO A 41 0.409 2.506 13.699 1.00 0.00 C

ATOM 655 CG PRO A 41 1.489 1.565 13.287 1.00 0.00 C

ATOM 656 CD PRO A 41 2.608 1.831 14.258 1.00 0.00 C

ATOM 657 HA PRO A 41 0.229 3.520 15.624 1.00 0.00 H

ATOM 658 1HB PRO A 41 -0.591 2.174 13.381 1.00 0.00 H

ATOM 659 2HB PRO A 41 0.552 3.521 13.300 1.00 0.00 H

ATOM 660 1HG PRO A 41 1.126 0.527 13.334 1.00 0.00 H

ATOM 661 2HG PRO A 41 1.779 1.753 12.243 1.00 0.00 H

ATOM 662 1HD PRO A 41 3.175 0.904 14.429 1.00 0.00 H

ATOM 663 2HD PRO A 41 3.263 2.618 13.856 1.00 0.00 H

ATOM 664 N TYR A 42 -1.650 1.790 16.088 1.00 0.00 N

ATOM 665 CA TYR A 42 -2.586 0.868 16.720 1.00 0.00 C

ATOM 666 C TYR A 42 -2.779 -0.385 15.876 1.00 0.00 C

ATOM 667 O TYR A 42 -3.120 -1.449 16.394 1.00 0.00 O

ATOM 668 CB TYR A 42 -3.931 1.554 16.967 1.00 0.00 C

ATOM 669 CG TYR A 42 -4.759 1.742 15.715 1.00 0.00 C

ATOM 670 CD1 TYR A 42 -5.698 0.786 15.355 1.00 0.00 C

ATOM 671 CD2 TYR A 42 -4.580 2.870 14.928 1.00 0.00 C

ATOM 672 CE1 TYR A 42 -6.454 0.958 14.211 1.00 0.00 C

ATOM 673 CE2 TYR A 42 -5.336 3.041 13.785 1.00 0.00 C

ATOM 674 CZ TYR A 42 -6.270 2.090 13.426 1.00 0.00 C

ATOM 675 OH TYR A 42 -7.023 2.261 12.288 1.00 0.00 O

ATOM 676 H TYR A 42 -1.971 2.710 15.821 1.00 0.00 H

ATOM 677 HA TYR A 42 -2.171 0.558 17.680 1.00 0.00 H

ATOM 678 1HB TYR A 42 -4.515 0.967 17.677 1.00 0.00 H

ATOM 679 2HB TYR A 42 -3.763 2.534 17.414 1.00 0.00 H

ATOM 680 HD1 TYR A 42 -5.838 -0.100 15.974 1.00 0.00 H

ATOM 681 HD2 TYR A 42 -3.842 3.620 15.211 1.00 0.00 H

ATOM 682 HE1 TYR A 42 -7.191 0.207 13.928 1.00 0.00 H

ATOM 683 HE2 TYR A 42 -5.195 3.927 13.166 1.00 0.00 H

ATOM 684 HH TYR A 42 -7.066 3.196 12.070 1.00 0.00 H

ATOM 685 N ASN A 43 -2.560 -0.253 14.572 1.00 0.00 N

ATOM 686 CA ASN A 43 -2.711 -1.375 13.653 1.00 0.00 C

ATOM 687 C ASN A 43 -1.360 -1.845 13.131 1.00 0.00 C

ATOM 688 O ASN A 43 -1.254 -2.341 12.009 1.00 0.00 O

ATOM 689 CB ASN A 43 -3.627 -1.002 12.501 1.00 0.00 C

ATOM 690 CG ASN A 43 -3.091 0.139 11.682 1.00 0.00 C

ATOM 691 OD1 ASN A 43 -2.012 0.670 11.967 1.00 0.00 O

ATOM 692 ND2 ASN A 43 -3.825 0.526 10.670 1.00 0.00 N

ATOM 693 H ASN A 43 -2.282 0.647 14.208 1.00 0.00 H

ATOM 694 HA ASN A 43 -3.157 -2.210 14.195 1.00 0.00 H

ATOM 695 1HB ASN A 43 -3.766 -1.867 11.852 1.00 0.00 H

ATOM 696 2HB ASN A 43 -4.607 -0.725 12.891 1.00 0.00 H

ATOM 697 1HD2 ASN A 43 -3.518 1.281 10.090 1.00 0.00 H

ATOM 698 2HD2 ASN A 43 -4.692 0.068 10.477 1.00 0.00 H

ATOM 699 N PHE A 44 -0.327 -1.687 13.952 1.00 0.00 N

ATOM 700 CA PHE A 44 1.014 -2.132 13.591 1.00 0.00 C

ATOM 701 C PHE A 44 1.027 -3.614 13.242 1.00 0.00 C

ATOM 702 O PHE A 44 1.442 -4.001 12.149 1.00 0.00 O

ATOM 703 CB PHE A 44 1.992 -1.863 14.736 1.00 0.00 C

ATOM 704 CG PHE A 44 3.397 -2.315 14.452 1.00 0.00 C

ATOM 705 CD1 PHE A 44 4.189 -1.632 13.541 1.00 0.00 C

ATOM 706 CD2 PHE A 44 3.928 -3.424 15.093 1.00 0.00 C

ATOM 707 CE1 PHE A 44 5.481 -2.047 13.278 1.00 0.00 C

ATOM 708 CE2 PHE A 44 5.219 -3.840 14.834 1.00 0.00 C

ATOM 709 CZ PHE A 44 5.996 -3.150 13.925 1.00 0.00 C

ATOM 710 H PHE A 44 -0.474 -1.247 14.849 1.00 0.00 H

ATOM 711 HA PHE A 44 1.340 -1.571 12.714 1.00 0.00 H

ATOM 712 1HB PHE A 44 2.016 -0.796 14.952 1.00 0.00 H

ATOM 713 2HB PHE A 44 1.647 -2.371 15.636 1.00 0.00 H

ATOM 714 HD1 PHE A 44 3.782 -0.759 13.030 1.00 0.00 H

ATOM 715 HD2 PHE A 44 3.313 -3.969 15.811 1.00 0.00 H

ATOM 716 HE1 PHE A 44 6.093 -1.501 12.560 1.00 0.00 H

ATOM 717 HE2 PHE A 44 5.625 -4.712 15.345 1.00 0.00 H

ATOM 718 HZ PHE A 44 7.014 -3.479 13.718 1.00 0.00 H

ATOM 719 N ASP A 45 0.571 -4.441 14.177 1.00 0.00 N

ATOM 720 CA ASP A 45 0.534 -5.884 13.972 1.00 0.00 C

ATOM 721 C ASP A 45 -0.483 -6.263 12.903 1.00 0.00 C

ATOM 722 O ASP A 45 -0.256 -7.179 12.112 1.00 0.00 O

ATOM 723 CB ASP A 45 0.200 -6.604 15.281 1.00 0.00 C

ATOM 724 CG ASP A 45 1.350 -6.584 16.278 1.00 0.00 C

ATOM 725 OD1 ASP A 45 2.459 -6.331 15.872 1.00 0.00 O

ATOM 726 OD2 ASP A 45 1.108 -6.821 17.438 1.00 0.00 O

ATOM 727 H ASP A 45 0.242 -4.060 15.052 1.00 0.00 H

ATOM 728 HA ASP A 45 1.519 -6.211 13.636 1.00 0.00 H

ATOM 729 1HB ASP A 45 -0.670 -6.135 15.741 1.00 0.00 H

ATOM 730 2HB ASP A 45 -0.061 -7.641 15.069 1.00 0.00 H

ATOM 731 N LEU A 46 -1.606 -5.552 12.884 1.00 0.00 N

ATOM 732 CA LEU A 46 -2.708 -5.886 11.990 1.00 0.00 C

ATOM 733 C LEU A 46 -2.293 -5.757 10.530 1.00 0.00 C

ATOM 734 O LEU A 46 -2.586 -6.629 9.712 1.00 0.00 O

ATOM 735 CB LEU A 46 -3.910 -4.974 12.268 1.00 0.00 C

ATOM 736 CG LEU A 46 -4.612 -5.190 13.615 1.00 0.00 C

ATOM 737 CD1 LEU A 46 -5.648 -4.096 13.830 1.00 0.00 C

ATOM 738 CD2 LEU A 46 -5.259 -6.567 13.636 1.00 0.00 C

ATOM 739 H LEU A 46 -1.698 -4.761 13.504 1.00 0.00 H

ATOM 740 HA LEU A 46 -3.002 -6.919 12.174 1.00 0.00 H

ATOM 741 1HB LEU A 46 -3.576 -3.939 12.231 1.00 0.00 H

ATOM 742 2HB LEU A 46 -4.650 -5.124 11.482 1.00 0.00 H

ATOM 743 HG LEU A 46 -3.881 -5.121 14.421 1.00 0.00 H

ATOM 744 1HD1 LEU A 46 -6.147 -4.249 14.787 1.00 0.00 H

ATOM 745 2HD1 LEU A 46 -5.155 -3.123 13.830 1.00 0.00 H

ATOM 746 3HD1 LEU A 46 -6.385 -4.130 13.028 1.00 0.00 H

ATOM 747 1HD2 LEU A 46 -5.757 -6.721 14.593 1.00 0.00 H

ATOM 748 2HD2 LEU A 46 -5.991 -6.638 12.831 1.00 0.00 H

ATOM 749 3HD2 LEU A 46 -4.493 -7.331 13.498 1.00 0.00 H

ATOM 750 N LEU A 47 -1.608 -4.665 10.209 1.00 0.00 N

ATOM 751 CA LEU A 47 -1.135 -4.429 8.851 1.00 0.00 C

ATOM 752 C LEU A 47 -0.089 -5.459 8.444 1.00 0.00 C

ATOM 753 O LEU A 47 -0.077 -5.931 7.308 1.00 0.00 O

ATOM 754 CB LEU A 47 -0.545 -3.018 8.732 1.00 0.00 C

ATOM 755 CG LEU A 47 -1.554 -1.865 8.825 1.00 0.00 C

ATOM 756 CD1 LEU A 47 -0.807 -0.538 8.859 1.00 0.00 C

ATOM 757 CD2 LEU A 47 -2.505 -1.927 7.639 1.00 0.00 C

ATOM 758 H LEU A 47 -1.410 -3.980 10.925 1.00 0.00 H

ATOM 759 HA LEU A 47 -1.982 -4.513 8.171 1.00 0.00 H

ATOM 760 1HB LEU A 47 0.188 -2.880 9.525 1.00 0.00 H

ATOM 761 2HB LEU A 47 -0.033 -2.934 7.774 1.00 0.00 H

ATOM 762 HG LEU A 47 -2.122 -1.952 9.751 1.00 0.00 H

ATOM 763 1HD1 LEU A 47 -1.524 0.280 8.925 1.00 0.00 H

ATOM 764 2HD1 LEU A 47 -0.149 -0.514 9.727 1.00 0.00 H

ATOM 765 3HD1 LEU A 47 -0.216 -0.430 7.951 1.00 0.00 H

ATOM 766 1HD2 LEU A 47 -3.222 -1.108 7.705 1.00 0.00 H

ATOM 767 2HD2 LEU A 47 -1.938 -1.839 6.712 1.00 0.00 H

ATOM 768 3HD2 LEU A 47 -3.038 -2.878 7.649 1.00 0.00 H

ATOM 769 N LEU A 48 0.788 -5.804 9.380 1.00 0.00 N

ATOM 770 CA LEU A 48 1.865 -6.749 9.110 1.00 0.00 C

ATOM 771 C LEU A 48 1.327 -8.166 8.952 1.00 0.00 C

ATOM 772 O LEU A 48 1.881 -8.971 8.203 1.00 0.00 O

ATOM 773 CB LEU A 48 2.900 -6.712 10.241 1.00 0.00 C

ATOM 774 CG LEU A 48 3.694 -5.406 10.373 1.00 0.00 C

ATOM 775 CD1 LEU A 48 4.590 -5.478 11.602 1.00 0.00 C

ATOM 776 CD2 LEU A 48 4.513 -5.180 9.111 1.00 0.00 C

ATOM 777 H LEU A 48 0.708 -5.401 10.303 1.00 0.00 H

ATOM 778 HA LEU A 48 2.352 -6.461 8.179 1.00 0.00 H

ATOM 779 1HB LEU A 48 2.388 -6.885 11.186 1.00 0.00 H

ATOM 780 2HB LEU A 48 3.615 -7.520 10.085 1.00 0.00 H

ATOM 781 HG LEU A 48 3.004 -4.573 10.512 1.00 0.00 H

ATOM 782 1HD1 LEU A 48 5.154 -4.550 11.696 1.00 0.00 H

ATOM 783 2HD1 LEU A 48 3.977 -5.620 12.492 1.00 0.00 H

ATOM 784 3HD1 LEU A 48 5.282 -6.313 11.499 1.00 0.00 H

ATOM 785 1HD2 LEU A 48 5.077 -4.251 9.205 1.00 0.00 H

ATOM 786 2HD2 LEU A 48 5.205 -6.012 8.972 1.00 0.00 H

ATOM 787 3HD2 LEU A 48 3.847 -5.115 8.251 1.00 0.00 H

ATOM 788 N ASP A 49 0.245 -8.465 9.662 1.00 0.00 N

ATOM 789 CA ASP A 49 -0.433 -9.748 9.522 1.00 0.00 C

ATOM 790 C ASP A 49 -1.080 -9.882 8.149 1.00 0.00 C

ATOM 791 O ASP A 49 -1.116 -10.969 7.572 1.00 0.00 O

ATOM 792 CB ASP A 49 -1.495 -9.916 10.611 1.00 0.00 C

ATOM 793 CG ASP A 49 -0.896 -10.162 11.990 1.00 0.00 C

ATOM 794 OD1 ASP A 49 0.269 -10.472 12.063 1.00 0.00 O

ATOM 795 OD2 ASP A 49 -1.610 -10.038 12.956 1.00 0.00 O

ATOM 796 H ASP A 49 -0.118 -7.786 10.316 1.00 0.00 H

ATOM 797 HA ASP A 49 0.305 -10.543 9.630 1.00 0.00 H

ATOM 798 1HB ASP A 49 -2.116 -9.021 10.656 1.00 0.00 H

ATOM 799 2HB ASP A 49 -2.146 -10.753 10.359 1.00 0.00 H

ATOM 800 N GLN A 50 -1.591 -8.770 7.630 1.00 0.00 N

ATOM 801 CA GLN A 50 -2.112 -8.729 6.269 1.00 0.00 C

ATOM 802 C GLN A 50 -0.998 -8.914 5.247 1.00 0.00 C

ATOM 803 O GLN A 50 -1.166 -9.618 4.251 1.00 0.00 O

ATOM 804 CB GLN A 50 -2.841 -7.407 6.014 1.00 0.00 C

ATOM 805 CG GLN A 50 -4.143 -7.256 6.783 1.00 0.00 C

ATOM 806 CD GLN A 50 -4.764 -5.884 6.604 1.00 0.00 C

ATOM 807 OE1 GLN A 50 -4.099 -4.936 6.178 1.00 0.00 O

ATOM 808 NE2 GLN A 50 -6.047 -5.770 6.929 1.00 0.00 N

ATOM 809 H GLN A 50 -1.619 -7.932 8.193 1.00 0.00 H

ATOM 810 HA GLN A 50 -2.821 -9.547 6.145 1.00 0.00 H

ATOM 811 1HB GLN A 50 -2.191 -6.575 6.286 1.00 0.00 H

ATOM 812 2HB GLN A 50 -3.065 -7.314 4.952 1.00 0.00 H

ATOM 813 1HG GLN A 50 -4.852 -8.002 6.426 1.00 0.00 H

ATOM 814 2HG GLN A 50 -3.945 -7.406 7.844 1.00 0.00 H

ATOM 815 1HE2 GLN A 50 -6.512 -4.888 6.832 1.00 0.00 H

ATOM 816 2HE2 GLN A 50 -6.549 -6.564 7.271 1.00 0.00 H

ATOM 817 N ILE A 51 0.141 -8.277 5.499 1.00 0.00 N

ATOM 818 CA ILE A 51 1.313 -8.436 4.646 1.00 0.00 C

ATOM 819 C ILE A 51 1.831 -9.868 4.681 1.00 0.00 C

ATOM 820 O ILE A 51 2.259 -10.409 3.662 1.00 0.00 O

ATOM 821 CB ILE A 51 2.436 -7.472 5.070 1.00 0.00 C

ATOM 822 CG1 ILE A 51 2.032 -6.023 4.787 1.00 0.00 C

ATOM 823 CG2 ILE A 51 3.732 -7.817 4.352 1.00 0.00 C

ATOM 824 CD1 ILE A 51 2.953 -4.999 5.410 1.00 0.00 C

ATOM 825 H ILE A 51 0.197 -7.668 6.303 1.00 0.00 H

ATOM 826 HA ILE A 51 1.027 -8.203 3.621 1.00 0.00 H

ATOM 827 HB ILE A 51 2.595 -7.551 6.145 1.00 0.00 H

ATOM 828 1HG1 ILE A 51 2.012 -5.855 3.711 1.00 0.00 H

ATOM 829 2HG1 ILE A 51 1.024 -5.846 5.163 1.00 0.00 H

ATOM 830 1HG2 ILE A 51 4.515 -7.126 4.663 1.00 0.00 H

ATOM 831 2HG2 ILE A 51 4.026 -8.835 4.602 1.00 0.00 H

ATOM 832 3HG2 ILE A 51 3.583 -7.736 3.275 1.00 0.00 H

ATOM 833 1HD1 ILE A 51 2.602 -3.997 5.165 1.00 0.00 H

ATOM 834 2HD1 ILE A 51 2.961 -5.127 6.493 1.00 0.00 H

ATOM 835 3HD1 ILE A 51 3.962 -5.134 5.021 1.00 0.00 H

ATOM 836 N ASP A 52 1.788 -10.478 5.861 1.00 0.00 N

ATOM 837 CA ASP A 52 2.230 -11.858 6.027 1.00 0.00 C

ATOM 838 C ASP A 52 1.292 -12.825 5.317 1.00 0.00 C

ATOM 839 O ASP A 52 0.484 -13.502 5.954 1.00 0.00 O

ATOM 840 CB ASP A 52 2.317 -12.218 7.512 1.00 0.00 C

ATOM 841 CG ASP A 52 2.893 -13.608 7.751 1.00 0.00 C

ATOM 842 OD1 ASP A 52 3.212 -14.270 6.792 1.00 0.00 O

ATOM 843 OD2 ASP A 52 3.009 -13.992 8.890 1.00 0.00 O

ATOM 844 H ASP A 52 1.441 -9.973 6.664 1.00 0.00 H

ATOM 845 HA ASP A 52 3.221 -11.959 5.585 1.00 0.00 H

ATOM 846 1HB ASP A 52 2.941 -11.488 8.028 1.00 0.00 H

ATOM 847 2HB ASP A 52 1.322 -12.172 7.956 1.00 0.00 H

ATOM 848 N SER A 53 1.403 -12.887 3.994 1.00 0.00 N

ATOM 849 CA SER A 53 0.390 -13.534 3.169 1.00 0.00 C

ATOM 850 C SER A 53 0.405 -15.045 3.363 1.00 0.00 C

ATOM 851 O SER A 53 -0.635 -15.700 3.291 1.00 0.00 O

ATOM 852 CB SER A 53 0.616 -13.201 1.708 1.00 0.00 C

ATOM 853 OG SER A 53 1.835 -13.723 1.256 1.00 0.00 O

ATOM 854 H SER A 53 2.210 -12.474 3.549 1.00 0.00 H

ATOM 855 HA SER A 53 -0.591 -13.160 3.468 1.00 0.00 H

ATOM 856 1HB SER A 53 -0.200 -13.608 1.111 1.00 0.00 H

ATOM 857 2HB SER A 53 0.609 -12.120 1.576 1.00 0.00 H

ATOM 858 HG SER A 53 2.449 -13.632 1.989 1.00 0.00 H

ATOM 859 N ASP A 54 1.590 -15.593 3.610 1.00 0.00 N

ATOM 860 CA ASP A 54 1.760 -17.037 3.711 1.00 0.00 C

ATOM 861 C ASP A 54 1.704 -17.499 5.162 1.00 0.00 C

ATOM 862 O ASP A 54 1.953 -18.666 5.462 1.00 0.00 O

ATOM 863 CB ASP A 54 3.089 -17.466 3.084 1.00 0.00 C

ATOM 864 CG ASP A 54 4.299 -16.955 3.853 1.00 0.00 C

ATOM 865 OD1 ASP A 54 4.113 -16.353 4.884 1.00 0.00 O

ATOM 866 OD2 ASP A 54 5.399 -17.170 3.402 1.00 0.00 O

ATOM 867 H ASP A 54 2.393 -14.993 3.731 1.00 0.00 H

ATOM 868 HA ASP A 54 0.947 -17.520 3.168 1.00 0.00 H

ATOM 869 1HB ASP A 54 3.137 -18.555 3.042 1.00 0.00 H

ATOM 870 2HB ASP A 54 3.144 -17.096 2.060 1.00 0.00 H

ATOM 871 N GLY A 55 1.374 -16.576 6.059 1.00 0.00 N

ATOM 872 CA GLY A 55 1.070 -16.925 7.441 1.00 0.00 C

ATOM 873 C GLY A 55 2.260 -17.597 8.114 1.00 0.00 C

ATOM 874 O GLY A 55 2.095 -18.530 8.900 1.00 0.00 O

ATOM 875 H GLY A 55 1.333 -15.607 5.775 1.00 0.00 H

ATOM 876 1HA GLY A 55 0.796 -16.024 7.992 1.00 0.00 H

ATOM 877 2HA GLY A 55 0.209 -17.591 7.467 1.00 0.00 H

ATOM 878 N SER A 56 3.459 -17.117 7.801 1.00 0.00 N

ATOM 879 CA SER A 56 4.684 -17.732 8.299 1.00 0.00 C

ATOM 880 C SER A 56 5.169 -17.044 9.568 1.00 0.00 C

ATOM 881 O SER A 56 6.135 -17.482 10.193 1.00 0.00 O

ATOM 882 CB SER A 56 5.765 -17.675 7.238 1.00 0.00 C

ATOM 883 OG SER A 56 6.086 -16.349 6.918 1.00 0.00 O

ATOM 884 H SER A 56 3.523 -16.306 7.202 1.00 0.00 H

ATOM 885 HA SER A 56 4.476 -18.777 8.534 1.00 0.00 H

ATOM 886 1HB SER A 56 6.655 -18.191 7.597 1.00 0.00 H

ATOM 887 2HB SER A 56 5.425 -18.195 6.343 1.00 0.00 H

ATOM 888 HG SER A 56 5.472 -16.086 6.229 1.00 0.00 H

ATOM 889 N GLY A 57 4.492 -15.964 9.945 1.00 0.00 N

ATOM 890 CA GLY A 57 4.816 -15.247 11.173 1.00 0.00 C

ATOM 891 C GLY A 57 6.007 -14.320 10.971 1.00 0.00 C

ATOM 892 O GLY A 57 6.721 -13.995 11.920 1.00 0.00 O

ATOM 893 H GLY A 57 3.734 -15.631 9.367 1.00 0.00 H

ATOM 894 1HA GLY A 57 3.950 -14.669 11.496 1.00 0.00 H

ATOM 895 2HA GLY A 57 5.037 -15.962 11.964 1.00 0.00 H

ATOM 896 N LYS A 58 6.217 -13.895 9.730 1.00 0.00 N

ATOM 897 CA LYS A 58 7.373 -13.076 9.385 1.00 0.00 C

ATOM 898 C LYS A 58 7.182 -12.394 8.036 1.00 0.00 C

ATOM 899 O LYS A 58 6.362 -12.822 7.224 1.00 0.00 O

ATOM 900 CB LYS A 58 8.645 -13.925 9.369 1.00 0.00 C

ATOM 901 CG LYS A 58 8.619 -15.079 8.375 1.00 0.00 C

ATOM 902 CD LYS A 58 9.848 -15.964 8.524 1.00 0.00 C

ATOM 903 CE LYS A 58 9.808 -17.135 7.553 1.00 0.00 C

ATOM 904 NZ LYS A 58 10.995 -18.020 7.701 1.00 0.00 N

ATOM 905 H LYS A 58 5.558 -14.146 9.006 1.00 0.00 H

ATOM 906 HA LYS A 58 7.483 -12.297 10.140 1.00 0.00 H

ATOM 907 1HB LYS A 58 9.501 -13.294 9.128 1.00 0.00 H

ATOM 908 2HB LYS A 58 8.816 -14.343 10.362 1.00 0.00 H

ATOM 909 1HG LYS A 58 7.726 -15.681 8.540 1.00 0.00 H

ATOM 910 2HG LYS A 58 8.588 -14.683 7.360 1.00 0.00 H

ATOM 911 1HD LYS A 58 10.746 -15.375 8.334 1.00 0.00 H

ATOM 912 2HD LYS A 58 9.897 -16.350 9.542 1.00 0.00 H

ATOM 913 1HE LYS A 58 8.908 -17.722 7.728 1.00 0.00 H

ATOM 914 2HE LYS A 58 9.777 -16.759 6.531 1.00 0.00 H

ATOM 915 1HZ LYS A 58 10.931 -18.782 7.041 1.00 0.00 H

ATOM 916 2HZ LYS A 58 11.836 -17.490 7.522 1.00 0.00 H

ATOM 917 3HZ LYS A 58 11.025 -18.391 8.640 1.00 0.00 H

ATOM 918 N ILE A 59 7.943 -11.331 7.804 1.00 0.00 N

ATOM 919 CA ILE A 59 7.876 -10.601 6.543 1.00 0.00 C

ATOM 920 C ILE A 59 8.872 -11.155 5.532 1.00 0.00 C

ATOM 921 O ILE A 59 10.081 -10.968 5.671 1.00 0.00 O

ATOM 922 CB ILE A 59 8.146 -9.101 6.762 1.00 0.00 C

ATOM 923 CG1 ILE A 59 7.194 -8.535 7.818 1.00 0.00 C

ATOM 924 CG2 ILE A 59 8.008 -8.339 5.453 1.00 0.00 C

ATOM 925 CD1 ILE A 59 5.731 -8.691 7.467 1.00 0.00 C

ATOM 926 H ILE A 59 8.586 -11.020 8.518 1.00 0.00 H

ATOM 927 HA ILE A 59 6.874 -10.714 6.131 1.00 0.00 H

ATOM 928 HB ILE A 59 9.157 -8.966 7.145 1.00 0.00 H

ATOM 929 1HG1 ILE A 59 7.371 -9.032 8.771 1.00 0.00 H

ATOM 930 2HG1 ILE A 59 7.398 -7.474 7.961 1.00 0.00 H

ATOM 931 1HG2 ILE A 59 8.201 -7.281 5.626 1.00 0.00 H

ATOM 932 2HG2 ILE A 59 8.725 -8.726 4.730 1.00 0.00 H

ATOM 933 3HG2 ILE A 59 6.997 -8.464 5.064 1.00 0.00 H

ATOM 934 1HD1 ILE A 59 5.119 -8.266 8.263 1.00 0.00 H

ATOM 935 2HD1 ILE A 59 5.524 -8.169 6.532 1.00 0.00 H

ATOM 936 3HD1 ILE A 59 5.495 -9.748 7.354 1.00 0.00 H

ATOM 937 N ASP A 60 8.356 -11.837 4.515 1.00 0.00 N

ATOM 938 CA ASP A 60 9.201 -12.445 3.494 1.00 0.00 C

ATOM 939 C ASP A 60 9.827 -11.386 2.594 1.00 0.00 C

ATOM 940 O ASP A 60 9.318 -10.271 2.485 1.00 0.00 O

ATOM 941 CB ASP A 60 8.394 -13.430 2.646 1.00 0.00 C

ATOM 942 CG ASP A 60 9.270 -14.314 1.768 1.00 0.00 C

ATOM 943 OD1 ASP A 60 9.807 -15.272 2.272 1.00 0.00 O

ATOM 944 OD2 ASP A 60 9.394 -14.022 0.603 1.00 0.00 O

ATOM 945 H ASP A 60 7.354 -11.936 4.447 1.00 0.00 H

ATOM 946 HA ASP A 60 10.006 -12.989 3.989 1.00 0.00 H

ATOM 947 1HB ASP A 60 7.798 -14.069 3.299 1.00 0.00 H

ATOM 948 2HB ASP A 60 7.702 -12.880 2.007 1.00 0.00 H

ATOM 949 N TYR A 61 10.933 -11.742 1.951 1.00 0.00 N

ATOM 950 CA TYR A 61 11.552 -10.878 0.953 1.00 0.00 C

ATOM 951 C TYR A 61 10.532 -10.409 -0.077 1.00 0.00 C

ATOM 952 O TYR A 61 10.503 -9.235 -0.446 1.00 0.00 O

ATOM 953 CB TYR A 61 12.710 -11.601 0.263 1.00 0.00 C

ATOM 954 CG TYR A 61 13.365 -10.793 -0.836 1.00 0.00 C

ATOM 955 CD1 TYR A 61 14.256 -9.780 -0.516 1.00 0.00 C

ATOM 956 CD2 TYR A 61 13.075 -11.067 -2.165 1.00 0.00 C

ATOM 957 CE1 TYR A 61 14.854 -9.042 -1.519 1.00 0.00 C

ATOM 958 CE2 TYR A 61 13.672 -10.330 -3.168 1.00 0.00 C

ATOM 959 CZ TYR A 61 14.559 -9.321 -2.849 1.00 0.00 C

ATOM 960 OH TYR A 61 15.154 -8.587 -3.849 1.00 0.00 O

ATOM 961 H TYR A 61 11.357 -12.635 2.159 1.00 0.00 H

ATOM 962 HA TYR A 61 11.943 -9.993 1.457 1.00 0.00 H

ATOM 963 1HB TYR A 61 13.473 -11.853 1.000 1.00 0.00 H

ATOM 964 2HB TYR A 61 12.351 -12.534 -0.169 1.00 0.00 H

ATOM 965 HD1 TYR A 61 14.484 -9.565 0.529 1.00 0.00 H

ATOM 966 HD2 TYR A 61 12.374 -11.863 -2.417 1.00 0.00 H

ATOM 967 HE1 TYR A 61 15.554 -8.246 -1.267 1.00 0.00 H

ATOM 968 HE2 TYR A 61 13.444 -10.544 -4.213 1.00 0.00 H

ATOM 969 HH TYR A 61 15.812 -8.001 -3.466 1.00 0.00 H

ATOM 970 N THR A 62 9.697 -11.334 -0.538 1.00 0.00 N

ATOM 971 CA THR A 62 8.735 -11.039 -1.594 1.00 0.00 C

ATOM 972 C THR A 62 7.511 -10.321 -1.040 1.00 0.00 C

ATOM 973 O THR A 62 6.901 -9.498 -1.723 1.00 0.00 O

ATOM 974 CB THR A 62 8.296 -12.325 -2.318 1.00 0.00 C

ATOM 975 OG1 THR A 62 7.675 -13.216 -1.382 1.00 0.00 O

ATOM 976 CG2 THR A 62 9.495 -13.017 -2.951 1.00 0.00 C

ATOM 977 H THR A 62 9.728 -12.264 -0.146 1.00 0.00 H

ATOM 978 HA THR A 62 9.211 -10.381 -2.321 1.00 0.00 H

ATOM 979 HB THR A 62 7.575 -12.077 -3.096 1.00 0.00 H

ATOM 980 HG1 THR A 62 8.305 -13.447 -0.695 1.00 0.00 H

ATOM 981 1HG2 THR A 62 9.165 -13.923 -3.459 1.00 0.00 H

ATOM 982 2HG2 THR A 62 9.962 -12.346 -3.672 1.00 0.00 H

ATOM 983 3HG2 THR A 62 10.215 -13.276 -2.177 1.00 0.00 H

ATOM 984 N GLU A 63 7.157 -10.637 0.201 1.00 0.00 N

ATOM 985 CA GLU A 63 6.054 -9.965 0.877 1.00 0.00 C

ATOM 986 C GLU A 63 6.384 -8.504 1.152 1.00 0.00 C

ATOM 987 O GLU A 63 5.538 -7.625 0.990 1.00 0.00 O

ATOM 988 CB GLU A 63 5.720 -10.678 2.190 1.00 0.00 C

ATOM 989 CG GLU A 63 5.030 -12.024 2.016 1.00 0.00 C

ATOM 990 CD GLU A 63 4.754 -12.712 3.324 1.00 0.00 C

ATOM 991 OE1 GLU A 63 5.472 -12.472 4.264 1.00 0.00 O

ATOM 992 OE2 GLU A 63 3.822 -13.480 3.383 1.00 0.00 O

ATOM 993 H GLU A 63 7.665 -11.362 0.688 1.00 0.00 H

ATOM 994 HA GLU A 63 5.178 -9.999 0.229 1.00 0.00 H

ATOM 995 1HB GLU A 63 6.635 -10.842 2.758 1.00 0.00 H

ATOM 996 2HB GLU A 63 5.069 -10.044 2.792 1.00 0.00 H

ATOM 997 1HG GLU A 63 4.087 -11.873 1.492 1.00 0.00 H

ATOM 998 2HG GLU A 63 5.658 -12.665 1.399 1.00 0.00 H

ATOM 999 N PHE A 64 7.620 -8.250 1.569 1.00 0.00 N

ATOM 1000 CA PHE A 64 8.071 -6.892 1.847 1.00 0.00 C

ATOM 1001 C PHE A 64 7.938 -6.004 0.617 1.00 0.00 C

ATOM 1002 O PHE A 64 7.360 -4.919 0.682 1.00 0.00 O

ATOM 1003 CB PHE A 64 9.525 -6.899 2.323 1.00 0.00 C

ATOM 1004 CG PHE A 64 10.037 -5.545 2.725 1.00 0.00 C

ATOM 1005 CD1 PHE A 64 9.960 -5.122 4.043 1.00 0.00 C

ATOM 1006 CD2 PHE A 64 10.597 -4.691 1.786 1.00 0.00 C

ATOM 1007 CE1 PHE A 64 10.431 -3.877 4.415 1.00 0.00 C

ATOM 1008 CE2 PHE A 64 11.069 -3.447 2.154 1.00 0.00 C

ATOM 1009 CZ PHE A 64 10.986 -3.039 3.471 1.00 0.00 C

ATOM 1010 H PHE A 64 8.264 -9.018 1.696 1.00 0.00 H

ATOM 1011 HA PHE A 64 7.447 -6.476 2.639 1.00 0.00 H

ATOM 1012 1HB PHE A 64 9.626 -7.568 3.176 1.00 0.00 H

ATOM 1013 2HB PHE A 64 10.165 -7.282 1.529 1.00 0.00 H

ATOM 1014 HD1 PHE A 64 9.522 -5.785 4.790 1.00 0.00 H

ATOM 1015 HD2 PHE A 64 10.663 -5.013 0.746 1.00 0.00 H

ATOM 1016 HE1 PHE A 64 10.363 -3.557 5.454 1.00 0.00 H

ATOM 1017 HE2 PHE A 64 11.507 -2.786 1.407 1.00 0.00 H

ATOM 1018 HZ PHE A 64 11.356 -2.057 3.762 1.00 0.00 H

ATOM 1019 N ILE A 65 8.476 -6.471 -0.505 1.00 0.00 N

ATOM 1020 CA ILE A 65 8.484 -5.688 -1.735 1.00 0.00 C

ATOM 1021 C ILE A 65 7.083 -5.567 -2.320 1.00 0.00 C

ATOM 1022 O ILE A 65 6.651 -4.479 -2.700 1.00 0.00 O

ATOM 1023 CB ILE A 65 9.425 -6.317 -2.779 1.00 0.00 C

ATOM 1024 CG1 ILE A 65 10.879 -6.230 -2.309 1.00 0.00 C

ATOM 1025 CG2 ILE A 65 9.256 -5.631 -4.127 1.00 0.00 C

ATOM 1026 CD1 ILE A 65 11.838 -7.059 -3.133 1.00 0.00 C

ATOM 1027 H ILE A 65 8.891 -7.392 -0.505 1.00 0.00 H

ATOM 1028 HA ILE A 65 8.845 -4.687 -1.505 1.00 0.00 H

ATOM 1029 HB ILE A 65 9.192 -7.375 -2.889 1.00 0.00 H

ATOM 1030 1HG1 ILE A 65 11.211 -5.193 -2.340 1.00 0.00 H

ATOM 1031 2HG1 ILE A 65 10.947 -6.563 -1.273 1.00 0.00 H

ATOM 1032 1HG2 ILE A 65 9.929 -6.087 -4.853 1.00 0.00 H

ATOM 1033 2HG2 ILE A 65 8.227 -5.743 -4.465 1.00 0.00 H

ATOM 1034 3HG2 ILE A 65 9.493 -4.572 -4.028 1.00 0.00 H

ATOM 1035 1HD1 ILE A 65 12.848 -6.947 -2.739 1.00 0.00 H

ATOM 1036 2HD1 ILE A 65 11.545 -8.108 -3.087 1.00 0.00 H

ATOM 1037 3HD1 ILE A 65 11.814 -6.720 -4.168 1.00 0.00 H

ATOM 1038 N ALA A 66 6.377 -6.691 -2.389 1.00 0.00 N

ATOM 1039 CA ALA A 66 5.055 -6.728 -3.003 1.00 0.00 C

ATOM 1040 C ALA A 66 4.080 -5.818 -2.269 1.00 0.00 C

ATOM 1041 O ALA A 66 3.279 -5.120 -2.890 1.00 0.00 O

ATOM 1042 CB ALA A 66 4.526 -8.155 -3.034 1.00 0.00 C

ATOM 1043 H ALA A 66 6.766 -7.540 -2.005 1.00 0.00 H

ATOM 1044 HA ALA A 66 5.147 -6.363 -4.026 1.00 0.00 H

ATOM 1045 1HB ALA A 66 3.539 -8.166 -3.496 1.00 0.00 H

ATOM 1046 2HB ALA A 66 5.205 -8.782 -3.613 1.00 0.00 H

ATOM 1047 3HB ALA A 66 4.456 -8.538 -2.018 1.00 0.00 H

ATOM 1048 N ALA A 67 4.153 -5.828 -0.942 1.00 0.00 N

ATOM 1049 CA ALA A 67 3.278 -5.002 -0.120 1.00 0.00 C

ATOM 1050 C ALA A 67 3.813 -3.581 -0.002 1.00 0.00 C

ATOM 1051 O ALA A 67 3.112 -2.679 0.458 1.00 0.00 O

ATOM 1052 CB ALA A 67 3.108 -5.620 1.261 1.00 0.00 C

ATOM 1053 H ALA A 67 4.833 -6.425 -0.491 1.00 0.00 H

ATOM 1054 HA ALA A 67 2.303 -4.952 -0.606 1.00 0.00 H

ATOM 1055 1HB ALA A 67 2.452 -4.991 1.863 1.00 0.00 H

ATOM 1056 2HB ALA A 67 2.670 -6.613 1.163 1.00 0.00 H

ATOM 1057 3HB ALA A 67 4.080 -5.698 1.745 1.00 0.00 H

ATOM 1058 N ALA A 68 5.059 -3.387 -0.420 1.00 0.00 N

ATOM 1059 CA ALA A 68 5.709 -2.086 -0.314 1.00 0.00 C

ATOM 1060 C ALA A 68 5.739 -1.601 1.130 1.00 0.00 C

ATOM 1061 O ALA A 68 5.286 -0.498 1.435 1.00 0.00 O

ATOM 1062 CB ALA A 68 5.005 -1.068 -1.199 1.00 0.00 C

ATOM 1063 H ALA A 68 5.569 -4.160 -0.822 1.00 0.00 H

ATOM 1064 HA ALA A 68 6.740 -2.194 -0.651 1.00 0.00 H

ATOM 1065 1HB ALA A 68 5.502 -0.102 -1.108 1.00 0.00 H

ATOM 1066 2HB ALA A 68 5.043 -1.400 -2.236 1.00 0.00 H

ATOM 1067 3HB ALA A 68 3.966 -0.972 -0.886 1.00 0.00 H

ATOM 1068 N LEU A 69 6.276 -2.433 2.017 1.00 0.00 N

ATOM 1069 CA LEU A 69 6.393 -2.078 3.426 1.00 0.00 C

ATOM 1070 C LEU A 69 7.557 -1.123 3.659 1.00 0.00 C

ATOM 1071 O LEU A 69 8.659 -1.338 3.155 1.00 0.00 O

ATOM 1072 CB LEU A 69 6.582 -3.341 4.277 1.00 0.00 C

ATOM 1073 CG LEU A 69 6.797 -3.106 5.777 1.00 0.00 C

ATOM 1074 CD1 LEU A 69 5.555 -2.456 6.373 1.00 0.00 C

ATOM 1075 CD2 LEU A 69 7.101 -4.432 6.460 1.00 0.00 C

ATOM 1076 H LEU A 69 6.611 -3.333 1.707 1.00 0.00 H

ATOM 1077 HA LEU A 69 5.474 -1.582 3.736 1.00 0.00 H

ATOM 1078 1HB LEU A 69 5.701 -3.971 4.163 1.00 0.00 H

ATOM 1079 2HB LEU A 69 7.446 -3.888 3.900 1.00 0.00 H

ATOM 1080 HG LEU A 69 7.634 -2.423 5.923 1.00 0.00 H

ATOM 1081 1HD1 LEU A 69 5.708 -2.289 7.439 1.00 0.00 H

ATOM 1082 2HD1 LEU A 69 5.373 -1.501 5.880 1.00 0.00 H

ATOM 1083 3HD1 LEU A 69 4.697 -3.110 6.228 1.00 0.00 H

ATOM 1084 1HD2 LEU A 69 7.254 -4.265 7.527 1.00 0.00 H

ATOM 1085 2HD2 LEU A 69 6.264 -5.115 6.316 1.00 0.00 H

ATOM 1086 3HD2 LEU A 69 8.002 -4.866 6.027 1.00 0.00 H

ATOM 1087 N ASP A 70 7.305 -0.068 4.426 1.00 0.00 N

ATOM 1088 CA ASP A 70 8.341 0.904 4.756 1.00 0.00 C

ATOM 1089 C ASP A 70 9.073 0.516 6.033 1.00 0.00 C

ATOM 1090 O ASP A 70 9.935 -0.319 6.001 1.00 0.00 O

ATOM 1091 OXT ASP A 70 8.789 1.044 7.073 1.00 0.00 O

ATOM 1092 CB ASP A 70 7.735 2.301 4.912 1.00 0.00 C

ATOM 1093 CG ASP A 70 8.783 3.376 5.168 1.00 0.00 C

ATOM 1094 OD1 ASP A 70 9.895 3.029 5.491 1.00 0.00 O

ATOM 1095 OD2 ASP A 70 8.463 4.533 5.037 1.00 0.00 O

ATOM 1096 H ASP A 70 6.372 0.065 4.789 1.00 0.00 H

ATOM 1097 HA ASP A 70 9.065 0.928 3.941 1.00 0.00 H

ATOM 1098 1HB ASP A 70 7.182 2.561 4.010 1.00 0.00 H

ATOM 1099 2HB ASP A 70 7.027 2.300 5.742 1.00 0.00 H

TER
