# Supplementary material for: Biophysical characterization of the calmodulin-like domain of Plasmodium falciparum calcium dependent protein kinase 3
Source: PLoS One. 2017 Jul 26;12(7):e0181721. doi: 10.1371/journal.pone.0181721 (PMC5528832; doi:10.1371/journal.pone.0181721)
Supplement: S3 Table — (DOCX) [file pone.0181721.s008.docx]

**S3 Table. Coordinates for the selected CS-Rosetta model of *pf*CDPK3 CLD C-lobe^Ca^.**

ATOM 1 N GLN A 1 0.000 0.000 0.000 1.00 0.00 N

ATOM 2 CA GLN A 1 1.458 0.000 0.000 1.00 0.00 C

ATOM 3 C GLN A 1 2.009 1.420 0.000 1.00 0.00 C

ATOM 4 O GLN A 1 2.486 1.911 1.023 1.00 0.00 O

ATOM 5 CB GLN A 1 1.994 -0.768 -1.211 1.00 0.00 C

ATOM 6 CG GLN A 1 1.698 -2.258 -1.184 1.00 0.00 C

ATOM 7 CD GLN A 1 2.370 -2.961 -0.020 1.00 0.00 C

ATOM 8 OE1 GLN A 1 3.569 -2.791 0.216 1.00 0.00 O

ATOM 9 NE2 GLN A 1 1.601 -3.756 0.715 1.00 0.00 N

ATOM 10 1H GLN A 1 -0.334 -0.943 -0.000 1.00 0.00 H

ATOM 11 2H GLN A 1 -0.334 0.471 0.816 1.00 0.00 H

ATOM 12 3H GLN A 1 -0.334 0.471 -0.816 1.00 0.00 H

ATOM 13 HA GLN A 1 1.804 -0.495 0.907 1.00 0.00 H

ATOM 14 1HB GLN A 1 1.563 -0.356 -2.123 1.00 0.00 H

ATOM 15 2HB GLN A 1 3.075 -0.642 -1.273 1.00 0.00 H

ATOM 16 1HG GLN A 1 0.621 -2.402 -1.094 1.00 0.00 H

ATOM 17 2HG GLN A 1 2.059 -2.707 -2.109 1.00 0.00 H

ATOM 18 1HE2 GLN A 1 1.989 -4.246 1.496 1.00 0.00 H

ATOM 19 2HE2 GLN A 1 0.633 -3.865 0.488 1.00 0.00 H

ATOM 20 N LEU A 2 1.940 2.076 -1.154 1.00 0.00 N

ATOM 21 CA LEU A 2 2.502 3.412 -1.310 1.00 0.00 C

ATOM 22 C LEU A 2 1.847 4.401 -0.354 1.00 0.00 C

ATOM 23 O LEU A 2 2.531 5.137 0.358 1.00 0.00 O

ATOM 24 CB LEU A 2 2.326 3.896 -2.755 1.00 0.00 C

ATOM 25 CG LEU A 2 2.782 5.333 -3.036 1.00 0.00 C

ATOM 26 CD1 LEU A 2 4.270 5.462 -2.737 1.00 0.00 C

ATOM 27 CD2 LEU A 2 2.483 5.687 -4.485 1.00 0.00 C

ATOM 28 H LEU A 2 1.487 1.638 -1.943 1.00 0.00 H

ATOM 29 HA LEU A 2 3.567 3.368 -1.082 1.00 0.00 H

ATOM 30 1HB LEU A 2 2.888 3.235 -3.413 1.00 0.00 H

ATOM 31 2HB LEU A 2 1.271 3.825 -3.019 1.00 0.00 H

ATOM 32 HG LEU A 2 2.250 6.019 -2.377 1.00 0.00 H

ATOM 33 1HD1 LEU A 2 4.594 6.483 -2.936 1.00 0.00 H

ATOM 34 2HD1 LEU A 2 4.453 5.222 -1.689 1.00 0.00 H

ATOM 35 3HD1 LEU A 2 4.829 4.774 -3.370 1.00 0.00 H

ATOM 36 1HD2 LEU A 2 2.807 6.709 -4.684 1.00 0.00 H

ATOM 37 2HD2 LEU A 2 3.017 5.002 -5.144 1.00 0.00 H

ATOM 38 3HD2 LEU A 2 1.411 5.604 -4.666 1.00 0.00 H

ATOM 39 N SER A 3 0.519 4.412 -0.341 1.00 0.00 N

ATOM 40 CA SER A 3 -0.232 5.363 0.470 1.00 0.00 C

ATOM 41 C SER A 3 0.085 5.195 1.950 1.00 0.00 C

ATOM 42 O SER A 3 0.098 6.167 2.706 1.00 0.00 O

ATOM 43 CB SER A 3 -1.720 5.186 0.239 1.00 0.00 C

ATOM 44 OG SER A 3 -2.156 3.940 0.709 1.00 0.00 O

ATOM 45 H SER A 3 0.015 3.745 -0.908 1.00 0.00 H

ATOM 46 HA SER A 3 0.052 6.373 0.171 1.00 0.00 H

ATOM 47 1HB SER A 3 -2.264 5.980 0.749 1.00 0.00 H

ATOM 48 2HB SER A 3 -1.936 5.272 -0.825 1.00 0.00 H

ATOM 49 HG SER A 3 -2.594 4.111 1.546 1.00 0.00 H

ATOM 50 N LYS A 4 0.342 3.957 2.359 1.00 0.00 N

ATOM 51 CA LYS A 4 0.761 3.673 3.726 1.00 0.00 C

ATOM 52 C LYS A 4 2.128 4.277 4.020 1.00 0.00 C

ATOM 53 O LYS A 4 2.342 4.866 5.080 1.00 0.00 O

ATOM 54 CB LYS A 4 0.789 2.164 3.975 1.00 0.00 C

ATOM 55 CG LYS A 4 1.151 1.768 5.400 1.00 0.00 C

ATOM 56 CD LYS A 4 1.067 0.261 5.593 1.00 0.00 C

ATOM 57 CE LYS A 4 1.469 -0.140 7.005 1.00 0.00 C

ATOM 58 NZ LYS A 4 1.326 -1.604 7.229 1.00 0.00 N

ATOM 59 H LYS A 4 0.243 3.193 1.705 1.00 0.00 H

ATOM 60 HA LYS A 4 0.042 4.125 4.410 1.00 0.00 H

ATOM 61 1HB LYS A 4 -0.190 1.739 3.748 1.00 0.00 H

ATOM 62 2HB LYS A 4 1.511 1.697 3.304 1.00 0.00 H

ATOM 63 1HG LYS A 4 2.167 2.098 5.622 1.00 0.00 H

ATOM 64 2HG LYS A 4 0.469 2.253 6.097 1.00 0.00 H

ATOM 65 1HD LYS A 4 0.046 -0.074 5.406 1.00 0.00 H

ATOM 66 2HD LYS A 4 1.729 -0.234 4.883 1.00 0.00 H

ATOM 67 1HE LYS A 4 2.506 0.142 7.182 1.00 0.00 H

ATOM 68 2HE LYS A 4 0.844 0.387 7.725 1.00 0.00 H

ATOM 69 1HZ LYS A 4 1.601 -1.829 8.175 1.00 0.00 H

ATOM 70 2HZ LYS A 4 0.363 -1.875 7.086 1.00 0.00 H

ATOM 71 3HZ LYS A 4 1.916 -2.104 6.580 1.00 0.00 H

ATOM 72 N LYS A 5 3.051 4.128 3.075 1.00 0.00 N

ATOM 73 CA LYS A 5 4.382 4.709 3.206 1.00 0.00 C

ATOM 74 C LYS A 5 4.318 6.230 3.242 1.00 0.00 C

ATOM 75 O LYS A 5 5.090 6.877 3.950 1.00 0.00 O

ATOM 76 CB LYS A 5 5.283 4.247 2.060 1.00 0.00 C

ATOM 77 CG LYS A 5 5.660 2.772 2.111 1.00 0.00 C

ATOM 78 CD LYS A 5 6.512 2.378 0.914 1.00 0.00 C

ATOM 79 CE LYS A 5 6.868 0.899 0.950 1.00 0.00 C

ATOM 80 NZ LYS A 5 7.676 0.492 -0.232 1.00 0.00 N

ATOM 81 H LYS A 5 2.824 3.600 2.245 1.00 0.00 H

ATOM 82 HA LYS A 5 4.815 4.371 4.148 1.00 0.00 H

ATOM 83 1HB LYS A 5 4.784 4.432 1.108 1.00 0.00 H

ATOM 84 2HB LYS A 5 6.205 4.829 2.065 1.00 0.00 H

ATOM 85 1HG LYS A 5 6.218 2.571 3.026 1.00 0.00 H

ATOM 86 2HG LYS A 5 4.755 2.166 2.118 1.00 0.00 H

ATOM 87 1HD LYS A 5 5.966 2.590 -0.007 1.00 0.00 H

ATOM 88 2HD LYS A 5 7.431 2.963 0.913 1.00 0.00 H

ATOM 89 1HE LYS A 5 7.436 0.684 1.854 1.00 0.00 H

ATOM 90 2HE LYS A 5 5.954 0.305 0.971 1.00 0.00 H

ATOM 91 1HZ LYS A 5 7.890 -0.493 -0.170 1.00 0.00 H

ATOM 92 2HZ LYS A 5 7.151 0.670 -1.076 1.00 0.00 H

ATOM 93 3HZ LYS A 5 8.535 1.023 -0.252 1.00 0.00 H

ATOM 94 N LEU A 6 3.393 6.797 2.474 1.00 0.00 N

ATOM 95 CA LEU A 6 3.207 8.243 2.440 1.00 0.00 C

ATOM 96 C LEU A 6 2.697 8.765 3.777 1.00 0.00 C

ATOM 97 O LEU A 6 3.088 9.844 4.222 1.00 0.00 O

ATOM 98 CB LEU A 6 2.223 8.624 1.326 1.00 0.00 C

ATOM 99 CG LEU A 6 2.725 8.408 -0.107 1.00 0.00 C

ATOM 100 CD1 LEU A 6 1.592 8.678 -1.089 1.00 0.00 C

ATOM 101 CD2 LEU A 6 3.910 9.324 -0.374 1.00 0.00 C

ATOM 102 H LEU A 6 2.804 6.211 1.900 1.00 0.00 H

ATOM 103 HA LEU A 6 4.169 8.710 2.233 1.00 0.00 H

ATOM 104 1HB LEU A 6 1.314 8.038 1.450 1.00 0.00 H

ATOM 105 2HB LEU A 6 1.969 9.678 1.433 1.00 0.00 H

ATOM 106 HG LEU A 6 3.034 7.369 -0.232 1.00 0.00 H

ATOM 107 1HD1 LEU A 6 1.949 8.524 -2.107 1.00 0.00 H

ATOM 108 2HD1 LEU A 6 0.766 7.996 -0.887 1.00 0.00 H

ATOM 109 3HD1 LEU A 6 1.251 9.706 -0.975 1.00 0.00 H

ATOM 110 1HD2 LEU A 6 4.267 9.169 -1.393 1.00 0.00 H

ATOM 111 2HD2 LEU A 6 3.602 10.362 -0.251 1.00 0.00 H

ATOM 112 3HD2 LEU A 6 4.711 9.097 0.329 1.00 0.00 H

ATOM 113 N ILE A 7 1.822 7.993 4.413 1.00 0.00 N

ATOM 114 CA ILE A 7 1.356 8.311 5.757 1.00 0.00 C

ATOM 115 C ILE A 7 2.500 8.270 6.761 1.00 0.00 C

ATOM 116 O ILE A 7 2.636 9.163 7.598 1.00 0.00 O

ATOM 117 CB ILE A 7 0.249 7.337 6.199 1.00 0.00 C

ATOM 118 CG1 ILE A 7 -1.012 7.543 5.355 1.00 0.00 C

ATOM 119 CG2 ILE A 7 -0.060 7.518 7.677 1.00 0.00 C

ATOM 120 CD1 ILE A 7 -2.028 6.432 5.494 1.00 0.00 C

ATOM 121 H ILE A 7 1.471 7.165 3.953 1.00 0.00 H

ATOM 122 HA ILE A 7 0.944 9.320 5.750 1.00 0.00 H

ATOM 123 HB ILE A 7 0.577 6.312 6.029 1.00 0.00 H

ATOM 124 1HG1 ILE A 7 -1.490 8.480 5.639 1.00 0.00 H

ATOM 125 2HG1 ILE A 7 -0.737 7.623 4.303 1.00 0.00 H

ATOM 126 1HG2 ILE A 7 -0.845 6.822 7.972 1.00 0.00 H

ATOM 127 2HG2 ILE A 7 0.837 7.322 8.262 1.00 0.00 H

ATOM 128 3HG2 ILE A 7 -0.395 8.540 7.856 1.00 0.00 H

ATOM 129 1HD1 ILE A 7 -2.893 6.649 4.866 1.00 0.00 H

ATOM 130 2HD1 ILE A 7 -1.580 5.488 5.180 1.00 0.00 H

ATOM 131 3HD1 ILE A 7 -2.345 6.356 6.533 1.00 0.00 H

ATOM 132 N TYR A 8 3.321 7.229 6.673 1.00 0.00 N

ATOM 133 CA TYR A 8 4.521 7.128 7.494 1.00 0.00 C

ATOM 134 C TYR A 8 5.405 8.359 7.332 1.00 0.00 C

ATOM 135 O TYR A 8 5.857 8.944 8.316 1.00 0.00 O

ATOM 136 CB TYR A 8 5.305 5.862 7.143 1.00 0.00 C

ATOM 137 CG TYR A 8 6.625 5.739 7.872 1.00 0.00 C

ATOM 138 CD1 TYR A 8 6.692 5.048 9.073 1.00 0.00 C

ATOM 139 CD2 TYR A 8 7.768 6.318 7.341 1.00 0.00 C

ATOM 140 CE1 TYR A 8 7.897 4.935 9.739 1.00 0.00 C

ATOM 141 CE2 TYR A 8 8.973 6.205 8.007 1.00 0.00 C

ATOM 142 CZ TYR A 8 9.039 5.518 9.201 1.00 0.00 C

ATOM 143 OH TYR A 8 10.240 5.406 9.864 1.00 0.00 O

ATOM 144 H TYR A 8 3.108 6.489 6.020 1.00 0.00 H

ATOM 145 HA TYR A 8 4.221 7.073 8.541 1.00 0.00 H

ATOM 146 1HB TYR A 8 4.703 4.984 7.379 1.00 0.00 H

ATOM 147 2HB TYR A 8 5.506 5.844 6.072 1.00 0.00 H

ATOM 148 HD1 TYR A 8 5.794 4.593 9.491 1.00 0.00 H

ATOM 149 HD2 TYR A 8 7.715 6.862 6.398 1.00 0.00 H

ATOM 150 HE1 TYR A 8 7.950 4.392 10.682 1.00 0.00 H

ATOM 151 HE2 TYR A 8 9.871 6.661 7.589 1.00 0.00 H

ATOM 152 HH TYR A 8 10.619 4.540 9.694 1.00 0.00 H

ATOM 153 N CYS A 9 5.647 8.747 6.085 1.00 0.00 N

ATOM 154 CA CYS A 9 6.481 9.906 5.791 1.00 0.00 C

ATOM 155 C CYS A 9 5.884 11.177 6.383 1.00 0.00 C

ATOM 156 O CYS A 9 6.593 11.988 6.978 1.00 0.00 O

ATOM 157 CB CYS A 9 6.646 10.081 4.282 1.00 0.00 C

ATOM 158 SG CYS A 9 7.672 8.814 3.499 1.00 0.00 S

ATOM 159 H CYS A 9 5.243 8.225 5.320 1.00 0.00 H

ATOM 160 HA CYS A 9 7.464 9.746 6.232 1.00 0.00 H

ATOM 161 1HB CYS A 9 5.666 10.065 3.804 1.00 0.00 H

ATOM 162 2HB CYS A 9 7.095 11.053 4.075 1.00 0.00 H

ATOM 163 HG CYS A 9 7.464 9.218 2.250 1.00 0.00 H

ATOM 164 N ALA A 10 4.577 11.345 6.214 1.00 0.00 N

ATOM 165 CA ALA A 10 3.874 12.494 6.773 1.00 0.00 C

ATOM 166 C ALA A 10 4.048 12.564 8.285 1.00 0.00 C

ATOM 167 O ALA A 10 4.303 13.632 8.841 1.00 0.00 O

ATOM 168 CB ALA A 10 2.397 12.437 6.413 1.00 0.00 C

ATOM 169 H ALA A 10 4.057 10.660 5.686 1.00 0.00 H

ATOM 170 HA ALA A 10 4.308 13.397 6.345 1.00 0.00 H

ATOM 171 1HB ALA A 10 1.885 13.301 6.838 1.00 0.00 H

ATOM 172 2HB ALA A 10 2.286 12.446 5.329 1.00 0.00 H

ATOM 173 3HB ALA A 10 1.959 11.524 6.814 1.00 0.00 H

ATOM 174 N PHE A 11 3.910 11.419 8.945 1.00 0.00 N

ATOM 175 CA PHE A 11 4.153 11.328 10.380 1.00 0.00 C

ATOM 176 C PHE A 11 5.564 11.781 10.729 1.00 0.00 C

ATOM 177 O PHE A 11 5.768 12.527 11.687 1.00 0.00 O

ATOM 178 CB PHE A 11 3.935 9.895 10.869 1.00 0.00 C

ATOM 179 CG PHE A 11 4.069 9.735 12.356 1.00 0.00 C

ATOM 180 CD1 PHE A 11 2.944 9.679 13.166 1.00 0.00 C

ATOM 181 CD2 PHE A 11 5.319 9.642 12.949 1.00 0.00 C

ATOM 182 CE1 PHE A 11 3.066 9.532 14.535 1.00 0.00 C

ATOM 183 CE2 PHE A 11 5.444 9.493 14.317 1.00 0.00 C

ATOM 184 CZ PHE A 11 4.315 9.439 15.110 1.00 0.00 C

ATOM 185 H PHE A 11 3.629 10.591 8.440 1.00 0.00 H

ATOM 186 HA PHE A 11 3.446 11.982 10.893 1.00 0.00 H

ATOM 187 1HB PHE A 11 2.941 9.558 10.578 1.00 0.00 H

ATOM 188 2HB PHE A 11 4.657 9.234 10.390 1.00 0.00 H

ATOM 189 HD1 PHE A 11 1.956 9.751 12.711 1.00 0.00 H

ATOM 190 HD2 PHE A 11 6.210 9.685 12.322 1.00 0.00 H

ATOM 191 HE1 PHE A 11 2.174 9.489 15.159 1.00 0.00 H

ATOM 192 HE2 PHE A 11 6.432 9.421 14.770 1.00 0.00 H

ATOM 193 HZ PHE A 11 4.412 9.324 16.189 1.00 0.00 H

ATOM 194 N ARG A 12 6.537 11.325 9.947 1.00 0.00 N

ATOM 195 CA ARG A 12 7.935 11.666 10.185 1.00 0.00 C

ATOM 196 C ARG A 12 8.158 13.170 10.093 1.00 0.00 C

ATOM 197 O ARG A 12 8.957 13.735 10.840 1.00 0.00 O

ATOM 198 CB ARG A 12 8.836 10.960 9.182 1.00 0.00 C

ATOM 199 CG ARG A 12 10.320 11.243 9.347 1.00 0.00 C

ATOM 200 CD ARG A 12 10.828 10.768 10.660 1.00 0.00 C

ATOM 201 NE ARG A 12 10.750 9.321 10.780 1.00 0.00 N

ATOM 202 CZ ARG A 12 11.023 8.632 11.905 1.00 0.00 C

ATOM 203 NH1 ARG A 12 11.390 9.269 12.994 1.00 0.00 N

ATOM 204 NH2 ARG A 12 10.922 7.314 11.913 1.00 0.00 N

ATOM 205 H ARG A 12 6.302 10.727 9.168 1.00 0.00 H

ATOM 206 HA ARG A 12 8.205 11.335 11.188 1.00 0.00 H

ATOM 207 1HB ARG A 12 8.695 9.883 9.261 1.00 0.00 H

ATOM 208 2HB ARG A 12 8.554 11.254 8.171 1.00 0.00 H

ATOM 209 1HG ARG A 12 10.877 10.734 8.560 1.00 0.00 H

ATOM 210 2HG ARG A 12 10.496 12.317 9.279 1.00 0.00 H

ATOM 211 1HD ARG A 12 11.870 11.064 10.776 1.00 0.00 H

ATOM 212 2HD ARG A 12 10.234 11.209 11.460 1.00 0.00 H

ATOM 213 HE ARG A 12 10.471 8.796 9.962 1.00 0.00 H

ATOM 214 1HH1 ARG A 12 11.467 10.277 12.988 1.00 0.00 H

ATOM 215 2HH1 ARG A 12 11.595 8.752 13.837 1.00 0.00 H

ATOM 216 1HH2 ARG A 12 10.640 6.823 11.075 1.00 0.00 H

ATOM 217 2HH2 ARG A 12 11.127 6.797 12.755 1.00 0.00 H

ATOM 218 N VAL A 13 7.447 13.813 9.174 1.00 0.00 N

ATOM 219 CA VAL A 13 7.496 15.265 9.045 1.00 0.00 C

ATOM 220 C VAL A 13 7.088 15.947 10.344 1.00 0.00 C

ATOM 221 O VAL A 13 7.719 16.912 10.775 1.00 0.00 O

ATOM 222 CB VAL A 13 6.567 15.732 7.909 1.00 0.00 C

ATOM 223 CG1 VAL A 13 6.417 17.246 7.932 1.00 0.00 C

ATOM 224 CG2 VAL A 13 7.113 15.265 6.568 1.00 0.00 C

ATOM 225 H VAL A 13 6.858 13.284 8.547 1.00 0.00 H

ATOM 226 HA VAL A 13 8.519 15.557 8.803 1.00 0.00 H

ATOM 227 HB VAL A 13 5.575 15.309 8.066 1.00 0.00 H

ATOM 228 1HG1 VAL A 13 5.757 17.559 7.123 1.00 0.00 H

ATOM 229 2HG1 VAL A 13 5.991 17.555 8.887 1.00 0.00 H

ATOM 230 3HG1 VAL A 13 7.395 17.711 7.802 1.00 0.00 H

ATOM 231 1HG2 VAL A 13 6.451 15.598 5.770 1.00 0.00 H

ATOM 232 2HG2 VAL A 13 8.108 15.684 6.414 1.00 0.00 H

ATOM 233 3HG2 VAL A 13 7.173 14.176 6.559 1.00 0.00 H

ATOM 234 N PHE A 14 6.028 15.440 10.965 1.00 0.00 N

ATOM 235 CA PHE A 14 5.568 15.962 12.246 1.00 0.00 C

ATOM 236 C PHE A 14 6.533 15.597 13.368 1.00 0.00 C

ATOM 237 O PHE A 14 6.768 16.390 14.279 1.00 0.00 O

ATOM 238 CB PHE A 14 4.173 15.425 12.572 1.00 0.00 C

ATOM 239 CG PHE A 14 3.077 16.056 11.762 1.00 0.00 C

ATOM 240 CD1 PHE A 14 2.514 15.383 10.688 1.00 0.00 C

ATOM 241 CD2 PHE A 14 2.607 17.323 12.071 1.00 0.00 C

ATOM 242 CE1 PHE A 14 1.505 15.962 9.941 1.00 0.00 C

ATOM 243 CE2 PHE A 14 1.598 17.904 11.327 1.00 0.00 C

ATOM 244 CZ PHE A 14 1.047 17.222 10.261 1.00 0.00 C

ATOM 245 H PHE A 14 5.528 14.673 10.538 1.00 0.00 H

ATOM 246 HA PHE A 14 5.517 17.049 12.179 1.00 0.00 H

ATOM 247 1HB PHE A 14 4.148 14.350 12.400 1.00 0.00 H

ATOM 248 2HB PHE A 14 3.956 15.593 13.626 1.00 0.00 H

ATOM 249 HD1 PHE A 14 2.875 14.385 10.436 1.00 0.00 H

ATOM 250 HD2 PHE A 14 3.042 17.862 12.913 1.00 0.00 H

ATOM 251 HE1 PHE A 14 1.072 15.422 9.100 1.00 0.00 H

ATOM 252 HE2 PHE A 14 1.237 18.901 11.581 1.00 0.00 H

ATOM 253 HZ PHE A 14 0.253 17.680 9.672 1.00 0.00 H

ATOM 254 N ASP A 15 7.089 14.393 13.294 1.00 0.00 N

ATOM 255 CA ASP A 15 8.012 13.912 14.316 1.00 0.00 C

ATOM 256 C ASP A 15 9.384 14.554 14.165 1.00 0.00 C

ATOM 257 O ASP A 15 10.306 13.950 13.617 1.00 0.00 O

ATOM 258 CB ASP A 15 8.144 12.389 14.245 1.00 0.00 C

ATOM 259 CG ASP A 15 8.933 11.808 15.411 1.00 0.00 C

ATOM 260 OD1 ASP A 15 9.339 12.562 16.263 1.00 0.00 O

ATOM 261 OD2 ASP A 15 9.122 10.615 15.438 1.00 0.00 O

ATOM 262 H ASP A 15 6.867 13.795 12.511 1.00 0.00 H

ATOM 263 HA ASP A 15 7.615 14.182 15.295 1.00 0.00 H

ATOM 264 1HB ASP A 15 7.151 11.937 14.236 1.00 0.00 H

ATOM 265 2HB ASP A 15 8.639 12.109 13.315 1.00 0.00 H

ATOM 266 N VAL A 16 9.514 15.783 14.654 1.00 0.00 N

ATOM 267 CA VAL A 16 10.721 16.572 14.434 1.00 0.00 C

ATOM 268 C VAL A 16 11.909 15.980 15.181 1.00 0.00 C

ATOM 269 O VAL A 16 12.989 15.811 14.615 1.00 0.00 O

ATOM 270 CB VAL A 16 10.500 18.025 14.893 1.00 0.00 C

ATOM 271 CG1 VAL A 16 11.804 18.807 14.832 1.00 0.00 C

ATOM 272 CG2 VAL A 16 9.435 18.685 14.031 1.00 0.00 C

ATOM 273 H VAL A 16 8.758 16.181 15.192 1.00 0.00 H

ATOM 274 HA VAL A 16 10.944 16.572 13.366 1.00 0.00 H

ATOM 275 HB VAL A 16 10.176 18.022 15.934 1.00 0.00 H

ATOM 276 1HG1 VAL A 16 11.630 19.832 15.160 1.00 0.00 H

ATOM 277 2HG1 VAL A 16 12.541 18.337 15.484 1.00 0.00 H

ATOM 278 3HG1 VAL A 16 12.177 18.813 13.808 1.00 0.00 H

ATOM 279 1HG2 VAL A 16 9.284 19.712 14.362 1.00 0.00 H

ATOM 280 2HG2 VAL A 16 9.757 18.682 12.989 1.00 0.00 H

ATOM 281 3HG2 VAL A 16 8.499 18.133 14.123 1.00 0.00 H

ATOM 282 N ASP A 17 11.704 15.668 16.456 1.00 0.00 N

ATOM 283 CA ASP A 17 12.788 15.215 17.319 1.00 0.00 C

ATOM 284 C ASP A 17 12.981 13.708 17.216 1.00 0.00 C

ATOM 285 O ASP A 17 13.872 13.142 17.850 1.00 0.00 O

ATOM 286 CB ASP A 17 12.514 15.603 18.774 1.00 0.00 C

ATOM 287 CG ASP A 17 11.333 14.854 19.374 1.00 0.00 C

ATOM 288 OD1 ASP A 17 10.834 13.959 18.733 1.00 0.00 O

ATOM 289 OD2 ASP A 17 10.940 15.183 20.468 1.00 0.00 O

ATOM 290 H ASP A 17 10.772 15.747 16.838 1.00 0.00 H

ATOM 291 HA ASP A 17 13.711 15.699 16.997 1.00 0.00 H

ATOM 292 1HB ASP A 17 13.399 15.400 19.378 1.00 0.00 H

ATOM 293 2HB ASP A 17 12.316 16.673 18.834 1.00 0.00 H

ATOM 294 N ASN A 18 12.140 13.062 16.415 1.00 0.00 N

ATOM 295 CA ASN A 18 12.330 11.656 16.078 1.00 0.00 C

ATOM 296 C ASN A 18 12.320 10.785 17.328 1.00 0.00 C

ATOM 297 O ASN A 18 13.312 10.130 17.647 1.00 0.00 O

ATOM 298 CB ASN A 18 13.619 11.462 15.302 1.00 0.00 C

ATOM 299 CG ASN A 18 13.588 12.128 13.954 1.00 0.00 C

ATOM 300 OD1 ASN A 18 12.686 11.881 13.146 1.00 0.00 O

ATOM 301 ND2 ASN A 18 14.556 12.970 13.696 1.00 0.00 N

ATOM 302 H ASN A 18 11.350 13.559 16.028 1.00 0.00 H

ATOM 303 HA ASN A 18 11.495 11.334 15.453 1.00 0.00 H

ATOM 304 1HB ASN A 18 14.454 11.866 15.875 1.00 0.00 H

ATOM 305 2HB ASN A 18 13.803 10.396 15.163 1.00 0.00 H

ATOM 306 1HD2 ASN A 18 14.587 13.443 12.815 1.00 0.00 H

ATOM 307 2HD2 ASN A 18 15.266 13.140 14.380 1.00 0.00 H

ATOM 308 N ASP A 19 11.193 10.782 18.032 1.00 0.00 N

ATOM 309 CA ASP A 19 11.007 9.895 19.174 1.00 0.00 C

ATOM 310 C ASP A 19 9.805 8.982 18.972 1.00 0.00 C

ATOM 311 O ASP A 19 9.404 8.256 19.883 1.00 0.00 O

ATOM 312 CB ASP A 19 10.827 10.707 20.460 1.00 0.00 C

ATOM 313 CG ASP A 19 9.491 11.435 20.519 1.00 0.00 C

ATOM 314 OD1 ASP A 19 8.724 11.304 19.595 1.00 0.00 O

ATOM 315 OD2 ASP A 19 9.251 12.115 21.488 1.00 0.00 O

ATOM 316 H ASP A 19 10.447 11.409 17.769 1.00 0.00 H

ATOM 317 HA ASP A 19 11.895 9.272 19.277 1.00 0.00 H

ATOM 318 1HB ASP A 19 10.902 10.045 21.322 1.00 0.00 H

ATOM 319 2HB ASP A 19 11.629 11.442 20.541 1.00 0.00 H

ATOM 320 N GLY A 20 9.233 9.021 17.774 1.00 0.00 N

ATOM 321 CA GLY A 20 8.212 8.057 17.379 1.00 0.00 C

ATOM 322 C GLY A 20 6.817 8.560 17.726 1.00 0.00 C

ATOM 323 O GLY A 20 5.821 7.886 17.462 1.00 0.00 O

ATOM 324 H GLY A 20 9.512 9.738 17.119 1.00 0.00 H

ATOM 325 1HA GLY A 20 8.281 7.873 16.307 1.00 0.00 H

ATOM 326 2HA GLY A 20 8.396 7.108 17.881 1.00 0.00 H

ATOM 327 N GLU A 21 6.751 9.747 18.320 1.00 0.00 N

ATOM 328 CA GLU A 21 5.478 10.335 18.718 1.00 0.00 C

ATOM 329 C GLU A 21 5.400 11.803 18.319 1.00 0.00 C

ATOM 330 O GLU A 21 6.415 12.499 18.278 1.00 0.00 O

ATOM 331 CB GLU A 21 5.275 10.195 20.228 1.00 0.00 C

ATOM 332 CG GLU A 21 5.280 8.760 20.734 1.00 0.00 C

ATOM 333 CD GLU A 21 4.072 7.980 20.293 1.00 0.00 C

ATOM 334 OE1 GLU A 21 3.089 8.590 19.946 1.00 0.00 O

ATOM 335 OE2 GLU A 21 4.132 6.773 20.303 1.00 0.00 O

ATOM 336 H GLU A 21 7.605 10.254 18.500 1.00 0.00 H

ATOM 337 HA GLU A 21 4.675 9.801 18.207 1.00 0.00 H

ATOM 338 1HB GLU A 21 6.062 10.737 20.753 1.00 0.00 H

ATOM 339 2HB GLU A 21 4.323 10.646 20.511 1.00 0.00 H

ATOM 340 1HG GLU A 21 6.175 8.259 20.366 1.00 0.00 H

ATOM 341 2HG GLU A 21 5.323 8.769 21.822 1.00 0.00 H

ATOM 342 N ILE A 22 4.191 12.268 18.025 1.00 0.00 N

ATOM 343 CA ILE A 22 3.966 13.673 17.709 1.00 0.00 C

ATOM 344 C ILE A 22 3.505 14.448 18.937 1.00 0.00 C

ATOM 345 O ILE A 22 2.464 14.143 19.519 1.00 0.00 O

ATOM 346 CB ILE A 22 2.925 13.824 16.585 1.00 0.00 C

ATOM 347 CG1 ILE A 22 3.409 13.126 15.311 1.00 0.00 C

ATOM 348 CG2 ILE A 22 2.641 15.293 16.316 1.00 0.00 C

ATOM 349 CD1 ILE A 22 2.348 13.002 14.241 1.00 0.00 C

ATOM 350 H ILE A 22 3.407 11.631 18.021 1.00 0.00 H

ATOM 351 HA ILE A 22 4.906 14.105 17.367 1.00 0.00 H

ATOM 352 HB ILE A 22 1.998 13.333 16.880 1.00 0.00 H

ATOM 353 1HG1 ILE A 22 4.253 13.675 14.894 1.00 0.00 H

ATOM 354 2HG1 ILE A 22 3.763 12.124 15.557 1.00 0.00 H

ATOM 355 1HG2 ILE A 22 1.904 15.381 15.518 1.00 0.00 H

ATOM 356 2HG2 ILE A 22 2.255 15.761 17.220 1.00 0.00 H

ATOM 357 3HG2 ILE A 22 3.563 15.792 16.014 1.00 0.00 H

ATOM 358 1HD1 ILE A 22 2.767 12.497 13.370 1.00 0.00 H

ATOM 359 2HD1 ILE A 22 1.508 12.424 14.627 1.00 0.00 H

ATOM 360 3HD1 ILE A 22 2.005 13.995 13.954 1.00 0.00 H

ATOM 361 N THR A 23 4.286 15.450 19.326 1.00 0.00 N

ATOM 362 CA THR A 23 3.946 16.285 20.471 1.00 0.00 C

ATOM 363 C THR A 23 3.088 17.472 20.053 1.00 0.00 C

ATOM 364 O THR A 23 2.907 17.731 18.863 1.00 0.00 O

ATOM 365 CB THR A 23 5.214 16.790 21.185 1.00 0.00 C

ATOM 366 OG1 THR A 23 5.929 17.682 20.320 1.00 0.00 O

ATOM 367 CG2 THR A 23 6.115 15.623 21.560 1.00 0.00 C

ATOM 368 H THR A 23 5.136 15.638 18.814 1.00 0.00 H

ATOM 369 HA THR A 23 3.371 15.686 21.178 1.00 0.00 H

ATOM 370 HB THR A 23 4.933 17.330 22.089 1.00 0.00 H

ATOM 371 HG1 THR A 23 6.744 17.264 20.033 1.00 0.00 H

ATOM 372 1HG2 THR A 23 7.006 15.999 22.063 1.00 0.00 H

ATOM 373 2HG2 THR A 23 5.578 14.949 22.227 1.00 0.00 H

ATOM 374 3HG2 THR A 23 6.408 15.085 20.659 1.00 0.00 H

ATOM 375 N THR A 24 2.561 18.191 21.038 1.00 0.00 N

ATOM 376 CA THR A 24 1.769 19.387 20.775 1.00 0.00 C

ATOM 377 C THR A 24 2.598 20.451 20.066 1.00 0.00 C

ATOM 378 O THR A 24 2.130 21.090 19.124 1.00 0.00 O

ATOM 379 CB THR A 24 1.190 19.966 22.079 1.00 0.00 C

ATOM 380 OG1 THR A 24 0.343 18.992 22.702 1.00 0.00 O

ATOM 381 CG2 THR A 24 0.384 21.224 21.792 1.00 0.00 C

ATOM 382 H THR A 24 2.712 17.902 21.994 1.00 0.00 H

ATOM 383 HA THR A 24 0.940 19.116 20.121 1.00 0.00 H

ATOM 384 HB THR A 24 2.003 20.211 22.761 1.00 0.00 H

ATOM 385 HG1 THR A 24 0.403 19.080 23.657 1.00 0.00 H

ATOM 386 1HG2 THR A 24 -0.018 21.619 22.725 1.00 0.00 H

ATOM 387 2HG2 THR A 24 1.028 21.971 21.328 1.00 0.00 H

ATOM 388 3HG2 THR A 24 -0.437 20.984 21.117 1.00 0.00 H

ATOM 389 N ALA A 25 3.830 20.637 20.526 1.00 0.00 N

ATOM 390 CA ALA A 25 4.734 21.609 19.922 1.00 0.00 C

ATOM 391 C ALA A 25 5.059 21.239 18.481 1.00 0.00 C

ATOM 392 O ALA A 25 5.071 22.096 17.597 1.00 0.00 O

ATOM 393 CB ALA A 25 6.012 21.723 20.741 1.00 0.00 C

ATOM 394 H ALA A 25 4.150 20.091 21.313 1.00 0.00 H

ATOM 395 HA ALA A 25 4.233 22.577 19.914 1.00 0.00 H

ATOM 396 1HB ALA A 25 6.677 22.453 20.278 1.00 0.00 H

ATOM 397 2HB ALA A 25 5.769 22.045 21.753 1.00 0.00 H

ATOM 398 3HB ALA A 25 6.508 20.754 20.778 1.00 0.00 H

ATOM 399 N GLU A 26 5.324 19.957 18.250 1.00 0.00 N

ATOM 400 CA GLU A 26 5.645 19.470 16.914 1.00 0.00 C

ATOM 401 C GLU A 26 4.465 19.644 15.966 1.00 0.00 C

ATOM 402 O GLU A 26 4.624 20.119 14.842 1.00 0.00 O

ATOM 403 CB GLU A 26 6.055 17.997 16.968 1.00 0.00 C

ATOM 404 CG GLU A 26 7.459 17.755 17.504 1.00 0.00 C

ATOM 405 CD GLU A 26 7.792 16.295 17.634 1.00 0.00 C

ATOM 406 OE1 GLU A 26 6.925 15.537 17.999 1.00 0.00 O

ATOM 407 OE2 GLU A 26 8.915 15.936 17.368 1.00 0.00 O

ATOM 408 H GLU A 26 5.302 19.304 19.020 1.00 0.00 H

ATOM 409 HA GLU A 26 6.481 20.052 16.525 1.00 0.00 H

ATOM 410 1HB GLU A 26 5.357 17.447 17.599 1.00 0.00 H

ATOM 411 2HB GLU A 26 6.001 17.567 15.967 1.00 0.00 H

ATOM 412 1HG GLU A 26 8.179 18.221 16.831 1.00 0.00 H

ATOM 413 2HG GLU A 26 7.552 18.232 18.478 1.00 0.00 H

ATOM 414 N LEU A 27 3.280 19.256 16.427 1.00 0.00 N

ATOM 415 CA LEU A 27 2.073 19.357 15.616 1.00 0.00 C

ATOM 416 C LEU A 27 1.756 20.808 15.278 1.00 0.00 C

ATOM 417 O LEU A 27 1.457 21.137 14.130 1.00 0.00 O

ATOM 418 CB LEU A 27 0.885 18.726 16.355 1.00 0.00 C

ATOM 419 CG LEU A 27 -0.454 18.754 15.606 1.00 0.00 C

ATOM 420 CD1 LEU A 27 -0.290 18.088 14.247 1.00 0.00 C

ATOM 421 CD2 LEU A 27 -1.515 18.048 16.437 1.00 0.00 C

ATOM 422 H LEU A 27 3.215 18.881 17.363 1.00 0.00 H

ATOM 423 HA LEU A 27 2.235 18.815 14.686 1.00 0.00 H

ATOM 424 1HB LEU A 27 1.122 17.686 16.570 1.00 0.00 H

ATOM 425 2HB LEU A 27 0.746 19.249 17.301 1.00 0.00 H

ATOM 426 HG LEU A 27 -0.755 19.788 15.436 1.00 0.00 H

ATOM 427 1HD1 LEU A 27 -1.241 18.108 13.715 1.00 0.00 H

ATOM 428 2HD1 LEU A 27 0.460 18.625 13.666 1.00 0.00 H

ATOM 429 3HD1 LEU A 27 0.027 17.055 14.384 1.00 0.00 H

ATOM 430 1HD2 LEU A 27 -2.467 18.068 15.905 1.00 0.00 H

ATOM 431 2HD2 LEU A 27 -1.216 17.013 16.606 1.00 0.00 H

ATOM 432 3HD2 LEU A 27 -1.624 18.555 17.396 1.00 0.00 H

ATOM 433 N ALA A 28 1.824 21.673 16.285 1.00 0.00 N

ATOM 434 CA ALA A 28 1.608 23.101 16.083 1.00 0.00 C

ATOM 435 C ALA A 28 2.639 23.684 15.126 1.00 0.00 C

ATOM 436 O ALA A 28 2.307 24.492 14.258 1.00 0.00 O

ATOM 437 CB ALA A 28 1.648 23.836 17.415 1.00 0.00 C

ATOM 438 H ALA A 28 2.030 21.333 17.213 1.00 0.00 H

ATOM 439 HA ALA A 28 0.623 23.235 15.636 1.00 0.00 H

ATOM 440 1HB ALA A 28 1.485 24.900 17.248 1.00 0.00 H

ATOM 441 2HB ALA A 28 0.867 23.447 18.069 1.00 0.00 H

ATOM 442 3HB ALA A 28 2.620 23.686 17.883 1.00 0.00 H

ATOM 443 N HIS A 29 3.891 23.269 15.288 1.00 0.00 N

ATOM 444 CA HIS A 29 4.979 23.777 14.461 1.00 0.00 C

ATOM 445 C HIS A 29 4.680 23.588 12.980 1.00 0.00 C

ATOM 446 O HIS A 29 4.816 24.518 12.185 1.00 0.00 O

ATOM 447 CB HIS A 29 6.298 23.081 14.815 1.00 0.00 C

ATOM 448 CG HIS A 29 7.451 23.509 13.962 1.00 0.00 C

ATOM 449 ND1 HIS A 29 8.035 24.754 14.071 1.00 0.00 N

ATOM 450 CD2 HIS A 29 8.130 22.860 12.988 1.00 0.00 C

ATOM 451 CE1 HIS A 29 9.023 24.851 13.199 1.00 0.00 C

ATOM 452 NE2 HIS A 29 9.101 23.716 12.530 1.00 0.00 N

ATOM 453 H HIS A 29 4.094 22.585 16.003 1.00 0.00 H

ATOM 454 HA HIS A 29 5.103 24.845 14.639 1.00 0.00 H

ATOM 455 1HB HIS A 29 6.549 23.284 15.856 1.00 0.00 H

ATOM 456 2HB HIS A 29 6.179 22.003 14.711 1.00 0.00 H

ATOM 457 HD2 HIS A 29 7.940 21.846 12.632 1.00 0.00 H

ATOM 458 HE1 HIS A 29 9.665 25.720 13.057 1.00 0.00 H

ATOM 459 HE2 HIS A 29 9.765 23.505 11.799 1.00 0.00 H

ATOM 460 N ILE A 30 4.272 22.377 12.614 1.00 0.00 N

ATOM 461 CA ILE A 30 4.023 22.042 11.217 1.00 0.00 C

ATOM 462 C ILE A 30 2.759 22.721 10.705 1.00 0.00 C

ATOM 463 O ILE A 30 2.718 23.206 9.574 1.00 0.00 O

ATOM 464 CB ILE A 30 3.901 20.519 11.030 1.00 0.00 C

ATOM 465 CG1 ILE A 30 5.211 19.827 11.416 1.00 0.00 C

ATOM 466 CG2 ILE A 30 3.522 20.188 9.595 1.00 0.00 C

ATOM 467 CD1 ILE A 30 6.405 20.291 10.614 1.00 0.00 C

ATOM 468 H ILE A 30 4.130 21.671 13.322 1.00 0.00 H

ATOM 469 HA ILE A 30 4.864 22.396 10.621 1.00 0.00 H

ATOM 470 HB ILE A 30 3.131 20.132 11.698 1.00 0.00 H

ATOM 471 1HG1 ILE A 30 5.418 20.004 12.471 1.00 0.00 H

ATOM 472 2HG1 ILE A 30 5.107 18.750 11.280 1.00 0.00 H

ATOM 473 1HG2 ILE A 30 3.441 19.107 9.481 1.00 0.00 H

ATOM 474 2HG2 ILE A 30 2.566 20.651 9.355 1.00 0.00 H

ATOM 475 3HG2 ILE A 30 4.289 20.567 8.920 1.00 0.00 H

ATOM 476 1HD1 ILE A 30 7.296 19.755 10.944 1.00 0.00 H

ATOM 477 2HD1 ILE A 30 6.233 20.091 9.556 1.00 0.00 H

ATOM 478 3HD1 ILE A 30 6.550 21.360 10.763 1.00 0.00 H

ATOM 479 N LEU A 31 1.728 22.750 11.543 1.00 0.00 N

ATOM 480 CA LEU A 31 0.452 23.347 11.168 1.00 0.00 C

ATOM 481 C LEU A 31 0.606 24.833 10.870 1.00 0.00 C

ATOM 482 O LEU A 31 -0.006 25.356 9.938 1.00 0.00 O

ATOM 483 CB LEU A 31 -0.576 23.148 12.288 1.00 0.00 C

ATOM 484 CG LEU A 31 -1.082 21.712 12.479 1.00 0.00 C

ATOM 485 CD1 LEU A 31 -1.927 21.635 13.743 1.00 0.00 C

ATOM 486 CD2 LEU A 31 -1.885 21.290 11.258 1.00 0.00 C

ATOM 487 H LEU A 31 1.831 22.349 12.464 1.00 0.00 H

ATOM 488 HA LEU A 31 0.090 22.852 10.267 1.00 0.00 H

ATOM 489 1HB LEU A 31 -0.132 23.470 13.228 1.00 0.00 H

ATOM 490 2HB LEU A 31 -1.441 23.779 12.083 1.00 0.00 H

ATOM 491 HG LEU A 31 -0.232 21.041 12.604 1.00 0.00 H

ATOM 492 1HD1 LEU A 31 -2.286 20.615 13.879 1.00 0.00 H

ATOM 493 2HD1 LEU A 31 -1.322 21.923 14.603 1.00 0.00 H

ATOM 494 3HD1 LEU A 31 -2.778 22.310 13.654 1.00 0.00 H

ATOM 495 1HD2 LEU A 31 -2.244 20.270 11.393 1.00 0.00 H

ATOM 496 2HD2 LEU A 31 -2.736 21.961 11.132 1.00 0.00 H

ATOM 497 3HD2 LEU A 31 -1.252 21.338 10.371 1.00 0.00 H

ATOM 498 N TYR A 32 1.427 25.510 11.666 1.00 0.00 N

ATOM 499 CA TYR A 32 1.630 26.945 11.516 1.00 0.00 C

ATOM 500 C TYR A 32 2.977 27.247 10.871 1.00 0.00 C

ATOM 501 O TYR A 32 3.325 28.407 10.653 1.00 0.00 O

ATOM 502 CB TYR A 32 1.523 27.646 12.872 1.00 0.00 C

ATOM 503 CG TYR A 32 0.132 27.617 13.466 1.00 0.00 C

ATOM 504 CD1 TYR A 32 -0.213 26.629 14.377 1.00 0.00 C

ATOM 505 CD2 TYR A 32 -0.799 28.578 13.100 1.00 0.00 C

ATOM 506 CE1 TYR A 32 -1.483 26.603 14.919 1.00 0.00 C

ATOM 507 CE2 TYR A 32 -2.069 28.552 13.642 1.00 0.00 C

ATOM 508 CZ TYR A 32 -2.412 27.569 14.548 1.00 0.00 C

ATOM 509 OH TYR A 32 -3.677 27.543 15.088 1.00 0.00 O

ATOM 510 H TYR A 32 1.923 25.016 12.395 1.00 0.00 H

ATOM 511 HA TYR A 32 0.853 27.338 10.858 1.00 0.00 H

ATOM 512 1HB TYR A 32 2.205 27.175 13.581 1.00 0.00 H

ATOM 513 2HB TYR A 32 1.827 28.687 12.769 1.00 0.00 H

ATOM 514 HD1 TYR A 32 0.518 25.874 14.665 1.00 0.00 H

ATOM 515 HD2 TYR A 32 -0.528 29.354 12.383 1.00 0.00 H

ATOM 516 HE1 TYR A 32 -1.754 25.827 15.635 1.00 0.00 H

ATOM 517 HE2 TYR A 32 -2.801 29.307 13.354 1.00 0.00 H

ATOM 518 HH TYR A 32 -3.794 28.303 15.664 1.00 0.00 H

ATOM 519 N ASN A 33 3.730 26.195 10.567 1.00 0.00 N

ATOM 520 CA ASN A 33 4.940 26.326 9.765 1.00 0.00 C

ATOM 521 C ASN A 33 5.982 27.182 10.475 1.00 0.00 C

ATOM 522 O ASN A 33 6.680 27.976 9.845 1.00 0.00 O

ATOM 523 CB ASN A 33 4.616 26.902 8.399 1.00 0.00 C

ATOM 524 CG ASN A 33 5.702 26.647 7.392 1.00 0.00 C

ATOM 525 OD1 ASN A 33 6.457 25.675 7.507 1.00 0.00 O

ATOM 526 ND2 ASN A 33 5.797 27.502 6.405 1.00 0.00 N

ATOM 527 H ASN A 33 3.457 25.282 10.901 1.00 0.00 H

ATOM 528 HA ASN A 33 5.375 25.334 9.630 1.00 0.00 H

ATOM 529 1HB ASN A 33 3.686 26.467 8.032 1.00 0.00 H

ATOM 530 2HB ASN A 33 4.462 27.978 8.485 1.00 0.00 H

ATOM 531 1HD2 ASN A 33 6.501 27.383 5.705 1.00 0.00 H

ATOM 532 2HD2 ASN A 33 5.165 28.275 6.352 1.00 0.00 H

ATOM 533 N GLY A 34 6.081 27.015 11.789 1.00 0.00 N

ATOM 534 CA GLY A 34 7.070 27.738 12.580 1.00 0.00 C

ATOM 535 C GLY A 34 6.763 29.229 12.617 1.00 0.00 C

ATOM 536 O GLY A 34 7.652 30.060 12.431 1.00 0.00 O

ATOM 537 H GLY A 34 5.456 26.371 12.252 1.00 0.00 H

ATOM 538 1HA GLY A 34 7.085 27.340 13.595 1.00 0.00 H

ATOM 539 2HA GLY A 34 8.061 27.577 12.158 1.00 0.00 H

ATOM 540 N ASN A 35 5.499 29.562 12.857 1.00 0.00 N

ATOM 541 CA ASN A 35 5.072 30.955 12.913 1.00 0.00 C

ATOM 542 C ASN A 35 5.249 31.640 11.564 1.00 0.00 C

ATOM 543 O ASN A 35 5.615 32.814 11.497 1.00 0.00 O

ATOM 544 CB ASN A 35 5.828 31.703 13.996 1.00 0.00 C

ATOM 545 CG ASN A 35 5.588 31.134 15.366 1.00 0.00 C

ATOM 546 OD1 ASN A 35 4.445 30.843 15.740 1.00 0.00 O

ATOM 547 ND2 ASN A 35 6.642 30.970 16.124 1.00 0.00 N

ATOM 548 H ASN A 35 4.818 28.832 13.005 1.00 0.00 H

ATOM 549 HA ASN A 35 4.007 30.981 13.151 1.00 0.00 H

ATOM 550 1HB ASN A 35 6.897 31.668 13.783 1.00 0.00 H

ATOM 551 2HB ASN A 35 5.527 32.750 13.992 1.00 0.00 H

ATOM 552 1HD2 ASN A 35 6.542 30.595 17.046 1.00 0.00 H

ATOM 553 2HD2 ASN A 35 7.547 31.219 15.781 1.00 0.00 H

ATOM 554 N LYS A 36 4.988 30.901 10.492 1.00 0.00 N

ATOM 555 CA LYS A 36 5.060 31.453 9.144 1.00 0.00 C

ATOM 556 C LYS A 36 3.682 31.511 8.498 1.00 0.00 C

ATOM 557 O LYS A 36 3.339 32.488 7.832 1.00 0.00 O

ATOM 558 CB LYS A 36 6.013 30.628 8.277 1.00 0.00 C

ATOM 559 CG LYS A 36 7.477 30.723 8.686 1.00 0.00 C

ATOM 560 CD LYS A 36 8.361 29.889 7.772 1.00 0.00 C

ATOM 561 CE LYS A 36 9.823 29.971 8.188 1.00 0.00 C

ATOM 562 NZ LYS A 36 10.697 29.140 7.317 1.00 0.00 N

ATOM 563 H LYS A 36 4.733 29.931 10.613 1.00 0.00 H

ATOM 564 HA LYS A 36 5.442 32.473 9.208 1.00 0.00 H

ATOM 565 1HB LYS A 36 5.723 29.578 8.315 1.00 0.00 H

ATOM 566 2HB LYS A 36 5.934 30.953 7.240 1.00 0.00 H

ATOM 567 1HG LYS A 36 7.801 31.764 8.640 1.00 0.00 H

ATOM 568 2HG LYS A 36 7.592 30.370 9.711 1.00 0.00 H

ATOM 569 1HD LYS A 36 8.040 28.847 7.807 1.00 0.00 H

ATOM 570 2HD LYS A 36 8.265 30.246 6.747 1.00 0.00 H

ATOM 571 1HE LYS A 36 10.158 31.006 8.136 1.00 0.00 H

ATOM 572 2HE LYS A 36 9.928 29.630 9.218 1.00 0.00 H

ATOM 573 1HZ LYS A 36 11.656 29.221 7.625 1.00 0.00 H

ATOM 574 2HZ LYS A 36 10.407 28.173 7.372 1.00 0.00 H

ATOM 575 3HZ LYS A 36 10.623 29.459 6.362 1.00 0.00 H

ATOM 576 N LYS A 37 2.895 30.460 8.699 1.00 0.00 N

ATOM 577 CA LYS A 37 1.495 30.462 8.293 1.00 0.00 C

ATOM 578 C LYS A 37 0.632 31.222 9.292 1.00 0.00 C

ATOM 579 O LYS A 37 -0.384 31.813 8.926 1.00 0.00 O

ATOM 580 CB LYS A 37 0.980 29.030 8.137 1.00 0.00 C

ATOM 581 CG LYS A 37 1.554 28.282 6.941 1.00 0.00 C

ATOM 582 CD LYS A 37 1.005 26.866 6.859 1.00 0.00 C

ATOM 583 CE LYS A 37 1.574 26.119 5.662 1.00 0.00 C

ATOM 584 NZ LYS A 37 1.040 24.733 5.566 1.00 0.00 N

ATOM 585 H LYS A 37 3.277 29.638 9.145 1.00 0.00 H

ATOM 586 HA LYS A 37 1.415 30.968 7.330 1.00 0.00 H

ATOM 587 1HB LYS A 37 1.217 28.458 9.034 1.00 0.00 H

ATOM 588 2HB LYS A 37 -0.105 29.043 8.034 1.00 0.00 H

ATOM 589 1HG LYS A 37 1.301 28.814 6.023 1.00 0.00 H

ATOM 590 2HG LYS A 37 2.639 28.238 7.026 1.00 0.00 H

ATOM 591 1HD LYS A 37 1.261 26.323 7.770 1.00 0.00 H

ATOM 592 2HD LYS A 37 -0.081 26.901 6.771 1.00 0.00 H

ATOM 593 1HE LYS A 37 1.324 26.655 4.747 1.00 0.00 H

ATOM 594 2HE LYS A 37 2.659 26.071 5.744 1.00 0.00 H

ATOM 595 1HZ LYS A 37 1.441 24.272 4.761 1.00 0.00 H

ATOM 596 2HZ LYS A 37 1.282 24.220 6.402 1.00 0.00 H

ATOM 597 3HZ LYS A 37 0.035 24.764 5.469 1.00 0.00 H

ATOM 598 N GLY A 38 1.044 31.205 10.555 1.00 0.00 N

ATOM 599 CA GLY A 38 0.396 32.011 11.583 1.00 0.00 C

ATOM 600 C GLY A 38 1.131 31.901 12.913 1.00 0.00 C

ATOM 601 O GLY A 38 1.741 30.875 13.213 1.00 0.00 O

ATOM 602 H GLY A 38 1.825 30.618 10.810 1.00 0.00 H

ATOM 603 1HA GLY A 38 0.367 33.053 11.264 1.00 0.00 H

ATOM 604 2HA GLY A 38 -0.635 31.683 11.706 1.00 0.00 H

ATOM 605 N ASN A 39 1.069 32.965 13.707 1.00 0.00 N

ATOM 606 CA ASN A 39 1.749 32.999 14.996 1.00 0.00 C

ATOM 607 C ASN A 39 1.053 32.101 16.010 1.00 0.00 C

ATOM 608 O ASN A 39 -0.157 32.199 16.213 1.00 0.00 O

ATOM 609 CB ASN A 39 1.838 34.423 15.515 1.00 0.00 C

ATOM 610 CG ASN A 39 2.737 35.289 14.677 1.00 0.00 C

ATOM 611 OD1 ASN A 39 3.730 34.812 14.115 1.00 0.00 O

ATOM 612 ND2 ASN A 39 2.409 36.552 14.582 1.00 0.00 N

ATOM 613 H ASN A 39 0.537 33.770 13.410 1.00 0.00 H

ATOM 614 HA ASN A 39 2.762 32.614 14.864 1.00 0.00 H

ATOM 615 1HB ASN A 39 0.841 34.866 15.534 1.00 0.00 H

ATOM 616 2HB ASN A 39 2.212 34.414 16.539 1.00 0.00 H

ATOM 617 1HD2 ASN A 39 2.971 37.175 14.037 1.00 0.00 H

ATOM 618 2HD2 ASN A 39 1.597 36.894 15.053 1.00 0.00 H

ATOM 619 N ILE A 40 1.825 31.226 16.646 1.00 0.00 N

ATOM 620 CA ILE A 40 1.276 30.278 17.608 1.00 0.00 C

ATOM 621 C ILE A 40 1.108 30.919 18.979 1.00 0.00 C

ATOM 622 O ILE A 40 2.087 31.175 19.680 1.00 0.00 O

ATOM 623 CB ILE A 40 2.177 29.035 17.729 1.00 0.00 C

ATOM 624 CG1 ILE A 40 2.302 28.331 16.375 1.00 0.00 C

ATOM 625 CG2 ILE A 40 1.630 28.081 18.780 1.00 0.00 C

ATOM 626 CD1 ILE A 40 3.361 27.254 16.342 1.00 0.00 C

ATOM 627 H ILE A 40 2.817 31.217 16.457 1.00 0.00 H

ATOM 628 HA ILE A 40 0.295 29.959 17.258 1.00 0.00 H

ATOM 629 HB ILE A 40 3.182 29.341 18.018 1.00 0.00 H

ATOM 630 1HG1 ILE A 40 1.346 27.879 16.111 1.00 0.00 H

ATOM 631 2HG1 ILE A 40 2.538 29.065 15.604 1.00 0.00 H

ATOM 632 1HG2 ILE A 40 2.278 27.208 18.852 1.00 0.00 H

ATOM 633 2HG2 ILE A 40 1.592 28.586 19.744 1.00 0.00 H

ATOM 634 3HG2 ILE A 40 0.626 27.765 18.496 1.00 0.00 H

ATOM 635 1HD1 ILE A 40 3.390 26.802 15.350 1.00 0.00 H

ATOM 636 2HD1 ILE A 40 4.334 27.692 16.569 1.00 0.00 H

ATOM 637 3HD1 ILE A 40 3.126 26.490 17.081 1.00 0.00 H

ATOM 638 N THR A 41 -0.139 31.177 19.357 1.00 0.00 N

ATOM 639 CA THR A 41 -0.449 31.665 20.695 1.00 0.00 C

ATOM 640 C THR A 41 -0.822 30.519 21.627 1.00 0.00 C

ATOM 641 O THR A 41 -0.886 29.362 21.209 1.00 0.00 O

ATOM 642 CB THR A 41 -1.594 32.695 20.659 1.00 0.00 C

ATOM 643 OG1 THR A 41 -2.810 32.048 20.263 1.00 0.00 O

ATOM 644 CG2 THR A 41 -1.275 33.812 19.678 1.00 0.00 C

ATOM 645 H THR A 41 -0.892 31.032 18.699 1.00 0.00 H

ATOM 646 HA THR A 41 0.438 32.152 21.100 1.00 0.00 H

ATOM 647 HB THR A 41 -1.732 33.120 21.653 1.00 0.00 H

ATOM 648 HG1 THR A 41 -2.742 31.775 19.344 1.00 0.00 H

ATOM 649 1HG2 THR A 41 -2.094 34.530 19.666 1.00 0.00 H

ATOM 650 2HG2 THR A 41 -0.357 34.314 19.983 1.00 0.00 H

ATOM 651 3HG2 THR A 41 -1.145 33.394 18.680 1.00 0.00 H

ATOM 652 N GLN A 42 -1.066 30.846 22.891 1.00 0.00 N

ATOM 653 CA GLN A 42 -1.529 29.861 23.861 1.00 0.00 C

ATOM 654 C GLN A 42 -2.870 29.269 23.448 1.00 0.00 C

ATOM 655 O GLN A 42 -3.132 28.086 23.669 1.00 0.00 O

ATOM 656 CB GLN A 42 -1.642 30.491 25.252 1.00 0.00 C

ATOM 657 CG GLN A 42 -2.063 29.520 26.343 1.00 0.00 C

ATOM 658 CD GLN A 42 -1.043 28.420 26.564 1.00 0.00 C

ATOM 659 OE1 GLN A 42 0.143 28.687 26.777 1.00 0.00 O

ATOM 660 NE2 GLN A 42 -1.498 27.173 26.515 1.00 0.00 N

ATOM 661 H GLN A 42 -0.928 31.801 23.187 1.00 0.00 H

ATOM 662 HA GLN A 42 -0.802 29.050 23.904 1.00 0.00 H

ATOM 663 1HB GLN A 42 -0.681 30.919 25.536 1.00 0.00 H

ATOM 664 2HB GLN A 42 -2.368 31.303 25.226 1.00 0.00 H

ATOM 665 1HG GLN A 42 -2.182 30.069 27.277 1.00 0.00 H

ATOM 666 2HG GLN A 42 -3.008 29.058 26.058 1.00 0.00 H

ATOM 667 1HE2 GLN A 42 -0.871 26.405 26.654 1.00 0.00 H

ATOM 668 2HE2 GLN A 42 -2.467 27.001 26.339 1.00 0.00 H

ATOM 669 N ARG A 43 -3.718 30.098 22.848 1.00 0.00 N

ATOM 670 CA ARG A 43 -5.005 29.641 22.338 1.00 0.00 C

ATOM 671 C ARG A 43 -4.825 28.677 21.172 1.00 0.00 C

ATOM 672 O ARG A 43 -5.604 27.739 21.003 1.00 0.00 O

ATOM 673 CB ARG A 43 -5.853 30.822 21.890 1.00 0.00 C

ATOM 674 CG ARG A 43 -6.366 31.705 23.017 1.00 0.00 C

ATOM 675 CD ARG A 43 -7.132 32.867 22.498 1.00 0.00 C

ATOM 676 NE ARG A 43 -7.625 33.714 23.572 1.00 0.00 N

ATOM 677 CZ ARG A 43 -8.231 34.904 23.391 1.00 0.00 C

ATOM 678 NH1 ARG A 43 -8.411 35.372 22.176 1.00 0.00 N

ATOM 679 NH2 ARG A 43 -8.644 35.601 24.435 1.00 0.00 N

ATOM 680 H ARG A 43 -3.464 31.069 22.743 1.00 0.00 H

ATOM 681 HA ARG A 43 -5.530 29.120 23.140 1.00 0.00 H

ATOM 682 1HB ARG A 43 -5.273 31.451 21.216 1.00 0.00 H

ATOM 683 2HB ARG A 43 -6.718 30.459 21.336 1.00 0.00 H

ATOM 684 1HG ARG A 43 -7.022 31.123 23.665 1.00 0.00 H

ATOM 685 2HG ARG A 43 -5.523 32.081 23.597 1.00 0.00 H

ATOM 686 1HD ARG A 43 -6.490 33.469 21.856 1.00 0.00 H

ATOM 687 2HD ARG A 43 -7.987 32.512 21.925 1.00 0.00 H

ATOM 688 HE ARG A 43 -7.504 33.388 24.522 1.00 0.00 H

ATOM 689 1HH1 ARG A 43 -8.095 34.839 21.377 1.00 0.00 H

ATOM 690 2HH1 ARG A 43 -8.865 36.263 22.040 1.00 0.00 H

ATOM 691 1HH2 ARG A 43 -8.506 35.241 25.370 1.00 0.00 H

ATOM 692 2HH2 ARG A 43 -9.098 36.492 24.300 1.00 0.00 H

ATOM 693 N ASP A 44 -3.793 28.914 20.370 1.00 0.00 N

ATOM 694 CA ASP A 44 -3.470 28.031 19.255 1.00 0.00 C

ATOM 695 C ASP A 44 -2.897 26.709 19.748 1.00 0.00 C

ATOM 696 O ASP A 44 -3.200 25.648 19.203 1.00 0.00 O

ATOM 697 CB ASP A 44 -2.475 28.705 18.308 1.00 0.00 C

ATOM 698 CG ASP A 44 -3.083 29.871 17.541 1.00 0.00 C

ATOM 699 OD1 ASP A 44 -3.967 29.641 16.750 1.00 0.00 O

ATOM 700 OD2 ASP A 44 -2.657 30.981 17.752 1.00 0.00 O

ATOM 701 H ASP A 44 -3.217 29.727 20.536 1.00 0.00 H

ATOM 702 HA ASP A 44 -4.388 27.821 18.704 1.00 0.00 H

ATOM 703 1HB ASP A 44 -1.620 29.069 18.878 1.00 0.00 H

ATOM 704 2HB ASP A 44 -2.102 27.972 17.591 1.00 0.00 H

ATOM 705 N VAL A 45 -2.067 26.779 20.783 1.00 0.00 N

ATOM 706 CA VAL A 45 -1.553 25.581 21.436 1.00 0.00 C

ATOM 707 C VAL A 45 -2.685 24.735 22.005 1.00 0.00 C

ATOM 708 O VAL A 45 -2.684 23.511 21.875 1.00 0.00 O

ATOM 709 CB VAL A 45 -0.583 25.964 22.570 1.00 0.00 C

ATOM 710 CG1 VAL A 45 -0.246 24.746 23.416 1.00 0.00 C

ATOM 711 CG2 VAL A 45 0.678 26.581 21.985 1.00 0.00 C

ATOM 712 H VAL A 45 -1.784 27.686 21.126 1.00 0.00 H

ATOM 713 HA VAL A 45 -1.012 24.989 20.697 1.00 0.00 H

ATOM 714 HB VAL A 45 -1.072 26.685 23.225 1.00 0.00 H

ATOM 715 1HG1 VAL A 45 0.441 25.035 24.213 1.00 0.00 H

ATOM 716 2HG1 VAL A 45 -1.159 24.342 23.853 1.00 0.00 H

ATOM 717 3HG1 VAL A 45 0.225 23.987 22.791 1.00 0.00 H

ATOM 718 1HG2 VAL A 45 1.359 26.851 22.793 1.00 0.00 H

ATOM 719 2HG2 VAL A 45 1.163 25.862 21.325 1.00 0.00 H

ATOM 720 3HG2 VAL A 45 0.417 27.475 21.418 1.00 0.00 H

ATOM 721 N ASN A 46 -3.651 25.395 22.635 1.00 0.00 N

ATOM 722 CA ASN A 46 -4.808 24.709 23.197 1.00 0.00 C

ATOM 723 C ASN A 46 -5.607 23.997 22.112 1.00 0.00 C

ATOM 724 O ASN A 46 -6.047 22.862 22.296 1.00 0.00 O

ATOM 725 CB ASN A 46 -5.689 25.682 23.957 1.00 0.00 C

ATOM 726 CG ASN A 46 -5.049 26.170 25.228 1.00 0.00 C

ATOM 727 OD1 ASN A 46 -4.061 25.597 25.699 1.00 0.00 O

ATOM 728 ND2 ASN A 46 -5.594 27.218 25.791 1.00 0.00 N

ATOM 729 H ASN A 46 -3.582 26.399 22.727 1.00 0.00 H

ATOM 730 HA ASN A 46 -4.454 23.945 23.891 1.00 0.00 H

ATOM 731 1HB ASN A 46 -5.914 26.541 23.324 1.00 0.00 H

ATOM 732 2HB ASN A 46 -6.636 25.200 24.203 1.00 0.00 H

ATOM 733 1HD2 ASN A 46 -5.210 27.587 26.638 1.00 0.00 H

ATOM 734 2HD2 ASN A 46 -6.393 27.650 25.374 1.00 0.00 H

ATOM 735 N ARG A 47 -5.790 24.670 20.982 1.00 0.00 N

ATOM 736 CA ARG A 47 -6.499 24.087 19.849 1.00 0.00 C

ATOM 737 C ARG A 47 -5.767 22.864 19.312 1.00 0.00 C

ATOM 738 O ARG A 47 -6.382 21.836 19.028 1.00 0.00 O

ATOM 739 CB ARG A 47 -6.658 25.109 18.733 1.00 0.00 C

ATOM 740 CG ARG A 47 -7.662 26.215 19.017 1.00 0.00 C

ATOM 741 CD ARG A 47 -7.619 27.276 17.979 1.00 0.00 C

ATOM 742 NE ARG A 47 -8.606 28.315 18.224 1.00 0.00 N

ATOM 743 CZ ARG A 47 -8.594 29.533 17.647 1.00 0.00 C

ATOM 744 NH1 ARG A 47 -7.643 29.848 16.796 1.00 0.00 N

ATOM 745 NH2 ARG A 47 -9.539 30.410 17.936 1.00 0.00 N

ATOM 746 H ARG A 47 -5.429 25.610 20.905 1.00 0.00 H

ATOM 747 HA ARG A 47 -7.490 23.780 20.183 1.00 0.00 H

ATOM 748 1HB ARG A 47 -5.697 25.580 18.532 1.00 0.00 H

ATOM 749 2HB ARG A 47 -6.973 24.605 17.820 1.00 0.00 H

ATOM 750 1HG ARG A 47 -8.668 25.797 19.039 1.00 0.00 H

ATOM 751 2HG ARG A 47 -7.438 26.671 19.983 1.00 0.00 H

ATOM 752 1HD ARG A 47 -6.632 27.738 17.971 1.00 0.00 H

ATOM 753 2HD ARG A 47 -7.821 26.838 17.002 1.00 0.00 H

ATOM 754 HE ARG A 47 -9.354 28.110 18.873 1.00 0.00 H

ATOM 755 1HH1 ARG A 47 -6.921 29.178 16.575 1.00 0.00 H

ATOM 756 2HH1 ARG A 47 -7.634 30.761 16.364 1.00 0.00 H

ATOM 757 1HH2 ARG A 47 -10.271 30.168 18.591 1.00 0.00 H

ATOM 758 2HH2 ARG A 47 -9.531 31.322 17.504 1.00 0.00 H

ATOM 759 N VAL A 48 -4.451 22.981 19.175 1.00 0.00 N

ATOM 760 CA VAL A 48 -3.628 21.873 18.704 1.00 0.00 C

ATOM 761 C VAL A 48 -3.658 20.709 19.686 1.00 0.00 C

ATOM 762 O VAL A 48 -3.721 19.547 19.285 1.00 0.00 O

ATOM 763 CB VAL A 48 -2.173 22.335 18.504 1.00 0.00 C

ATOM 764 CG1 VAL A 48 -1.262 21.140 18.265 1.00 0.00 C

ATOM 765 CG2 VAL A 48 -2.095 23.313 17.341 1.00 0.00 C

ATOM 766 H VAL A 48 -4.009 23.860 19.402 1.00 0.00 H

ATOM 767 HA VAL A 48 -4.022 21.532 17.746 1.00 0.00 H

ATOM 768 HB VAL A 48 -1.828 22.825 19.415 1.00 0.00 H

ATOM 769 1HG1 VAL A 48 -0.237 21.485 18.126 1.00 0.00 H

ATOM 770 2HG1 VAL A 48 -1.306 20.472 19.125 1.00 0.00 H

ATOM 771 3HG1 VAL A 48 -1.589 20.607 17.373 1.00 0.00 H

ATOM 772 1HG2 VAL A 48 -1.064 23.637 17.206 1.00 0.00 H

ATOM 773 2HG2 VAL A 48 -2.445 22.825 16.432 1.00 0.00 H

ATOM 774 3HG2 VAL A 48 -2.722 24.180 17.553 1.00 0.00 H

ATOM 775 N LYS A 49 -3.611 21.028 20.975 1.00 0.00 N

ATOM 776 CA LYS A 49 -3.745 20.019 22.019 1.00 0.00 C

ATOM 777 C LYS A 49 -5.048 19.244 21.872 1.00 0.00 C

ATOM 778 O LYS A 49 -5.068 18.018 21.986 1.00 0.00 O

ATOM 779 CB LYS A 49 -3.672 20.667 23.403 1.00 0.00 C

ATOM 780 CG LYS A 49 -3.770 19.686 24.563 1.00 0.00 C

ATOM 781 CD LYS A 49 -3.639 20.397 25.902 1.00 0.00 C

ATOM 782 CE LYS A 49 -3.779 19.424 27.063 1.00 0.00 C

ATOM 783 NZ LYS A 49 -3.653 20.107 28.379 1.00 0.00 N

ATOM 784 H LYS A 49 -3.480 21.994 21.239 1.00 0.00 H

ATOM 785 HA LYS A 49 -2.922 19.310 21.923 1.00 0.00 H

ATOM 786 1HB LYS A 49 -2.731 21.209 23.502 1.00 0.00 H

ATOM 787 2HB LYS A 49 -4.479 21.392 23.508 1.00 0.00 H

ATOM 788 1HG LYS A 49 -4.733 19.174 24.527 1.00 0.00 H

ATOM 789 2HG LYS A 49 -2.980 18.941 24.478 1.00 0.00 H

ATOM 790 1HD LYS A 49 -2.664 20.884 25.961 1.00 0.00 H

ATOM 791 2HD LYS A 49 -4.412 21.161 25.985 1.00 0.00 H

ATOM 792 1HE LYS A 49 -4.750 18.935 27.013 1.00 0.00 H

ATOM 793 2HE LYS A 49 -3.007 18.658 26.990 1.00 0.00 H

ATOM 794 1HZ LYS A 49 -3.750 19.428 29.121 1.00 0.00 H

ATOM 795 2HZ LYS A 49 -2.746 20.549 28.445 1.00 0.00 H

ATOM 796 3HZ LYS A 49 -4.375 20.807 28.466 1.00 0.00 H

ATOM 797 N ARG A 50 -6.135 19.965 21.620 1.00 0.00 N

ATOM 798 CA ARG A 50 -7.439 19.343 21.427 1.00 0.00 C

ATOM 799 C ARG A 50 -7.432 18.410 20.223 1.00 0.00 C

ATOM 800 O ARG A 50 -8.009 17.323 20.265 1.00 0.00 O

ATOM 801 CB ARG A 50 -8.515 20.403 21.237 1.00 0.00 C

ATOM 802 CG ARG A 50 -8.873 21.182 22.493 1.00 0.00 C

ATOM 803 CD ARG A 50 -9.866 22.251 22.211 1.00 0.00 C

ATOM 804 NE ARG A 50 -10.218 22.993 23.411 1.00 0.00 N

ATOM 805 CZ ARG A 50 -11.040 24.060 23.436 1.00 0.00 C

ATOM 806 NH1 ARG A 50 -11.586 24.496 22.322 1.00 0.00 N

ATOM 807 NH2 ARG A 50 -11.299 24.668 24.581 1.00 0.00 N

ATOM 808 H ARG A 50 -6.055 20.970 21.559 1.00 0.00 H

ATOM 809 HA ARG A 50 -7.679 18.760 22.316 1.00 0.00 H

ATOM 810 1HB ARG A 50 -8.189 21.121 20.486 1.00 0.00 H

ATOM 811 2HB ARG A 50 -9.427 19.934 20.868 1.00 0.00 H

ATOM 812 1HG ARG A 50 -9.300 20.504 23.232 1.00 0.00 H

ATOM 813 2HG ARG A 50 -7.975 21.647 22.902 1.00 0.00 H

ATOM 814 1HD ARG A 50 -9.451 22.951 21.487 1.00 0.00 H

ATOM 815 2HD ARG A 50 -10.774 21.806 21.807 1.00 0.00 H

ATOM 816 HE ARG A 50 -9.818 22.687 24.288 1.00 0.00 H

ATOM 817 1HH1 ARG A 50 -11.388 24.031 21.447 1.00 0.00 H

ATOM 818 2HH1 ARG A 50 -12.202 25.296 22.341 1.00 0.00 H

ATOM 819 1HH2 ARG A 50 -10.879 24.333 25.437 1.00 0.00 H

ATOM 820 2HH2 ARG A 50 -11.915 25.467 24.600 1.00 0.00 H

ATOM 821 N MET A 51 -6.776 18.841 19.151 1.00 0.00 N

ATOM 822 CA MET A 51 -6.630 18.014 17.959 1.00 0.00 C

ATOM 823 C MET A 51 -5.981 16.677 18.292 1.00 0.00 C

ATOM 824 O MET A 51 -6.368 15.638 17.757 1.00 0.00 O

ATOM 825 CB MET A 51 -5.815 18.754 16.900 1.00 0.00 C

ATOM 826 CG MET A 51 -6.531 19.939 16.268 1.00 0.00 C

ATOM 827 SD MET A 51 -5.461 20.894 15.174 1.00 0.00 S

ATOM 828 CE MET A 51 -5.197 19.720 13.849 1.00 0.00 C

ATOM 829 H MET A 51 -6.368 19.764 19.162 1.00 0.00 H

ATOM 830 HA MET A 51 -7.622 17.808 17.557 1.00 0.00 H

ATOM 831 1HB MET A 51 -4.890 19.120 17.344 1.00 0.00 H

ATOM 832 2HB MET A 51 -5.542 18.062 16.102 1.00 0.00 H

ATOM 833 1HG MET A 51 -7.384 19.583 15.691 1.00 0.00 H

ATOM 834 2HG MET A 51 -6.901 20.600 17.051 1.00 0.00 H

ATOM 835 1HE MET A 51 -4.552 20.165 13.091 1.00 0.00 H

ATOM 836 2HE MET A 51 -4.723 18.821 14.247 1.00 0.00 H

ATOM 837 3HE MET A 51 -6.155 19.456 13.400 1.00 0.00 H

ATOM 838 N ILE A 52 -4.992 16.710 19.178 1.00 0.00 N

ATOM 839 CA ILE A 52 -4.372 15.489 19.680 1.00 0.00 C

ATOM 840 C ILE A 52 -5.359 14.666 20.498 1.00 0.00 C

ATOM 841 O ILE A 52 -5.495 13.460 20.293 1.00 0.00 O

ATOM 842 CB ILE A 52 -3.137 15.814 20.541 1.00 0.00 C

ATOM 843 CG1 ILE A 52 -2.023 16.406 19.673 1.00 0.00 C

ATOM 844 CG2 ILE A 52 -2.649 14.567 21.262 1.00 0.00 C

ATOM 845 CD1 ILE A 52 -0.882 17.000 20.467 1.00 0.00 C

ATOM 846 H ILE A 52 -4.661 17.603 19.513 1.00 0.00 H

ATOM 847 HA ILE A 52 -4.051 14.889 18.830 1.00 0.00 H

ATOM 848 HB ILE A 52 -3.398 16.571 21.279 1.00 0.00 H

ATOM 849 1HG1 ILE A 52 -1.619 15.632 19.022 1.00 0.00 H

ATOM 850 2HG1 ILE A 52 -2.435 17.187 19.034 1.00 0.00 H

ATOM 851 1HG2 ILE A 52 -1.775 14.815 21.865 1.00 0.00 H

ATOM 852 2HG2 ILE A 52 -3.440 14.188 21.908 1.00 0.00 H

ATOM 853 3HG2 ILE A 52 -2.381 13.805 20.531 1.00 0.00 H

ATOM 854 1HD1 ILE A 52 -0.133 17.400 19.784 1.00 0.00 H

ATOM 855 2HD1 ILE A 52 -1.259 17.803 21.101 1.00 0.00 H

ATOM 856 3HD1 ILE A 52 -0.430 16.229 21.089 1.00 0.00 H

ATOM 857 N ARG A 53 -6.046 15.325 21.425 1.00 0.00 N

ATOM 858 CA ARG A 53 -6.917 14.635 22.369 1.00 0.00 C

ATOM 859 C ARG A 53 -8.100 13.990 21.657 1.00 0.00 C

ATOM 860 O ARG A 53 -8.698 13.040 22.162 1.00 0.00 O

ATOM 861 CB ARG A 53 -7.430 15.599 23.427 1.00 0.00 C

ATOM 862 CG ARG A 53 -6.362 16.161 24.353 1.00 0.00 C

ATOM 863 CD ARG A 53 -5.742 15.098 25.184 1.00 0.00 C

ATOM 864 NE ARG A 53 -6.659 14.596 26.195 1.00 0.00 N

ATOM 865 CZ ARG A 53 -6.547 13.398 26.802 1.00 0.00 C

ATOM 866 NH1 ARG A 53 -5.556 12.593 26.489 1.00 0.00 N

ATOM 867 NH2 ARG A 53 -7.435 13.033 27.711 1.00 0.00 N

ATOM 868 H ARG A 53 -5.963 16.330 21.477 1.00 0.00 H

ATOM 869 HA ARG A 53 -6.341 13.852 22.862 1.00 0.00 H

ATOM 870 1HB ARG A 53 -7.923 16.441 22.944 1.00 0.00 H

ATOM 871 2HB ARG A 53 -8.173 15.097 24.048 1.00 0.00 H

ATOM 872 1HG ARG A 53 -5.579 16.633 23.759 1.00 0.00 H

ATOM 873 2HG ARG A 53 -6.809 16.900 25.018 1.00 0.00 H

ATOM 874 1HD ARG A 53 -5.446 14.264 24.548 1.00 0.00 H

ATOM 875 2HD ARG A 53 -4.863 15.498 25.689 1.00 0.00 H

ATOM 876 HE ARG A 53 -7.434 15.188 26.462 1.00 0.00 H

ATOM 877 1HH1 ARG A 53 -4.878 12.872 25.795 1.00 0.00 H

ATOM 878 2HH1 ARG A 53 -5.473 11.695 26.944 1.00 0.00 H

ATOM 879 1HH2 ARG A 53 -8.197 13.652 27.951 1.00 0.00 H

ATOM 880 2HH2 ARG A 53 -7.351 12.136 28.165 1.00 0.00 H

ATOM 881 N ASP A 54 -8.433 14.513 20.482 1.00 0.00 N

ATOM 882 CA ASP A 54 -9.529 13.973 19.686 1.00 0.00 C

ATOM 883 C ASP A 54 -9.213 12.566 19.196 1.00 0.00 C

ATOM 884 O ASP A 54 -10.116 11.789 18.884 1.00 0.00 O

ATOM 885 CB ASP A 54 -9.823 14.883 18.491 1.00 0.00 C

ATOM 886 CG ASP A 54 -10.533 16.171 18.887 1.00 0.00 C

ATOM 887 OD1 ASP A 54 -11.052 16.229 19.976 1.00 0.00 O

ATOM 888 OD2 ASP A 54 -10.548 17.084 18.096 1.00 0.00 O

ATOM 889 H ASP A 54 -7.913 15.304 20.130 1.00 0.00 H

ATOM 890 HA ASP A 54 -10.420 13.923 20.313 1.00 0.00 H

ATOM 891 1HB ASP A 54 -8.890 15.139 17.990 1.00 0.00 H

ATOM 892 2HB ASP A 54 -10.445 14.349 17.772 1.00 0.00 H

ATOM 893 N VAL A 55 -7.926 12.243 19.129 1.00 0.00 N

ATOM 894 CA VAL A 55 -7.484 10.963 18.589 1.00 0.00 C

ATOM 895 C VAL A 55 -6.549 10.250 19.557 1.00 0.00 C

ATOM 896 O VAL A 55 -6.124 9.122 19.308 1.00 0.00 O

ATOM 897 CB VAL A 55 -6.763 11.171 17.244 1.00 0.00 C

ATOM 898 CG1 VAL A 55 -7.712 11.773 16.218 1.00 0.00 C

ATOM 899 CG2 VAL A 55 -5.546 12.062 17.440 1.00 0.00 C

ATOM 900 H VAL A 55 -7.236 12.902 19.461 1.00 0.00 H

ATOM 901 HA VAL A 55 -8.360 10.336 18.423 1.00 0.00 H

ATOM 902 HB VAL A 55 -6.446 10.202 16.858 1.00 0.00 H

ATOM 903 1HG1 VAL A 55 -7.186 11.913 15.273 1.00 0.00 H

ATOM 904 2HG1 VAL A 55 -8.557 11.101 16.067 1.00 0.00 H

ATOM 905 3HG1 VAL A 55 -8.073 12.736 16.578 1.00 0.00 H

ATOM 906 1HG2 VAL A 55 -5.041 12.204 16.485 1.00 0.00 H

ATOM 907 2HG2 VAL A 55 -5.863 13.029 17.831 1.00 0.00 H

ATOM 908 3HG2 VAL A 55 -4.861 11.592 18.146 1.00 0.00 H

ATOM 909 N ASP A 56 -6.232 10.915 20.663 1.00 0.00 N

ATOM 910 CA ASP A 56 -5.333 10.352 21.664 1.00 0.00 C

ATOM 911 C ASP A 56 -5.985 9.187 22.397 1.00 0.00 C

ATOM 912 O ASP A 56 -6.453 9.335 23.526 1.00 0.00 O

ATOM 913 CB ASP A 56 -4.913 11.425 22.672 1.00 0.00 C

ATOM 914 CG ASP A 56 -3.836 10.944 23.635 1.00 0.00 C

ATOM 915 OD1 ASP A 56 -3.290 9.891 23.407 1.00 0.00 O

ATOM 916 OD2 ASP A 56 -3.572 11.633 24.591 1.00 0.00 O

ATOM 917 H ASP A 56 -6.623 11.833 20.815 1.00 0.00 H

ATOM 918 HA ASP A 56 -4.441 9.980 21.159 1.00 0.00 H

ATOM 919 1HB ASP A 56 -4.537 12.298 22.138 1.00 0.00 H

ATOM 920 2HB ASP A 56 -5.781 11.742 23.249 1.00 0.00 H

ATOM 921 N LYS A 57 -6.013 8.027 21.748 1.00 0.00 N

ATOM 922 CA LYS A 57 -6.732 6.873 22.274 1.00 0.00 C

ATOM 923 C LYS A 57 -6.050 6.318 23.517 1.00 0.00 C

ATOM 924 O LYS A 57 -6.712 5.930 24.480 1.00 0.00 O

ATOM 925 CB LYS A 57 -6.848 5.783 21.206 1.00 0.00 C

ATOM 926 CG LYS A 57 -7.762 6.139 20.041 1.00 0.00 C

ATOM 927 CD LYS A 57 -7.839 5.002 19.032 1.00 0.00 C

ATOM 928 CE LYS A 57 -8.645 5.403 17.806 1.00 0.00 C

ATOM 929 NZ LYS A 57 -8.769 4.286 16.831 1.00 0.00 N

ATOM 930 H LYS A 57 -5.524 7.944 20.869 1.00 0.00 H

ATOM 931 HA LYS A 57 -7.736 7.190 22.558 1.00 0.00 H

ATOM 932 1HB LYS A 57 -5.860 5.564 20.801 1.00 0.00 H

ATOM 933 2HB LYS A 57 -7.227 4.867 21.661 1.00 0.00 H

ATOM 934 1HG LYS A 57 -8.765 6.349 20.416 1.00 0.00 H

ATOM 935 2HG LYS A 57 -7.385 7.031 19.542 1.00 0.00 H

ATOM 936 1HD LYS A 57 -6.832 4.724 18.720 1.00 0.00 H

ATOM 937 2HD LYS A 57 -8.309 4.135 19.496 1.00 0.00 H

ATOM 938 1HE LYS A 57 -9.643 5.714 18.113 1.00 0.00 H

ATOM 939 2HE LYS A 57 -8.162 6.247 17.313 1.00 0.00 H

ATOM 940 1HZ LYS A 57 -9.309 4.592 16.034 1.00 0.00 H

ATOM 941 2HZ LYS A 57 -7.849 4.001 16.526 1.00 0.00 H

ATOM 942 3HZ LYS A 57 -9.233 3.504 17.272 1.00 0.00 H

ATOM 943 N ASN A 58 -4.722 6.282 23.490 1.00 0.00 N

ATOM 944 CA ASN A 58 -3.951 5.643 24.551 1.00 0.00 C

ATOM 945 C ASN A 58 -3.642 6.624 25.674 1.00 0.00 C

ATOM 946 O ASN A 58 -3.030 6.260 26.678 1.00 0.00 O

ATOM 947 CB ASN A 58 -2.671 5.046 23.995 1.00 0.00 C

ATOM 948 CG ASN A 58 -1.679 6.095 23.576 1.00 0.00 C

ATOM 949 OD1 ASN A 58 -1.732 7.239 24.041 1.00 0.00 O

ATOM 950 ND2 ASN A 58 -0.774 5.727 22.705 1.00 0.00 N

ATOM 951 H ASN A 58 -4.233 6.708 22.716 1.00 0.00 H

ATOM 952 HA ASN A 58 -4.552 4.840 24.980 1.00 0.00 H

ATOM 953 1HB ASN A 58 -2.210 4.407 24.750 1.00 0.00 H

ATOM 954 2HB ASN A 58 -2.905 4.420 23.135 1.00 0.00 H

ATOM 955 1HD2 ASN A 58 -0.087 6.383 22.390 1.00 0.00 H

ATOM 956 2HD2 ASN A 58 -0.768 4.791 22.355 1.00 0.00 H

ATOM 957 N ASN A 59 -4.068 7.871 25.498 1.00 0.00 N

ATOM 958 CA ASN A 59 -4.061 8.843 26.585 1.00 0.00 C

ATOM 959 C ASN A 59 -2.648 9.080 27.103 1.00 0.00 C

ATOM 960 O ASN A 59 -2.353 8.822 28.270 1.00 0.00 O

ATOM 961 CB ASN A 59 -4.974 8.392 27.710 1.00 0.00 C

ATOM 962 CG ASN A 59 -5.261 9.491 28.696 1.00 0.00 C

ATOM 963 OD1 ASN A 59 -5.392 10.661 28.319 1.00 0.00 O

ATOM 964 ND2 ASN A 59 -5.362 9.137 29.952 1.00 0.00 N

ATOM 965 H ASN A 59 -4.406 8.151 24.589 1.00 0.00 H

ATOM 966 HA ASN A 59 -4.428 9.795 26.199 1.00 0.00 H

ATOM 967 1HB ASN A 59 -5.918 8.038 27.294 1.00 0.00 H

ATOM 968 2HB ASN A 59 -4.515 7.557 28.239 1.00 0.00 H

ATOM 969 1HD2 ASN A 59 -5.552 9.826 30.652 1.00 0.00 H

ATOM 970 2HD2 ASN A 59 -5.248 8.179 30.212 1.00 0.00 H

ATOM 971 N ASP A 60 -1.778 9.573 26.228 1.00 0.00 N

ATOM 972 CA ASP A 60 -0.427 9.954 26.624 1.00 0.00 C

ATOM 973 C ASP A 60 -0.093 11.365 26.154 1.00 0.00 C

ATOM 974 O ASP A 60 1.038 11.827 26.302 1.00 0.00 O

ATOM 975 CB ASP A 60 0.596 8.966 26.058 1.00 0.00 C

ATOM 976 CG ASP A 60 0.695 9.020 24.540 1.00 0.00 C

ATOM 977 OD1 ASP A 60 0.060 9.862 23.952 1.00 0.00 O

ATOM 978 OD2 ASP A 60 1.405 8.217 23.983 1.00 0.00 O

ATOM 979 H ASP A 60 -2.059 9.687 25.265 1.00 0.00 H

ATOM 980 HA ASP A 60 -0.367 9.934 27.712 1.00 0.00 H

ATOM 981 1HB ASP A 60 1.579 9.180 26.480 1.00 0.00 H

ATOM 982 2HB ASP A 60 0.326 7.953 26.355 1.00 0.00 H

ATOM 983 N GLY A 61 -1.084 12.044 25.587 1.00 0.00 N

ATOM 984 CA GLY A 61 -0.948 13.455 25.244 1.00 0.00 C

ATOM 985 C GLY A 61 -0.205 13.632 23.927 1.00 0.00 C

ATOM 986 O GLY A 61 0.171 14.746 23.560 1.00 0.00 O

ATOM 987 H GLY A 61 -1.955 11.571 25.388 1.00 0.00 H

ATOM 988 1HA GLY A 61 -1.937 13.909 25.173 1.00 0.00 H

ATOM 989 2HA GLY A 61 -0.414 13.972 26.040 1.00 0.00 H

ATOM 990 N LYS A 62 0.004 12.528 23.218 1.00 0.00 N

ATOM 991 CA LYS A 62 0.713 12.558 21.944 1.00 0.00 C

ATOM 992 C LYS A 62 -0.063 11.815 20.864 1.00 0.00 C

ATOM 993 O LYS A 62 -1.086 11.188 21.142 1.00 0.00 O

ATOM 994 CB LYS A 62 2.111 11.957 22.094 1.00 0.00 C

ATOM 995 CG LYS A 62 3.011 12.696 23.076 1.00 0.00 C

ATOM 996 CD LYS A 62 4.403 12.083 23.119 1.00 0.00 C

ATOM 997 CE LYS A 62 5.270 12.749 24.177 1.00 0.00 C

ATOM 998 NZ LYS A 62 6.643 12.177 24.213 1.00 0.00 N

ATOM 999 H LYS A 62 -0.336 11.645 23.571 1.00 0.00 H

ATOM 1000 HA LYS A 62 0.812 13.597 21.628 1.00 0.00 H

ATOM 1001 1HB LYS A 62 2.029 10.923 22.430 1.00 0.00 H

ATOM 1002 2HB LYS A 62 2.610 11.948 21.125 1.00 0.00 H

ATOM 1003 1HG LYS A 62 3.093 13.742 22.778 1.00 0.00 H

ATOM 1004 2HG LYS A 62 2.574 12.654 24.073 1.00 0.00 H

ATOM 1005 1HD LYS A 62 4.325 11.018 23.343 1.00 0.00 H

ATOM 1006 2HD LYS A 62 4.881 12.197 22.146 1.00 0.00 H

ATOM 1007 1HE LYS A 62 5.340 13.816 23.971 1.00 0.00 H

ATOM 1008 2HE LYS A 62 4.811 12.620 25.157 1.00 0.00 H

ATOM 1009 1HZ LYS A 62 7.185 12.644 24.926 1.00 0.00 H

ATOM 1010 2HZ LYS A 62 6.592 11.189 24.422 1.00 0.00 H

ATOM 1011 3HZ LYS A 62 7.086 12.307 23.315 1.00 0.00 H

ATOM 1012 N ILE A 63 0.428 11.889 19.632 1.00 0.00 N

ATOM 1013 CA ILE A 63 -0.146 11.125 18.531 1.00 0.00 C

ATOM 1014 C ILE A 63 0.837 10.083 18.012 1.00 0.00 C

ATOM 1015 O ILE A 63 1.933 10.418 17.563 1.00 0.00 O

ATOM 1016 CB ILE A 63 -0.565 12.055 17.378 1.00 0.00 C

ATOM 1017 CG1 ILE A 63 -1.640 13.038 17.848 1.00 0.00 C

ATOM 1018 CG2 ILE A 63 -1.064 11.242 16.193 1.00 0.00 C

ATOM 1019 CD1 ILE A 63 -1.999 14.088 16.822 1.00 0.00 C

ATOM 1020 H ILE A 63 1.220 12.490 19.454 1.00 0.00 H

ATOM 1021 HA ILE A 63 -1.032 10.607 18.895 1.00 0.00 H

ATOM 1022 HB ILE A 63 0.290 12.651 17.062 1.00 0.00 H

ATOM 1023 1HG1 ILE A 63 -2.546 12.490 18.106 1.00 0.00 H

ATOM 1024 2HG1 ILE A 63 -1.299 13.546 18.750 1.00 0.00 H

ATOM 1025 1HG2 ILE A 63 -1.357 11.914 15.388 1.00 0.00 H

ATOM 1026 2HG2 ILE A 63 -0.271 10.582 15.845 1.00 0.00 H

ATOM 1027 3HG2 ILE A 63 -1.924 10.645 16.498 1.00 0.00 H

ATOM 1028 1HD1 ILE A 63 -2.766 14.747 17.228 1.00 0.00 H

ATOM 1029 2HD1 ILE A 63 -1.112 14.673 16.575 1.00 0.00 H

ATOM 1030 3HD1 ILE A 63 -2.377 13.604 15.923 1.00 0.00 H

ATOM 1031 N ASP A 64 0.438 8.817 18.077 1.00 0.00 N

ATOM 1032 CA ASP A 64 1.210 7.738 17.473 1.00 0.00 C

ATOM 1033 C ASP A 64 0.736 7.450 16.054 1.00 0.00 C

ATOM 1034 O ASP A 64 -0.149 8.130 15.535 1.00 0.00 O

ATOM 1035 CB ASP A 64 1.110 6.467 18.319 1.00 0.00 C

ATOM 1036 CG ASP A 64 -0.277 5.839 18.283 1.00 0.00 C

ATOM 1037 OD1 ASP A 64 -0.996 6.086 17.344 1.00 0.00 O

ATOM 1038 OD2 ASP A 64 -0.605 5.118 19.196 1.00 0.00 O

ATOM 1039 H ASP A 64 -0.423 8.597 18.558 1.00 0.00 H

ATOM 1040 HA ASP A 64 2.255 8.045 17.427 1.00 0.00 H

ATOM 1041 1HB ASP A 64 1.834 5.734 17.963 1.00 0.00 H

ATOM 1042 2HB ASP A 64 1.361 6.699 19.355 1.00 0.00 H

ATOM 1043 N PHE A 65 1.330 6.437 15.432 1.00 0.00 N

ATOM 1044 CA PHE A 65 1.109 6.173 14.015 1.00 0.00 C

ATOM 1045 C PHE A 65 -0.346 5.814 13.743 1.00 0.00 C

ATOM 1046 O PHE A 65 -0.925 6.243 12.745 1.00 0.00 O

ATOM 1047 CB PHE A 65 2.017 5.039 13.535 1.00 0.00 C

ATOM 1048 CG PHE A 65 3.416 5.480 13.210 1.00 0.00 C

ATOM 1049 CD1 PHE A 65 4.434 5.349 14.143 1.00 0.00 C

ATOM 1050 CD2 PHE A 65 3.717 6.027 11.972 1.00 0.00 C

ATOM 1051 CE1 PHE A 65 5.722 5.755 13.845 1.00 0.00 C

ATOM 1052 CE2 PHE A 65 5.002 6.432 11.671 1.00 0.00 C

ATOM 1053 CZ PHE A 65 6.006 6.296 12.609 1.00 0.00 C

ATOM 1054 H PHE A 65 1.951 5.835 15.954 1.00 0.00 H

ATOM 1055 HA PHE A 65 1.350 7.075 13.453 1.00 0.00 H

ATOM 1056 1HB PHE A 65 2.074 4.268 14.303 1.00 0.00 H

ATOM 1057 2HB PHE A 65 1.589 4.583 12.644 1.00 0.00 H

ATOM 1058 HD1 PHE A 65 4.210 4.920 15.120 1.00 0.00 H

ATOM 1059 HD2 PHE A 65 2.924 6.135 11.231 1.00 0.00 H

ATOM 1060 HE1 PHE A 65 6.512 5.646 14.587 1.00 0.00 H

ATOM 1061 HE2 PHE A 65 5.225 6.860 10.694 1.00 0.00 H

ATOM 1062 HZ PHE A 65 7.020 6.617 12.374 1.00 0.00 H

ATOM 1063 N HIS A 66 -0.933 5.024 14.636 1.00 0.00 N

ATOM 1064 CA HIS A 66 -2.342 4.664 14.532 1.00 0.00 C

ATOM 1065 C HIS A 66 -3.237 5.885 14.701 1.00 0.00 C

ATOM 1066 O HIS A 66 -4.113 6.143 13.877 1.00 0.00 O

ATOM 1067 CB HIS A 66 -2.709 3.605 15.577 1.00 0.00 C

ATOM 1068 CG HIS A 66 -4.173 3.296 15.632 1.00 0.00 C

ATOM 1069 ND1 HIS A 66 -4.811 2.547 14.666 1.00 0.00 N

ATOM 1070 CD2 HIS A 66 -5.122 3.635 16.535 1.00 0.00 C

ATOM 1071 CE1 HIS A 66 -6.092 2.438 14.974 1.00 0.00 C

ATOM 1072 NE2 HIS A 66 -6.306 3.090 16.103 1.00 0.00 N

ATOM 1073 H HIS A 66 -0.389 4.665 15.407 1.00 0.00 H

ATOM 1074 HA HIS A 66 -2.538 4.246 13.545 1.00 0.00 H

ATOM 1075 1HB HIS A 66 -2.172 2.680 15.362 1.00 0.00 H

ATOM 1076 2HB HIS A 66 -2.394 3.944 16.563 1.00 0.00 H

ATOM 1077 HD1 HIS A 66 -4.376 2.081 13.895 1.00 0.00 H

ATOM 1078 HD2 HIS A 66 -5.093 4.216 17.458 1.00 0.00 H

ATOM 1079 HE1 HIS A 66 -6.772 1.879 14.332 1.00 0.00 H

ATOM 1080 N GLU A 67 -3.011 6.633 15.776 1.00 0.00 N

ATOM 1081 CA GLU A 67 -3.811 7.816 16.068 1.00 0.00 C

ATOM 1082 C GLU A 67 -3.641 8.876 14.988 1.00 0.00 C

ATOM 1083 O GLU A 67 -4.555 9.656 14.720 1.00 0.00 O

ATOM 1084 CB GLU A 67 -3.428 8.396 17.431 1.00 0.00 C

ATOM 1085 CG GLU A 67 -3.697 7.469 18.608 1.00 0.00 C

ATOM 1086 CD GLU A 67 -3.070 7.949 19.886 1.00 0.00 C

ATOM 1087 OE1 GLU A 67 -2.102 8.668 19.818 1.00 0.00 O

ATOM 1088 OE2 GLU A 67 -3.559 7.597 20.934 1.00 0.00 O

ATOM 1089 H GLU A 67 -2.266 6.375 16.407 1.00 0.00 H

ATOM 1090 HA GLU A 67 -4.862 7.525 16.095 1.00 0.00 H

ATOM 1091 1HB GLU A 67 -2.366 8.643 17.436 1.00 0.00 H

ATOM 1092 2HB GLU A 67 -3.980 9.321 17.601 1.00 0.00 H

ATOM 1093 1HG GLU A 67 -4.774 7.387 18.753 1.00 0.00 H

ATOM 1094 2HG GLU A 67 -3.314 6.478 18.370 1.00 0.00 H

ATOM 1095 N PHE A 68 -2.465 8.900 14.370 1.00 0.00 N

ATOM 1096 CA PHE A 68 -2.208 9.794 13.247 1.00 0.00 C

ATOM 1097 C PHE A 68 -3.110 9.466 12.064 1.00 0.00 C

ATOM 1098 O PHE A 68 -3.725 10.355 11.474 1.00 0.00 O

ATOM 1099 CB PHE A 68 -0.743 9.704 12.817 1.00 0.00 C

ATOM 1100 CG PHE A 68 -0.387 10.621 11.681 1.00 0.00 C

ATOM 1101 CD1 PHE A 68 -0.301 11.991 11.877 1.00 0.00 C

ATOM 1102 CD2 PHE A 68 -0.139 10.115 10.414 1.00 0.00 C

ATOM 1103 CE1 PHE A 68 0.026 12.835 10.833 1.00 0.00 C

ATOM 1104 CE2 PHE A 68 0.190 10.956 9.369 1.00 0.00 C

ATOM 1105 CZ PHE A 68 0.272 12.318 9.579 1.00 0.00 C

ATOM 1106 H PHE A 68 -1.730 8.284 14.686 1.00 0.00 H

ATOM 1107 HA PHE A 68 -2.417 10.817 13.563 1.00 0.00 H

ATOM 1108 1HB PHE A 68 -0.100 9.946 13.663 1.00 0.00 H

ATOM 1109 2HB PHE A 68 -0.514 8.683 12.514 1.00 0.00 H

ATOM 1110 HD1 PHE A 68 -0.494 12.400 12.870 1.00 0.00 H

ATOM 1111 HD2 PHE A 68 -0.204 9.039 10.248 1.00 0.00 H

ATOM 1112 HE1 PHE A 68 0.090 13.910 11.001 1.00 0.00 H

ATOM 1113 HE2 PHE A 68 0.383 10.546 8.378 1.00 0.00 H

ATOM 1114 HZ PHE A 68 0.528 12.983 8.755 1.00 0.00 H

ATOM 1115 N SER A 69 -3.186 8.184 11.721 1.00 0.00 N

ATOM 1116 CA SER A 69 -4.049 7.731 10.637 1.00 0.00 C

ATOM 1117 C SER A 69 -5.519 7.913 10.990 1.00 0.00 C

ATOM 1118 O SER A 69 -6.368 8.042 10.108 1.00 0.00 O

ATOM 1119 CB SER A 69 -3.772 6.273 10.325 1.00 0.00 C

ATOM 1120 OG SER A 69 -4.218 5.444 11.362 1.00 0.00 O

ATOM 1121 H SER A 69 -2.631 7.507 12.225 1.00 0.00 H

ATOM 1122 HA SER A 69 -3.833 8.328 9.750 1.00 0.00 H

ATOM 1123 1HB SER A 69 -4.271 5.999 9.396 1.00 0.00 H

ATOM 1124 2HB SER A 69 -2.702 6.130 10.175 1.00 0.00 H

ATOM 1125 HG SER A 69 -4.123 5.955 12.169 1.00 0.00 H

ATOM 1126 N GLU A 70 -5.815 7.923 12.286 1.00 0.00 N

ATOM 1127 CA GLU A 70 -7.155 8.241 12.764 1.00 0.00 C

ATOM 1128 C GLU A 70 -7.474 9.717 12.568 1.00 0.00 C

ATOM 1129 O GLU A 70 -8.577 10.074 12.153 1.00 0.00 O

ATOM 1130 CB GLU A 70 -7.296 7.869 14.241 1.00 0.00 C

ATOM 1131 CG GLU A 70 -8.671 8.151 14.832 1.00 0.00 C

ATOM 1132 CD GLU A 70 -9.753 7.302 14.226 1.00 0.00 C

ATOM 1133 OE1 GLU A 70 -9.536 6.126 14.058 1.00 0.00 O

ATOM 1134 OE2 GLU A 70 -10.799 7.831 13.929 1.00 0.00 O

ATOM 1135 H GLU A 70 -5.092 7.706 12.957 1.00 0.00 H

ATOM 1136 HA GLU A 70 -7.875 7.659 12.187 1.00 0.00 H

ATOM 1137 1HB GLU A 70 -7.088 6.807 14.371 1.00 0.00 H

ATOM 1138 2HB GLU A 70 -6.561 8.421 14.827 1.00 0.00 H

ATOM 1139 1HG GLU A 70 -8.638 7.966 15.905 1.00 0.00 H

ATOM 1140 2HG GLU A 70 -8.911 9.202 14.679 1.00 0.00 H

ATOM 1141 N MET A 71 -6.503 10.572 12.871 1.00 0.00 N

ATOM 1142 CA MET A 71 -6.640 12.003 12.628 1.00 0.00 C

ATOM 1143 C MET A 71 -6.783 12.299 11.140 1.00 0.00 C

ATOM 1144 O MET A 71 -7.613 13.114 10.737 1.00 0.00 O

ATOM 1145 CB MET A 71 -5.441 12.753 13.207 1.00 0.00 C

ATOM 1146 CG MET A 71 -5.486 14.262 13.007 1.00 0.00 C

ATOM 1147 SD MET A 71 -4.065 15.102 13.733 1.00 0.00 S

ATOM 1148 CE MET A 71 -2.755 14.556 12.643 1.00 0.00 C

ATOM 1149 H MET A 71 -5.648 10.222 13.279 1.00 0.00 H

ATOM 1150 HA MET A 71 -7.545 12.353 13.123 1.00 0.00 H

ATOM 1151 1HB MET A 71 -5.374 12.560 14.276 1.00 0.00 H

ATOM 1152 2HB MET A 71 -4.524 12.382 12.748 1.00 0.00 H

ATOM 1153 1HG MET A 71 -5.512 14.487 11.941 1.00 0.00 H

ATOM 1154 2HG MET A 71 -6.392 14.662 13.461 1.00 0.00 H

ATOM 1155 1HE MET A 71 -1.808 14.992 12.963 1.00 0.00 H

ATOM 1156 2HE MET A 71 -2.683 13.468 12.676 1.00 0.00 H

ATOM 1157 3HE MET A 71 -2.974 14.873 11.623 1.00 0.00 H

ATOM 1158 N MET A 72 -5.971 11.631 10.328 1.00 0.00 N

ATOM 1159 CA MET A 72 -6.001 11.826 8.884 1.00 0.00 C

ATOM 1160 C MET A 72 -7.142 11.043 8.246 1.00 0.00 C

ATOM 1161 O MET A 72 -7.519 11.298 7.102 1.00 0.00 O

ATOM 1162 CB MET A 72 -4.665 11.417 8.268 1.00 0.00 C

ATOM 1163 CG MET A 72 -3.487 12.289 8.682 1.00 0.00 C

ATOM 1164 SD MET A 72 -3.706 14.018 8.216 1.00 0.00 S

ATOM 1165 CE MET A 72 -3.628 13.900 6.432 1.00 0.00 C

ATOM 1166 H MET A 72 -5.315 10.971 10.722 1.00 0.00 H

ATOM 1167 HA MET A 72 -6.172 12.883 8.681 1.00 0.00 H

ATOM 1168 1HB MET A 72 -4.434 10.390 8.549 1.00 0.00 H

ATOM 1169 2HB MET A 72 -4.739 11.450 7.180 1.00 0.00 H

ATOM 1170 1HG MET A 72 -3.358 12.236 9.762 1.00 0.00 H

ATOM 1171 2HG MET A 72 -2.577 11.918 8.211 1.00 0.00 H

ATOM 1172 1HE MET A 72 -3.748 14.893 5.996 1.00 0.00 H

ATOM 1173 2HE MET A 72 -2.663 13.487 6.136 1.00 0.00 H

ATOM 1174 3HE MET A 72 -4.427 13.249 6.074 1.00 0.00 H

ATOM 1175 N LYS A 73 -7.688 10.090 8.993 1.00 0.00 N

ATOM 1176 CA LYS A 73 -8.745 9.226 8.480 1.00 0.00 C

ATOM 1177 C LYS A 73 -8.284 8.467 7.242 1.00 0.00 C

ATOM 1178 O LYS A 73 -9.002 8.392 6.245 1.00 0.00 O

ATOM 1179 CB LYS A 73 -9.997 10.045 8.158 1.00 0.00 C

ATOM 1180 CG LYS A 73 -10.561 10.822 9.340 1.00 0.00 C

ATOM 1181 CD LYS A 73 -11.146 9.887 10.388 1.00 0.00 C

ATOM 1182 CE LYS A 73 -11.737 10.664 11.556 1.00 0.00 C

ATOM 1183 NZ LYS A 73 -12.299 9.761 12.597 1.00 0.00 N

ATOM 1184 H LYS A 73 -7.363 9.959 9.940 1.00 0.00 H

ATOM 1185 HA LYS A 73 -8.996 8.493 9.247 1.00 0.00 H

ATOM 1186 1HB LYS A 73 -9.771 10.758 7.365 1.00 0.00 H

ATOM 1187 2HB LYS A 73 -10.780 9.382 7.789 1.00 0.00 H

ATOM 1188 1HG LYS A 73 -9.768 11.416 9.797 1.00 0.00 H

ATOM 1189 2HG LYS A 73 -11.341 11.498 8.992 1.00 0.00 H

ATOM 1190 1HD LYS A 73 -11.928 9.276 9.935 1.00 0.00 H

ATOM 1191 2HD LYS A 73 -10.364 9.226 10.763 1.00 0.00 H

ATOM 1192 1HE LYS A 73 -10.964 11.284 12.008 1.00 0.00 H

ATOM 1193 2HE LYS A 73 -12.530 11.317 11.194 1.00 0.00 H

ATOM 1194 1HZ LYS A 73 -12.680 10.313 13.352 1.00 0.00 H

ATOM 1195 2HZ LYS A 73 -13.030 9.193 12.192 1.00 0.00 H

ATOM 1196 3HZ LYS A 73 -11.568 9.163 12.954 1.00 0.00 H

ATOM 1197 N LEU A 74 -7.083 7.905 7.313 1.00 0.00 N

ATOM 1198 CA LEU A 74 -6.518 7.159 6.194 1.00 0.00 C

ATOM 1199 C LEU A 74 -6.188 5.728 6.596 1.00 0.00 C

ATOM 1200 O LEU A 74 -5.909 5.449 7.762 1.00 0.00 O

ATOM 1201 CB LEU A 74 -5.253 7.856 5.677 1.00 0.00 C

ATOM 1202 CG LEU A 74 -5.440 9.300 5.194 1.00 0.00 C

ATOM 1203 CD1 LEU A 74 -4.083 9.902 4.856 1.00 0.00 C

ATOM 1204 CD2 LEU A 74 -6.361 9.316 3.984 1.00 0.00 C

ATOM 1205 H LEU A 74 -6.546 7.996 8.164 1.00 0.00 H

ATOM 1206 HA LEU A 74 -7.255 7.130 5.392 1.00 0.00 H

ATOM 1207 1HB LEU A 74 -4.512 7.867 6.475 1.00 0.00 H

ATOM 1208 2HB LEU A 74 -4.853 7.277 4.845 1.00 0.00 H

ATOM 1209 HG LEU A 74 -5.880 9.897 5.993 1.00 0.00 H

ATOM 1210 1HD1 LEU A 74 -4.215 10.929 4.513 1.00 0.00 H

ATOM 1211 2HD1 LEU A 74 -3.451 9.896 5.745 1.00 0.00 H

ATOM 1212 3HD1 LEU A 74 -3.610 9.315 4.070 1.00 0.00 H

ATOM 1213 1HD2 LEU A 74 -6.494 10.343 3.641 1.00 0.00 H

ATOM 1214 2HD2 LEU A 74 -5.922 8.720 3.184 1.00 0.00 H

ATOM 1215 3HD2 LEU A 74 -7.330 8.897 4.258 1.00 0.00 H

ATOM 1216 N LYS A 75 -6.223 4.823 5.624 1.00 0.00 N

ATOM 1217 CA LYS A 75 -5.858 3.431 5.860 1.00 0.00 C

ATOM 1218 C LYS A 75 -4.363 3.288 6.113 1.00 0.00 C

ATOM 1219 O LYS A 75 -3.543 3.749 5.320 1.00 0.00 O

ATOM 1220 CB LYS A 75 -6.277 2.559 4.675 1.00 0.00 C

ATOM 1221 CG LYS A 75 -6.012 1.071 4.864 1.00 0.00 C

ATOM 1222 CD LYS A 75 -6.518 0.265 3.677 1.00 0.00 C

ATOM 1223 CE LYS A 75 -6.252 -1.222 3.863 1.00 0.00 C

ATOM 1224 NZ LYS A 75 -6.743 -2.023 2.709 1.00 0.00 N

ATOM 1225 H LYS A 75 -6.508 5.106 4.698 1.00 0.00 H

ATOM 1226 HA LYS A 75 -6.382 3.082 6.751 1.00 0.00 H

ATOM 1227 1HB LYS A 75 -7.343 2.687 4.487 1.00 0.00 H

ATOM 1228 2HB LYS A 75 -5.746 2.883 3.780 1.00 0.00 H

ATOM 1229 1HG LYS A 75 -4.940 0.904 4.974 1.00 0.00 H

ATOM 1230 2HG LYS A 75 -6.511 0.724 5.768 1.00 0.00 H

ATOM 1231 1HD LYS A 75 -7.592 0.422 3.562 1.00 0.00 H

ATOM 1232 2HD LYS A 75 -6.020 0.602 2.768 1.00 0.00 H

ATOM 1233 1HE LYS A 75 -5.182 -1.388 3.976 1.00 0.00 H

ATOM 1234 2HE LYS A 75 -6.749 -1.570 4.768 1.00 0.00 H

ATOM 1235 1HZ LYS A 75 -6.548 -3.001 2.871 1.00 0.00 H

ATOM 1236 2HZ LYS A 75 -7.739 -1.891 2.606 1.00 0.00 H

ATOM 1237 3HZ LYS A 75 -6.275 -1.723 1.867 1.00 0.00 H

ATOM 1238 N PHE A 76 -4.015 2.646 7.223 1.00 0.00 N

ATOM 1239 CA PHE A 76 -2.618 2.489 7.611 1.00 0.00 C

ATOM 1240 C PHE A 76 -2.406 1.203 8.399 1.00 0.00 C

ATOM 1241 O PHE A 76 -2.312 0.155 7.822 1.00 0.00 O

ATOM 1242 OXT PHE A 76 -2.333 1.237 9.596 1.00 0.00 O

ATOM 1243 CB PHE A 76 -2.158 3.686 8.446 1.00 0.00 C

ATOM 1244 CG PHE A 76 -0.702 3.647 8.811 1.00 0.00 C

ATOM 1245 CD1 PHE A 76 0.262 4.108 7.926 1.00 0.00 C

ATOM 1246 CD2 PHE A 76 -0.291 3.148 10.038 1.00 0.00 C

ATOM 1247 CE1 PHE A 76 1.603 4.072 8.260 1.00 0.00 C

ATOM 1248 CE2 PHE A 76 1.048 3.112 10.375 1.00 0.00 C

ATOM 1249 CZ PHE A 76 1.996 3.575 9.484 1.00 0.00 C

ATOM 1250 H PHE A 76 -4.736 2.256 7.813 1.00 0.00 H

ATOM 1251 HA PHE A 76 -2.011 2.441 6.707 1.00 0.00 H

ATOM 1252 1HB PHE A 76 -2.347 4.607 7.896 1.00 0.00 H

ATOM 1253 2HB PHE A 76 -2.738 3.731 9.367 1.00 0.00 H

ATOM 1254 HD1 PHE A 76 -0.049 4.503 6.958 1.00 0.00 H

ATOM 1255 HD2 PHE A 76 -1.040 2.782 10.741 1.00 0.00 H

ATOM 1256 HE1 PHE A 76 2.349 4.438 7.555 1.00 0.00 H

ATOM 1257 HE2 PHE A 76 1.357 2.719 11.343 1.00 0.00 H

ATOM 1258 HZ PHE A 76 3.052 3.546 9.748 1.00 0.00 H

TER
